# Supplementary material for: Finding the best fit: examining the decision-making of augmentative and alternative communication professionals in the UK using a discrete choice experiment
Source: BMJ Open. 2019 Dec 1;9(11):e030274. doi: 10.1136/bmjopen-2019-030274 (PMC6924798; doi:10.1136/bmjopen-2019-030274)
Supplement: Supplementary data [file bmjopen-2019-030274supp001.pdf]

Appendix A – Attributes from best-worst scaling case 1 study

Table B 1: Attributes used in best-worst scaling case 1 survey in Webb et al. [23] and rank in terms of relative importance score

Child attribute	Rank
*Child's receptive and expressive language abilities	1
Support for AAC from communication partners	2
*Communication ability with aided AAC	3
*Child's determination and persistence	4
Physical abilities for access	5
*Predicted future needs and abilities	6
Level of learning ability	7
Insight into own communicative skills	8
Attention level	9
Access to professional AAC support	10
Speech skills and intelligibility	11
Functional visual skills	12
History of aided AAC use	13
Presence of additional diagnoses	14
Level of fatigue	15
Literacy ability	16
Educational stage	17
Primary diagnosis	18
Mobility	19
**AAC system attributes**	**Rank**
*Vocabulary or language package(s)	1
*Consistency of layout and navigation	2
Ease of customization	3
Durability and reliability	4
*Type of vocabulary organization	5
Number of key presses required to generate symbol or text output	6
*Size of output vocabulary	7
Range of access methods	8
Number of cells per page	9
Portability	10
*Graphic representation	11
Battery life	12
Supplier support	13
Ease of mounting on a range of equipment	14
Cost	15
Additional assistive technology functions	16
Voice	17
Appearance	18

Note. Asterisk indicates attribute included in discrete choice experiment.

Appendix B – Example survey

Note: the AAC system levels and child vignettes shown here are for illustrative purposes and do not represent the statistical design used in the full survey.

Instructions

Thank you for taking part in this survey.

It aims to identify what factors clinicians think are important when making decisions about aided AAC systems for children with communication difficulties.

You will be asked a series of questions. Each one has the same format. A brief description of a child will be given, along with three possible choices of aided AAC systems.

The three AAC systems are described in terms of five characteristics (the systems are identical apart from changes to these five characteristics):-

1. **Vocabulary sets**: Pre-determined vocabulary or language package provided, which can be:-
   - No commercially provided sets
   - Commercially provided sets without language progression
   - Commercially provided sets with language progression
2. **Size of vocabulary**: The size of the output vocabulary available within the aided AAC system, which can be:-
   - Up to 50 vocabulary items
   - 50-1000 vocabulary items
   - More than 1000 vocabulary items
3. **Type of vocabulary organisation**: Primary format used to organise the vocabulary within the aided AAC system, which can be:-
   - Visual scene display
   - Semantic organisation
   - Semantic syntactic organisation
   - Pragmatic organisation

4. **Graphic Representation**: Primary type of graphic symbol used, which can be:-
   - Photo symbols (i.e. a photo symbol set without rules or encoding)
   - Pictographic symbols (i.e. a graphic symbol set without rules or encoding)
   - Ideographic symbols (i.e. a symbol system with rules or encoding)
   - Graphic symbols with text (i.e. a system with either pictographic or ideographic symbols that incorporates an alphabet for generating text)
5. **Consistency of layout**: Consistency of layout of symbols on pages, including when navigating through pages to select desired output, which can be:-
   - Inconsistent layout
   - Somewhat consistent layout
   - Highly consistent layout

Imagine you had to choose between only these three systems. You should indicate which you would prescribe for the child described. If your preferred option is not available, pick the system from the three options that you think best matches the child's needs. There are no right or wrong answers. It is acknowledged that this may feel uncomfortable for you.

In the survey, there are three different children described. You will be asked four questions about each child (12 questions in total).

In acknowledgement of choices being uncomfortable, after each choice, you will be asked to indicate how well you think that system matches the child's needs. (1 = very unsuitable, 7 = very suitable).

This survey is part of independent research funded by the National Institute for Health Research (NIHR), Health Service and Delivery Research (HS&DR) Programme 14/70/153. The views expressed are those of the authors and do not necessarily reflect those of the NHS, the NIHR, NETSCC, the HS&DR programme or the Department of Health.

Consent

Your participation in this survey is voluntary. All information is collected anonymously and held in confidence. We hope you complete the survey but you are free to stop responding at any point resulting in your answers will be removed.

❑ I have read and understood the above and consent to taking part.

I confirm my work involves assessing children for aided AAC systems and I contribute to the decision making in relation to the language and vocabulary organisation within aided AAC systems.

❑ Yes

❑ No

If yes go to DCE questions.

If no go to a page with the following:-

Thank you for your interest in this survey. At present we are only recruiting participants who contribute to decision making in relation to the language and vocabulary organisation within aided AAC for children. Over the coming 12 months we will be recruiting people with a wider range of AAC experience to test decision making resources we are developing. If you are interested in this aspect of the project or would like to be notified when the free resources are available, there will be an opportunity at the end to submit your email address.

We would still like to ask you a few questions about your experience with AAC to check the representativeness of participants.

Then go directly to demographics questionnaire.

Question 1

Child A has delayed expressive and receptive language and is able to use aided AAC for a few communicative functions. Child A is motivated to communicate through symbol communication systems. Child A is predicted to regress in skills and abilities (regression).

	System 1	System 2	System 3
**Size of vocabulary**<br>The size of the output vocabulary available within the aided AAC system.	50-1000 vocabulary items	50-1000 vocabulary items	50-1000 vocabulary items
**Type of vocabulary organisation**<br>Primary format used to organise the vocabulary within the aided AAC system	Pragmatic	Visual Scene	Visual Scene
**Vocabulary sets**<br>Pre-determined vocabulary or language package provided	No vocabulary set	Vocabulary sets with staged progression	Fixed vocabulary set
**Graphic representation**<br>Primary type of graphic symbol used	Ideographic symbol system (with rules or encoding)	Photos	Ideographic symbol system (with rules or encoding)
**Type of vocabulary organisation**<br>Primary format used to organise the vocabulary within the aided AAC system	Idiosyncratic layout	Idiosyncratic layout	Consistency of some aspects of layout

For this child I would choose:

- ❑ System 1
- ❑ System 2
- ❑ System 3

On a scale from 1 to 7, how good a match is your chosen device for this child? (1=very unsuitable, 7=very suitable).

❑	❑	❑	❑	❑	❑	❑
1	2	3	4	5	6	7

Question 2

Child A has delayed expressive and receptive language and is able to use aided AAC for a few communicative functions. Child A is motivated to communicate through symbol communication systems. Child A is predicted to regress in skills and abilities (regression).

	System 1	System 2	System 3
**Size of vocabulary** The size of the output vocabulary available within the aided AAC system.	50-1000 vocabulary items	50-1000 vocabulary items	50-1000 vocabulary items
**Type of vocabulary organisation** Primary format used to organise the vocabulary within the aided AAC system	Taxonomic	Visual Scene	Pragmatic
**Vocabulary sets** Pre-determined vocabulary or language package provided	Vocabulary sets with staged progression	No vocabulary set	Fixed vocabulary set
**Graphic representation** Primary type of graphic symbol used	Text	Pictographic symbol set	Photos
**Type of vocabulary organisation** Primary format used to organise the vocabulary within the aided AAC system	Consistency of some aspects of layout	Idiosyncratic layout	Consistency of some aspects of layout

For this child I would choose:

- ❑ System 1
- ❑ System 2
- ❑ System 3

On a scale from 1 to 7, how good a match is your chosen device for this child? (1=very unsuitable, 7=very suitable).

❑	❑	❑	❑	❑	❑	❑
1	2	3	4	5	6	7

Question 3

Child A has delayed expressive and receptive language and is able to use aided AAC for a few communicative functions. Child A is motivated to communicate through symbol communication systems. Child A is predicted to regress in skills and abilities (regression).

	**System 1**	**System 2**	**System 3**
**Size of vocabulary** The size of the output vocabulary available within the aided AAC system.	50-1000 vocabulary items	50-1000 vocabulary items	Up to 50 vocabulary items
**Type of vocabulary organisation** Primary format used to organise the vocabulary within the aided AAC system	Semantic-Syntactic	Semantic-Syntactic	Visual Scene
**Vocabulary sets** Pre-determined vocabulary or language package provided	Vocabulary sets with staged progression	No vocabulary set	Fixed vocabulary set
**Graphic representation** Primary type of graphic symbol used	Text	Photos	Pictographic symbol set
**Type of vocabulary organisation** Primary format used to organise the vocabulary within the aided AAC system	Consistency of all aspects of layout	Idiosyncratic layout	Consistency of some aspects of layout

For this child I would choose:

- ❑ System 1
- ❑ System 2
- ❑ System 3

On a scale from 1 to 7, how good a match is your chosen device for this child? (1=very unsuitable, 7=very suitable).

❑	❑	❑	❑	❑	❑	❑
1	2	3	4	5	6	7

Question 4

Child A has delayed expressive and receptive language and is able to use aided AAC for a few communicative functions. Child A is motivated to communicate through symbol communication systems. Child A is predicted to regress in skills and abilities (regression).

	**System 1**	**System 2**	**System 3**
**Size of vocabulary** The size of the output vocabulary available within the aided AAC system.	50-1000 vocabulary items	50-1000 vocabulary items	50-1000 vocabulary items
**Type of vocabulary organisation** Primary format used to organise the vocabulary within the aided AAC system	Visual Scene	Taxonomic	Pragmatic
**Vocabulary sets** Pre-determined vocabulary or language package provided	Vocabulary sets with staged progression	Fixed vocabulary set	Fixed vocabulary set
**Graphic representation** Primary type of graphic symbol used	Ideographic symbol system (with rules or encoding)	Text	Photos
**Type of vocabulary organisation** Primary format used to organise the vocabulary within the aided AAC system	Consistency of some aspects of layout	Consistency of all aspects of layout	Idiosyncratic layout

For this child I would choose:
- ❑ System 1
- ❑ System 2
- ❑ System 3

On a scale from 1 to 7, how good a match is your chosen device for this child? (1=very unsuitable, 7=very suitable).

❑	❑	❑	❑	❑	❑	❑
1	2	3	4	5	6	7

Question 5

Child B has receptive language exceeding expressive language and no previous AAC experience. Child B is only motivated to communicate through methods other than symbol communication systems. Child B is predicted to maintain current skills and abilities (plateau).

	System 1	System 2	System 3
**Size of vocabulary**<br>The size of the output vocabulary available within the aided AAC system.	Up to 50 vocabulary items	50-1000 vocabulary items	More than 1000 vocabulary items
**Type of vocabulary organisation**<br>Primary format used to organise the vocabulary within the aided AAC system	Taxonomic	Visual Scene	Pragmatic
**Vocabulary sets**<br>Pre-determined vocabulary or language package provided	No vocabulary set	Fixed vocabulary set	Vocabulary sets with staged progression
**Graphic representation**<br>Primary type of graphic symbol used	Photos	Text	Ideographic symbol system (with rules or encoding)
**Type of vocabulary organisation**<br>Primary format used to organise the vocabulary within the aided AAC system	Consistency of all aspects of layout	Idiosyncratic layout	Consistency of some aspects of layout

For this child I would choose:

- ❑ System 1
- ❑ System 2
- ❑ System 3

On a scale from 1 to 7, how good a match is your chosen device for this child? (1=very unsuitable, 7=very suitable).

❑	❑	❑	❑	❑	❑	❑
1	2	3	4	5	6	7

Question 6

Child B has receptive language exceeding expressive language and no previous AAC experience. Child B is only motivated to communicate through methods other than symbol communication systems. Child B is predicted to maintain current skills and abilities (plateau).

	**System 1**	**System 2**	**System 3**
**Size of vocabulary**<br>The size of the output vocabulary available within the aided AAC system.	More than 1000 vocabulary items	Up to 50 vocabulary items	50-1000 vocabulary items
**Type of vocabulary organisation**<br>Primary format used to organise the vocabulary within the aided AAC system	Semantic-Syntactic	Semantic-Syntactic	Taxonomic
**Vocabulary sets**<br>Pre-determined vocabulary or language package provided	Vocabulary sets with staged progression	Fixed vocabulary set	No vocabulary set
**Graphic representation**<br>Primary type of graphic symbol used	Ideographic symbol system (with rules or encoding)	Photos	Ideographic symbol system (with rules or encoding)
**Type of vocabulary organisation**<br>Primary format used to organise the vocabulary within the aided AAC system	Idiosyncratic layout	Consistency of some aspects of layout	Consistency of some aspects of layout

For this child I would choose:

- ❑ System 1
- ❑ System 2
- ❑ System 3

On a scale from 1 to 7, how good a match is your chosen device for this child? (1=very unsuitable, 7=very suitable).

❑	❑	❑	❑	❑	❑	❑
1	2	3	4	5	6	7

Question 7

Child B has receptive language exceeding expressive language and no previous AAC experience. Child B is only motivated to communicate through methods other than symbol communication systems. Child B is predicted to maintain current skills and abilities (plateau).

	**System 1**	**System 2**	**System 3**
**Size of vocabulary**<br>The size of the output vocabulary available within the aided AAC system.	Up to 50 vocabulary items	More than 1000 vocabulary items	50-1000 vocabulary items
**Type of vocabulary organisation**<br>Primary format used to organise the vocabulary within the aided AAC system	Taxonomic	Pragmatic	Taxonomic
**Vocabulary sets**<br>Pre-determined vocabulary or language package provided	Fixed vocabulary set	Vocabulary sets with staged progression	No vocabulary set
**Graphic representation**<br>Primary type of graphic symbol used	Photos	Ideographic symbol system (with rules or encoding)	Text
**Type of vocabulary organisation**<br>Primary format used to organise the vocabulary within the aided AAC system	Consistency of all aspects of layout	Consistency of some aspects of layout	Idiosyncratic layout

For this child I would choose:

- ❑ System 1
- ❑ System 2
- ❑ System 3

On a scale from 1 to 7, how good a match is your chosen device for this child? (1=very unsuitable, 7=very suitable).

❑	❑	❑	❑	❑	❑	❑
1	2	3	4	5	6	7

Question 8

Child B has receptive language exceeding expressive language and no previous AAC experience. Child B is only motivated to communicate through methods other than symbol communication systems. Child B is predicted to maintain current skills and abilities (plateau).

	**System 1**	**System 2**	**System 3**
**Size of vocabulary**<br>The size of the output vocabulary available within the aided AAC system.	Up to 50 vocabulary items	More than 1000 vocabulary items	50-1000 vocabulary items
**Type of vocabulary organisation**<br>Primary format used to organise the vocabulary within the aided AAC system	Pragmatic	Semantic-Syntactic	Visual Scene
**Vocabulary sets**<br>Pre-determined vocabulary or language package provided	Fixed vocabulary set	Fixed vocabulary set	Vocabulary sets with staged progression
**Graphic representation**<br>Primary type of graphic symbol used	Ideographic symbol system (with rules or encoding)	Ideographic symbol system (with rules or encoding)	Photos
**Type of vocabulary organisation**<br>Primary format used to organise the vocabulary within the aided AAC system	Consistency of some aspects of layout	Consistency of all aspects of layout	Idiosyncratic layout

For this child I would choose:
- ❑ System 1
- ❑ System 2
- ❑ System 3

On a scale from 1 to 7, how good a match is your chosen device for this child? (1=very unsuitable, 7=very suitable).

❑	❑	❑	❑	❑	❑	❑
1	2	3	4	5	6	7

Question 9

Child C has delayed expressive and receptive language and no previous AAC experience. Child C is only motivated to communicate through methods other than symbol communication systems. Child C is predicted to maintain current skills and abilities (plateau).

	**System 1**	**System 2**	**System 3**
**Size of vocabulary** The size of the output vocabulary available within the aided AAC system.	50-1000 vocabulary items	More than 1000 vocabulary items	Up to 50 vocabulary items
**Type of vocabulary organisation** Primary format used to organise the vocabulary within the aided AAC system	Visual Scene	Pragmatic	Semantic-Syntactic
**Vocabulary sets** Pre-determined vocabulary or language package provided	Fixed vocabulary set	Fixed vocabulary set	Fixed vocabulary set
**Graphic representation** Primary type of graphic symbol used	Pictographic symbol set	Pictographic symbol set	Text
**Type of vocabulary organisation** Primary format used to organise the vocabulary within the aided AAC system	Idiosyncratic layout	Consistency of some aspects of layout	Consistency of all aspects of layout

For this child I would choose:
- ❑ System 1
- ❑ System 2
- ❑ System 3

On a scale from 1 to 7, how good a match is your chosen device for this child? (1=very unsuitable, 7=very suitable).

❑	❑	❑	❑	❑	❑	❑
1	2	3	4	5	6	7

Question 10

Child C has delayed expressive and receptive language and no previous AAC experience. Child C is only motivated to communicate through methods other than symbol communication systems. Child C is predicted to maintain current skills and abilities (plateau).

	System 1	System 2	System 3
**Size of vocabulary**<br>The size of the output vocabulary available within the aided AAC system.	Up to 50 vocabulary items	More than 1000 vocabulary items	Up to 50 vocabulary items
**Type of vocabulary organisation**<br>Primary format used to organise the vocabulary within the aided AAC system	Taxonomic	Semantic-Syntactic	Taxonomic
**Vocabulary sets**<br>Pre-determined vocabulary or language package provided	No vocabulary set	Vocabulary sets with staged progression	No vocabulary set
**Graphic representation**<br>Primary type of graphic symbol used	Ideographic symbol system (with rules or encoding)	Ideographic symbol system (with rules or encoding)	Photos
**Type of vocabulary organisation**<br>Primary format used to organise the vocabulary within the aided AAC system	Consistency of all aspects of layout	Consistency of some aspects of layout	Idiosyncratic layout

For this child I would choose:

- ❑ System 1
- ❑ System 2
- ❑ System 3

On a scale from 1 to 7, how good a match is your chosen device for this child? (1=very unsuitable, 7=very suitable).

❑	❑	❑	❑	❑	❑	❑
1	2	3	4	5	6	7

Question 11

Child C has delayed expressive and receptive language and no previous AAC experience. Child C is only motivated to communicate through methods other than symbol communication systems. Child C is predicted to maintain current skills and abilities (plateau).

	**System 1**	**System 2**	**System 3**
**Size of vocabulary**<br>The size of the output vocabulary available within the aided AAC system.	Up to 50 vocabulary items	50-1000 vocabulary items	50-1000 vocabulary items
**Type of vocabulary organisation**<br>Primary format used to organise the vocabulary within the aided AAC system	Visual Scene	Semantic-Syntactic	Semantic-Syntactic
**Vocabulary sets**<br>Pre-determined vocabulary or language package provided	No vocabulary set	Vocabulary sets with staged progression	No vocabulary set
**Graphic representation**<br>Primary type of graphic symbol used	Photos	Pictographic symbol set	Text
**Type of vocabulary organisation**<br>Primary format used to organise the vocabulary within the aided AAC system	Idiosyncratic layout	Consistency of some aspects of layout	Consistency of all aspects of layout

For this child I would choose:
- ❑ System 1
- ❑ System 2
- ❑ System 3

On a scale from 1 to 7, how good a match is your chosen device for this child? (1=very unsuitable, 7=very suitable).

❑	❑	❑	❑	❑	❑	❑
1	2	3	4	5	6	7

Question 12

Child C has delayed expressive and receptive language and no previous AAC experience. Child C is only motivated to communicate through methods other than symbol communication systems. Child C is predicted to maintain current skills and abilities (plateau).

	System 1	System 2	System 3
**Size of vocabulary** The size of the output vocabulary available within the aided AAC system.	Up to 50 vocabulary items	More than 1000 vocabulary items	50-1000 vocabulary items
**Type of vocabulary organisation** Primary format used to organise the vocabulary within the aided AAC system	Semantic-Syntactic	Semantic-Syntactic	Visual Scene
**Vocabulary sets** Pre-determined vocabulary or language package provided	No vocabulary set	Vocabulary sets with staged progression	Fixed vocabulary set
**Graphic representation** Primary type of graphic symbol used	Ideographic symbol system (with rules or encoding)	Text	Photos
**Type of vocabulary organisation** Primary format used to organise the vocabulary within the aided AAC system	Consistency of all aspects of layout	Idiosyncratic layout	Consistency of some aspects of layout

For this child I would choose:
- ❑ System 1
- ❑ System 2
- ❑ System 3

On a scale from 1 to 7, how good a match is your chosen device for this child? (1=very unsuitable, 7=very suitable).

❑	❑	❑	❑	❑	❑	❑
1	2	3	4	5	6	7

Questionnaire

In this final part of the survey, we'd like to ask you for some information about yourself and your experience with AAC.

We would like to know about the characteristics of the people who complete this survey to check that we have a representative sample. We would also like to check if people with different professional experiences have different opinions.

All responses will be held anonymously and we have no way of tracing your responses back to you as an individual.

Q. What is your age? _______ years.

Q. What is your gender?

❑ Male ❑ Female ❑ Other ❑ Prefer not to say

Q. How would you describe your ethnicity?

- ❑ White - English/Welsh/Scottish/Northern Irish/British
- ❑ White -Irish
- ❑ White - Gypsy or Irish Traveller
- ❑ White - Any other White background
- ❑ Mixed/Multiple ethnic group - White and Black Caribbean
- ❑ Mixed/Multiple ethnic group - White and Black African
- ❑ Mixed/Multiple ethnic group - White and Asian
- ❑ Mixed/Multiple ethnic group - Any other Mixed/Multiple ethnic background
- ❑ Asian/Asian British - Indian
- ❑ Asian/Asian British - Pakistani
- ❑ Asian/Asian British – Bangladeshi
- ❑ Asian/Asian British - Chinese
- ❑ Asian/Asian British - Any other Asian background
- ❑ Black/ African/Caribbean/Black British - African

- ❑ Black/ African/Caribbean/Black British - Caribbean
- ❑ Black/ African/Caribbean/Black British - Any other Black/African/Caribbean background
- ❑ Arab
- ❑ Other

Q. For how many years have you worked with AAC? _______ years.

Q. What is your professional background? You may select more than one option if applicable.

- ❑ Occupational therapist
- ❑ Speech and language therapist
- ❑ Assistive technology specialist
- ❑ Clinical scientist
- ❑ Teacher
- ❑ Other

Q. If you selected Other, please specify.

____

Q. How much of your role relates to AAC? _______%.

(e.g. 1 day per week = 20%, 2 days a week = 40%, etc.)

Q. How would you characterise your role? Pick the one that best describes your role.

- ❑ I refer on anyone who may benefit from AAC
- ❑ I assess and implement AAC. I seek support from within my own team for decisions made
- ❑ I assess and implement AAC. I seek support from outside my own team for
- ❑ decisions made
- ❑ I assess and implement AAC. I act as a support for others in relation to AAC
- ❑ decision making
- ❑ I assess only. I provide support to others outside my team in relation to
- ❑ AAC decision making
- ❑ Other

Q. If you selected Other, please specify.

____

Q. Out of the list below, select the three most common diagnoses you encounter in your work.

- ❑ Autism Spectrum Disorder
- ❑ Physical disability (e.g. neuromuscular, cerebral palsy etc.)
- ❑ Dyspraxia
- ❑ Intellectual Disability/Developmental Delay
- ❑ Neurological
- ❑ Specific Speech/Language Impairment
- ❑ Syndromes
- ❑ Unknown
- ❑ Other

Q. If you selected Other, please specify.

____

Q. Who do you provide services for? (Please choose all that apply.)

- ❑ All age groups
- ❑ Preschool age
- ❑ Primary school age
- ❑ Secondary school age
- ❑ Higher education
- ❑ Further education
- ❑ Adults
- ❑ Other

Q. If you selected Other, please specify.

____

Q. What is the geographical area covered by your service? (Please choose all that apply.

- ❑ North West England
- ❑ North East England
- ❑ Yorkshire and Humber
- ❑ West Midlands
- ❑ East Midlands
- ❑ East of England

- ❑ South West England
- ❑ South East England
- ❑ London
- ❑ Northern Ireland
- ❑ North Wales
- ❑ South Wales
- ❑ Mid-Wales
- ❑ Southern Scotland
- ❑ Central Scotland
- ❑ Northern Scotland
- ❑ Non-UK

End of survey

Thank you for your participation in this survey.

Your responses will contribute to the results of the I-ASC project and support the development of decision making resources for use in AAC assessments.

You can follow the progress of our research project on our website, on Facebook or on Twitter.

Figure A 1: Example discrete choice experiment task

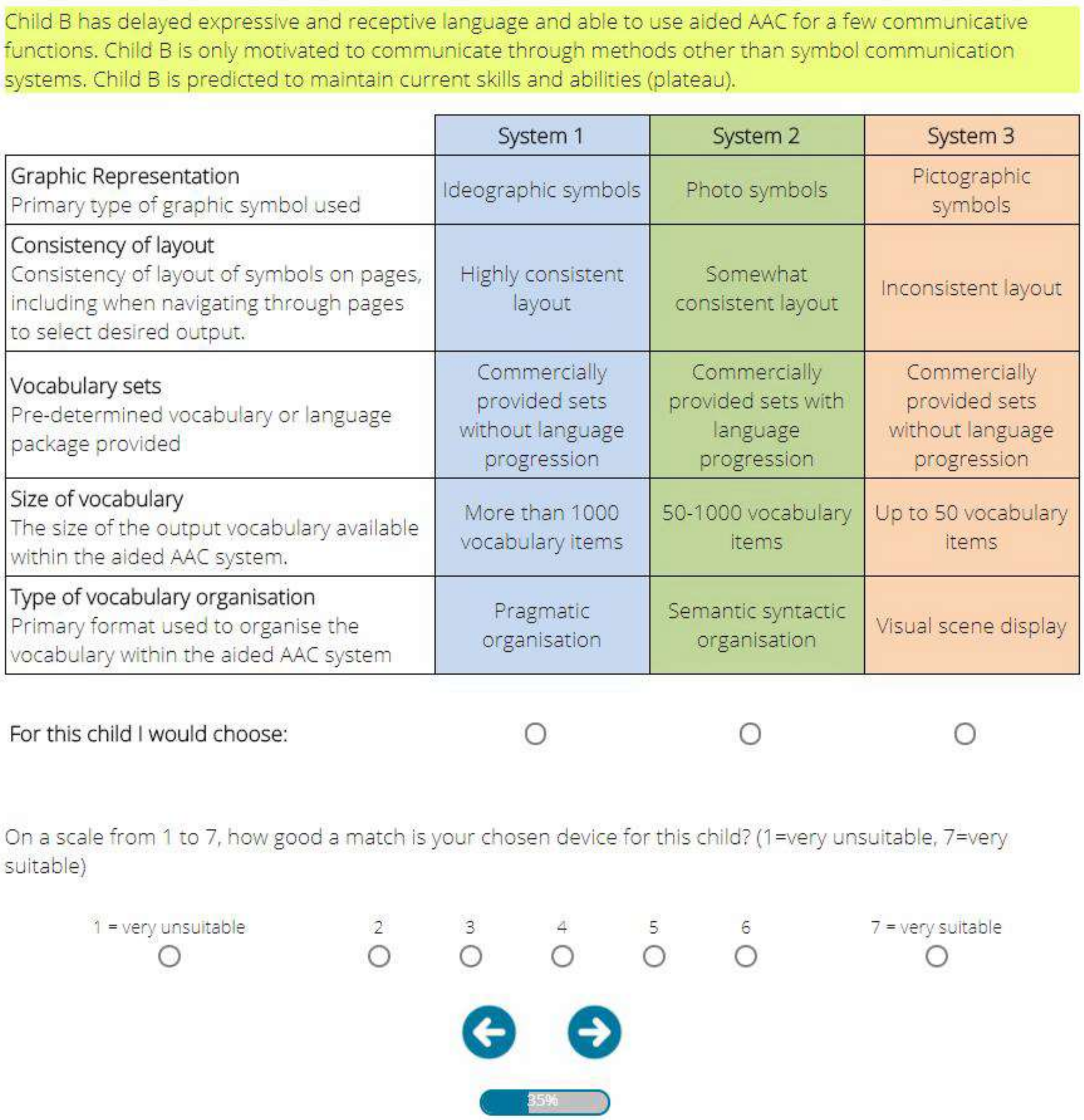

Child B has delayed expressive and receptive language and able to use aided AAC for a few communicative functions. Child B is only motivated to communicate through methods other than symbol communication systems. Child B is predicted to maintain current skills and abilities (plateau).

	System 1	System 2	System 3
Graphic Representation<br>Primary type of graphic symbol used	Ideographic symbols	Photo symbols	Pictographic symbols
Consistency of layout<br>Consistency of layout of symbols on pages, including when navigating through pages to select desired output.	Highly consistent layout	Somewhat consistent layout	Inconsistent layout
Vocabulary sets<br>Pre-determined vocabulary or language package provided	Commercially provided sets without language progression	Commercially provided sets with language progression	Commercially provided sets without language progression
Size of vocabulary<br>The size of the output vocabulary available within the aided AAC system.	More than 1000 vocabulary items	50-1000 vocabulary items	Up to 50 vocabulary items
Type of vocabulary organisation<br>Primary format used to organise the vocabulary within the aided AAC system	Pragmatic organisation	Semantic syntactic organisation	Visual scene display

For this child I would choose:

On a scale from 1 to 7, how good a match is your chosen device for this child? (1=very unsuitable, 7=very suitable)

1 = very unsuitable 2 3 4 5 6 7 = very suitable

35%

Appendix C – Final preferred model selection process

A full model with all interaction terms and two alternative specific constants implies 98 parameters, which is too many to reliably estimate given the amount of data collected and given that many interactions are expected to be of very low magnitude. Thus, a strategy was required to identify a suitable model with fewer parameters.

The first stage was estimating a series of stepwise multinomial logit (MNL) models, beginning with a model with all 98 parameters. The parameter with the highest p-value, excluding the γ_0 constant terms, was eliminated, and a model with 97 parameters was estimated. Then the parameter with the lowest p-value was excluded and a new model run, and so on in an iterative process until only the 12 γ_0 constant terms remained (one for each non-baseline system level).

The Bayesian Information Criterion (BIC) was used to select the preferred MNL model. This model was then re-estimated as a mixed logit (MIXL) model to account for participant heterogeneity. (The process did not begin by estimating a series of stepwise MIXL models due to the difficulty and greatly increased computational resources required to estimate MIXL models with a large number of parameters.) The β \coefficients on system attribute levels were assumed to be drawn from normal distributions with means given by

$$\bar{\beta}_{alc} = \gamma_{al0} + \gamma_{al} z_c$$

and variances given by

$$\sigma_{alc}^2 = (\sigma_{al0} + \sigma_{al} z_c)^2.$$

If p is the number of parameters of the preferred MNL model, then models with between p – 3 and p + 3 parameters were re-estimated as MIXL models. The BIC for each MIXL model is given in **Error! Reference source not found.**.

The MIXL model minimising the BIC was chosen as the final preferred model.

Table C 1: Bayesian information criteria (BIC) for estimated mixed logit models

Number of parameters	BIC
22	3502.25
23	3487.80
24	3482.30
25	3489.18
26	3493.07
27	3502.28
28	3509.34

Appendix D – Relative interaction attribute importance

Relative information attribute importance (RIAI) measures the amount that preferences for attributes of choice objects are impacted by a given interaction attribute associated with a choice situation relative to other interaction attributes. It may be calculated either with respect to a single choice object attribute or overall with respect to all choice object attributes.

RIAI is calculated with respect to a single choice object attribute by taking the difference between the greatest increase an interaction attribute causes to a choice object attribute's part worth utility and the greatest decrease, expressed as a percentage of the differences for all interaction attributes. Formally, the RIAI for interaction attribute i with respect to choice attribute c is

$$RIAI_{ic} = 100\left(\frac{\gamma_{ic}^{max} - \gamma_{ic}^{min}}{\sum_{j=1}^{N_I} \gamma_{jc}^{max} - \gamma_{jc}^{min}}\right)$$

where γ_{ic}^{max} and γ_{ic}^{min} are respectively the maximum and minimum coefficients for interaction attribute i with respect to choice attribute c and N_I is the number of interaction attributes. The overall RIAI for i is similarly calculated as

$$RIAI_{i} = 100\left(\frac{\gamma_{i}^{max} - \gamma_{i}^{min}}{\sum_{j=1}^{N_I} \gamma_{j}^{max} - \gamma_{j}^{min}}\right)$$

Where now γ_{i}^{max} and γ_{i}^{min} are respectively the maximum and minimum coefficients for interaction attribute i across all choice attributes.
