# Supplementary material for: Finding the best fit: examining the decision-making of augmentative and alternative communication professionals in the UK using a discrete choice experiment
Source: BMJ Open. 2019 Dec 1;9(11):e030274. doi: 10.1136/bmjopen-2019-030274 (PMC6924798; doi:10.1136/bmjopen-2019-030274)
Supplement: Author's manuscript [file bmjopen-2019-030274.draft_revisions.pdf]

BMJ Open

BMJ Open is committed to open peer review. As part of this commitment we make the peer review history of every article we publish publicly available.

When an article is published we post the peer reviewers' comments and the authors' responses online. We also post the versions of the paper that were used during peer review. These are the versions that the peer review comments apply to.

The versions of the paper that follow are the versions that were submitted during the peer review process. They are not the versions of record or the final published versions. They should not be cited or distributed as the published version of this manuscript.

BMJ Open is an open access journal and the full, final, typeset and author-corrected version of record of the manuscript is available on our site with no access controls, subscription charges or pay-per-view fees (http://bmjopen.bmj.com).

If you have any questions on BMJ Open's open peer review process please email info.bmjopen@bmj.com

BMJ Open

Finding the best fit: A discrete choice experiment on the decision making of augmentative and alternative communication professionals

Journal:	*BMJ Open*
Manuscript ID	bmjopen-2019-030274
Article Type:	Research
Date Submitted by the Author:	06-Mar-2019

Complete List of Authors:	Webb, Edward; University of Leeds, Leeds Institute of Health Sciences<br>Meads, David; University of Leeds, Leeds Institute of Health Sciences<br>Lynch, Yvonne; Manchester Metropolitan University, Faculty of Health, Psychology and Social Care<br>Randall, Nicola; Barnsley Hospital NHS Foundation Trust, Barnsley Assistive Technology Team<br>Judge, Simon; Barnsley Hospital NHS Foundation Trust, Barnsley Assistive Technology Team<br>Goldbart, Juliet; Manchester Metropolitan University, Faculty of Health, Psychology and Social Care<br>Meredith, Stuart; Manchester Metropolitan University, Faculty of Health, Psychology and Social Care<br>Moulam, Liz; Manchester Metropolitan University, Faculty of Health, Psychology and Social Care<br>Hess, Stephane; University of Leeds, Choice Modelling Centre and Institute of Transport Studies<br>Murray, Janice; Manchester Metropolitan University, Faculty of Health, Psychology and Social Care

Keywords:	discrete choice experiment, augmentative and alternative communication, clinical decision making

SCHOLARONE™
Manuscripts

Finding the best fit: A discrete choice experiment on the decision making of augmentative and alternative communication professionals

Edward J.D. Webb[1,2] David Meads[2,3] Yvonne Lynch[4,5]

Nicola Randall[6,7] Simon Judge[6,8] Juliet Goldbart[4,9] Stuart Meredith[4,10]

Liz Moulam[4,11] Stephane Hess[12,13] Janice Murray[4,14]

Keywords: discrete choice experiment; augmentative and alternative communication; clinical decision making

Word count: 4280

For peer review only

[1] Corresponding author; e.j.d.webb@leeds.ac.uk; +44 113 343 2982; Leeds Institute of Health Sciences (LIHS), Level 10 Worsley Building, Clarendon Way, Leeds, LS2 9NL, UK. OrcID: 0000-0001-7918-839X
[2] Leeds Institute of Health Sciences, University of Leeds
[3] d.meads@leeds.ac.uk
[4] Faculty of Health, Psychology and Social Care, Manchester Metropolitan University
[5] yvonne.lynch@mmu.ac.uk
[6] Barnsley Assistive Technology Team, Barnsley Hospital NHS Foundation Trust
[7] nicola.randall1@nhs.net
[8] simon.judge@nhs.net
[9] j.goldbart@mmu.ac.uk
[10] meredithsn@hotmail.com
[11] l.moulam@mmu.ac.uk
[12] Choice Modelling Centre and Institute for Transport Studies, University of Leeds
[13] s.hess@leeds.ac.uk
[14] j.murray@mmu.ac.uk

For peer review only - http://bmjopen.bmj.com/site/about/guidelines.xhtml

A DCE on AAC professionals' decision making

ABSTRACT

Objectives: Children with a wide range of conditions (e.g. cerebral palsy, autism) can benefit from augmentative and alternative communication (AAC) systems. However, little is known about professionals' decision making when recommending symbol based AAC systems for children. This study examines AAC professionals' preferences for attributes of AAC systems and how they interact with child characteristics.

Design: A DCE with six AAC system attributes and four child attributes. Participants chose which AAC system to provide for a child vignette.

Setting: An online survey in the UK.

Participants: 155 UK-based AAC professionals recruited between 20/10/17 and 4/3/18.

Outcomes: AAC professionals' preferences as measured by an optimal mixed logit model identified using a stepwise procedure and the Bayesian Information Criterion.

Results: Significant differences were observed in preferences for AAC system attributes and large interactions were seen between child attributes, e.g. motivation to communicate using AAC, and predicted progression in skills and abilities lead to participants making more ambitious choices for children. These characteristics were perceived as relatively more important than language ability and previous AAC experience.

Conclusions: It is possible to examine AAC professionals' decision making using a DCE. AAC professionals make trade-offs between attributes of AAC systems, and these trade-offs change depending on the characteristics of the child for whom the system is being provided.

STRENGTHS AND LIMITATIONS OF THIS STUDY

- This is the first discrete choice experiment, and only the second stated preferences study in the field of augmentative and alternative communication technology.

For peer review only - http://bmjopen.bmj.com/site/about/guidelines.xhtml

For peer review only

- The study used unusual and innovative methodology by (1) using a best-worst scaling case 1 study in attribute selection; (2) having AAC system choices be made in the context of a child vignette formed from a set of attributes; and (3) introducing a new measure termed relative interaction attribute importance to interpret results.
- Child vignettes were relatively simple, and a single vignette could represent children with very different needs.
- In some ways the discrete choice experiment task differed from how augmentative and alternative professionals make decisions in practice.

INTRODUCTION

Many people struggle to produce speech due to a wide range of condition, including cerebral palsy, intellectual/developmental delays and autism spectrum conditions. Even within diagnoses, individuals' communication related needs and abilities are extremely varied. Augmentative and alternative communication (AAC) refers to approaches which support the communication of such individuals. AAC systems encompass a large number of unaided methods including signing, facial expression and body language as well as the use of aided systems [1]. This article focuses on aided AAC systems, also known as communication aids, which include high-tech electronic devices, such as that used by Stephen Hawking or Britain's Got Talent winner Lee Ridley, as well as low-tech systems, such as boards and communication books.

AAC can improve the lives of people with communication disabilities [2-4]. AAC is especially important for children, as their language and communication abilities are developing and their needs evolving [5-7], in contrast to an adult with an acquired condition. What system they use thus has an impact not just in the immediate future but over their lifetime.

Choosing an AAC system requires consideration of many features. For example, what type of graphic symbols (e.g. photos, stylized pictures, words) to use, how many symbols are available, how they are organized, and how they are accessed.

For peer review only - http://bmjopen.bmj.com/site/about/guidelines.xhtml

For peer review only

The process through which children receive AAC systems can vary [8 9], but commonly their needs and abilities are assessed by a team of AAC professionals, which may include speech and language therapists, occupational therapists and/or specialist teachers [10]. The team makes recommendations, and a final decision is made with variable input from the child and family, depending on individual circumstances. The large degree of heterogeneity in the population of people who benefit from AAC, and in the systems available, means the assessment and subsequent matching of individual and system is a complex task and unique to each person.

It has been estimated that approximately 1 in 200 children in the UK would benefit from AAC [11-13], although rates of abandonment of AAC systems by children of 30-50% have been observed [14 15], with the causes of abandonment not well understood. AAC systems can be costly (up to £10,000 for high-tech systems) and require a large amount of professional support [16]. However, they have been suggested to be a cost-effective use of NHS resources [17].

There is currently a lack of documented evidence for assessment and decision making processes [18 19], and what does exist is frequently individual case studies [3,17]. AAC practitioners must often make difficult and complex decisions in a complicated, heterogeneous and rapidly evolving environment, balancing the needs of an individual child, and available resources, and take account of the cultural and contextual influences that shape each assessment [20 21]. While there are studies which highlight some important factors in decision making [12 18 22], available guidelines tend to focus on the organisational structure of services, rather than decision making as such [10 19]

This study is part of a wider project examining provision of AAC systems for children entitled I-ASC: Identifying appropriate symbol communication aids for children who are non-speaking. It has several components and has employed a number of research methods with the aim of generating a body of research evidence on current practice, recommendations for best practice, and resources to support AAC professionals in making these decisions. This study aims to contribute quantitative evidence regarding the current decision making rationale of AAC professionals.

For peer review only - http://bmjopen.bmj.com/site/about/guidelines.xhtml

As yet, there is limited quantitative research in AAC decision making. A study from the current research project ran a Best-worst Scaling (BWS) Case 1 survey [23], which quantified what AAC professionals considered the most and least important factors related to both children and AAC systems. However, the earlier study did not examine the trade-offs professionals make between different attributes of AAC systems, nor how trade-offs change depending on the characteristics of children.

This study presents a discrete choice experiment (DCE) which seeks to achieve both these aims. Participants were shown a series of vignettes describing hypothetical children and made choices as to which among a set of hypothetical AAC systems they would choose for each child. Analysing the results revealed respondents' preferences for the levels of various AAC system attributes, as well as how those preferences are influenced by the children's characteristics.

METHODS

Survey development

No stated preference work existed in AAC prior to this research project, meaning a large number of potential attributes and little evidence as to which to include in a DCE. Thus a BWS case 1 study was performed initially and the results used to guide attribute selection for the DCE. Attributes for the BWS study were created using focus groups and interviews with AAC professionals, people who use AAC, their families, and other stakeholders; systematic literature reviews; and input from an expert panel. For more details see section 2 of Webb et al. [23].

The BWS study produced relative importance scores for 19 child and 18 system attributes given in Appendix B with their rank in terms of relative importance. DCE attributes were selected from these during consensus discussions between authors with expertise in AAC, speech and language therapy, and health economics. The selection criteria were that attributes should: (1) form coherent and realistic descriptions of children and systems; (2) address the research aims of the wider research project; (3) include mainly attributes with high relative importance scores in the BWS study; and (4) be small in number so choice tasks

For peer review only - http://bmjopen.bmj.com/site/about/guidelines.xhtml

would not over burden respondents. Consensus was achieved via unstructured discussions until all authors were in agreement. This resulted in four child and five system attributes, listed in Tables 1 and 2 together with non-specialist descriptions.

Broadly speaking, child attributes capture a child's language ability, experience with AAC, attitude/motivation to communicate with AAC, and whether the child is expected to regress, plateau or progress in communication ability. System attributes broadly capture what vocabulary set(s) are pre-provided by manufacturers, how many vocabulary items are provided, how they are organised, the type of graphical symbols used, and how consistent the layout of words/symbols are.

A total of 54 vignettes can be formed from the child attributes. Authors with expertise in AAC and speech and language therapy identified and removed 18 vignettes representing unrealistic combinations, leaving 36. Each participant had three vignettes randomly selected to answer questions about.

Prior experience from the BWS study suggested it would be difficult to recruit a large respondent sample, thus a relatively heavy response burden of 12 choices between three systems was selected. Authors with experience in AAC and speech and language therapy removed 158 unrealistic combinations from the 432 AAC systems which could be formed from the system attributes, leaving 274. A D-efficient survey design was generated using NGene (©ChoiceMetrics) with five blocks, meaning 60 choice tasks in total. Random allocations of block and child vignettes were independent.

The children in the first, second and third vignettes were referred to as Child A, Child B and Child C respectively, and participants made four choices for each with choices for a given child grouped together. Note that as the sets of AAC systems presented were driven by the experimental design, participants were offered different choices for each child. The order of system attributes was randomised between participants, but consistent within participants and which systems appeared on the left, middle and right of the screen was also randomised. An example question is shown in Appendix A.

For peer review only - http://bmjopen.bmj.com/site/about/guidelines.xhtml

Participants finally answered questions about themselves and their experiences with AAC (for details, see supplementary online material). The DCE was administered online for ease of recruitment and was tested by five AAC professionals and the wording of some attributes and levels altered. Participants began by confirming they contributed towards AAC decision making for children, and those who did not answered only demographic questions. The precise wording of the question was: "I confirm my work involves assessing children for aided AAC systems and I contribute to the decision making in relation to the language and vocabulary organisation with in AAC systems." During testing it was revealed that some AAC professionals did not have sufficient input into the decision making process in their day-to-day practice for the DCE questions to be meaningful (e.g. occupational therapists specialising in optimising physical access to an AAC system recommended by other members of the team), and this question was designed to filter out such respondents.

Recruitment was carried out using email lists of AAC professionals gathered by the research project as part of prior activities, publically available lists and websites and the professional contacts of authors. The study was also advertised via the mailing list of Communication Matters (www.communicationmatters.org.uk), a UK wide AAC charity, through the project website and online media. Responses were collected between 20/10/17 and 4/3/18.

Analysis

Analysis of participants' choices was grounded in random utility maximisation. In a given choice scenario t, participant i chooses which of three AAC systems to allocate to child c. The utility to participant i of allocating AAC system $s \in \{1,2,3\}$ to child c in choice scenario t is

$$u_{isc} = \alpha_s + \beta_{ic} x_s + \varepsilon_i$$

where α_s is an alternative specific constant for AAC system s, x_s is a vector of dummy variables indicating AAC system levels, β_{ic} is a vector of coefficients which differ across participants and children, and ε_i is an error term which varies across choice scenarios and alternatives.

For peer review only - http://bmjopen.bmj.com/site/about/guidelines.xhtml

The coefficient on level l of system attribute a, β_{ialc}, depends of the characteristics of the child vignette according to

$$\beta_{ialc} = \gamma_{ial0} + \gamma_{ial} z_c$$

where γ_{ial0} is a constant giving the preference for a system attribute at baseline child levels, z_c is a vector of dummy variables indicating vignette levels and γ_{ial} is a vector of coefficients, allowing for heterogeneity in relative preference for AAC system attributes depending on child characteristics.

A full model with all interaction terms includes too many parameters to estimate reliably. Thus parameters were eliminated in a stepwise process and a final preferred mixed logit model identified using the Bayesian Information Criterion. The mixed logit model incorporates participant heterogeneity by allowing AAC system attribute parameters to be random, following a normal distribution with both means and variances depending on child characteristics. For details, see Appendix C.

Models were estimated using the CMC Choice Modelling Centre Code for R version 1.1 [24] and all analysis was carried out using R version 3.3.1. Statistical significance was assessed at the 5% level after adjusting for multiple testing using Holm's sequential Bonferroni correction [25].

Results are presented using a new measure termed *relative interaction attribute importance* (RIAI) which assesses how big an impact child attributes have on AAC professionals' decision making. It is analogous to relative attribute importance, often used to present DCE results [26], and it may be calculated either with respect to a single choice object attribute or overall with respect to all choice object attributes. For details, see Appendix D.

Note relative attribute importance is not an appropriate measure of the importance of AAC system attributes here, as their relative importance changes depending on which child AAC professionals are presented with. Nor is it appropriate to take the mean relative importance over all child vignettes. The set of child vignettes used is not representative of the case mixes seen by AAC professionals: some vignettes may represent children commonly seen, while other vignettes may represent a type of child seldom encountered. Thus

For peer review only - http://bmjopen.bmj.com/site/about/guidelines.xhtml

averaging over the set of vignettes would not give meaningful insight as to the relative importance of an attribute in AAC professionals' decision making in the real world.

Patient and public involvement

One author (SM) is an AAC user, and one (LM) is the parent of an AAC user, and both were involved in all stages of the research. DCE attributes were developed with impact from AAC stakeholders and the survey tested with AAC professionals as detailed above. Findings from the study and the wider research project have been disseminated to AAC stakeholders and the public at events at the Scottish Parliament (Edinburgh, UK), the Science and Industry Museum (Manchester, UK) and the Houses of Parliament (London, UK).

RESULTS

A total of 172 participants completed the survey, of whom 155 indicated they contributed to decision making regarding AAC systems and answered DCE questions. Summary statistics of their demographics and professional experience are given in Table 3. Most participants were female (~90%) and white. We believe this to be reasonably representative of the population of AAC professionals in the UK. The mean age of DCE participants was around 40 and they had around 10 years experience on average. Around 75% of DCE participants had a speech and language therapy background, with no other background reported by more than 10%. Those who did not answer DCE questions were less likely to have a speech and language therapy background (~50%), with teacher (~20%) and occupational therapist (~30%) more common.

Approximately 30% of the sample worked with all age groups, while 50-60% worked with each of pre-school, primary school and secondary school age children. The sample also encountered a wide range of diagnoses, e.g. physical disability (~80% of DCE participants), intellectual disability/developmental delay (~70%) and autism spectrum disorder (~65%).

Turning to DCE responses, respondents chose the left-hand option 37.6% of the time, and the central and right-hand options 33.1% and 29.2% of the time respectively, significantly different from an equal distribution (one sample Kolmogorov-Smirnov $p = 0.002$).

For peer review only - http://bmjopen.bmj.com/site/about/guidelines.xhtml

Table 4 contains the results of the final preferred model, with 24 coefficients. The "constant" terms give participants' preferences for AAC system allocation when shown a vignette with all attributes at baseline levels, which represents the most challenging profile that can be formed from the set of child levels. ("Child A/B/C has delayed expressive and receptive language and no previous AAC experience. Child A/B/C does not appear motivated to communicate through any methods and means. Child A/B/C is predicted to progress in skills and abilities (regression).") The interaction terms in the model hence represent how respondents' preferences for AAC systems change if choosing for a vignette presenting less of a challenge on a given child attribute.

For the baseline vignette, vocabulary sets which are fixed or have staged progression are preferred to no pre-provided vocabulary. There are no significant differences in preferences between up to 50 and 50-1000 vocab items, but over 1000 items is considered significantly worse. There is no significant preference between visual scene, taxonomic or semantic-syntactic vocabulary organisation, but pragmatic organisation is preferred. There is no preference between graphic representation using photos or pictographs, but text is less preferred than either, and idiographic symbols are considered even worse. Finally, having only some aspects of system layout consistent is less preferred than having all aspects consistent or an idiosyncratic layout.

Compared to this baseline vignette, professionals were much more likely (odds ratio, OR 3.88) to choose systems with staged progression vocabulary sets with staged progression to no pre-installed set if the vignette predicted progress in skills and ability. An intermediate number of vocabulary items (50-1000) became more preferable compared to 50 or fewer for a vignette motivated to communicate using AAC. Over 1000 items became significantly more preferable for vignettes with a variety of characteristics: receptive language exceeding expressive language, an ability to use a range of AAC functions, motivated to communicate using AAC and predicted to progress.

For peer review only - http://bmjopen.bmj.com/site/about/guidelines.xhtml

Two significant interactions exist between vocabulary organisation and motivation. A vignette with motivation to communicate using AAC became more likely to be allocated a system with taxonomic (OR 2.03), or semantic-syntactic (OR 2.29) organisation compared to visual scene.

Motivation to communicate using AAC also has a large influence on graphic representation. It increases the probability of choosing pictographic symbols (OR 3.88), idiographic symbols (OR 5.31) or text (OR 4.00) rather than photos. However, being predicted to progress makes pictographic symbols less preferable.

Figure 1 illustrates the RIAI of child attributes for each system attribute and overall. Consistency of layout is omitted, as there are no interactions for this attribute. Predicted future skills and abilities is the only child attribute to influence preferences for type of vocabulary set. It is also one of only two to influence preferences for graphic representation, although determination and persistence is more important (67% vs. 33%). Determination and persistence is the only child attribute to impact preferences for type of vocabulary organisation. All child attributes have an influence on preferences for vocabulary size, with communication ability with AAC (32%) and determination and persistence (28%) relatively more important than future skills and abilities (22%) and receptive and expressive language (17%). Overall, future skills and abilities has the greatest relative importance (38%), followed by determination and persistence (19%), communication ability with AAC (20%), and receptive and expressive language (12%).

DISCUSSION

This study has demonstrated the feasibility of DCEs as a research tool in AAC. Although some informal feedback was received that participants found the tasks difficult due to not having as much information or as wide a range of options as in real life, the AAC professionals that responded to the survey found the tasks meaningful. The set of AAC system attributes created coherent options and the child vignettes presented meaningful descriptions which conveyed useful information to participants.

This DCE has revealed AAC professionals' priorities when choosing AAC systems for children and shown that these priorities interact with children's, to the extent that for some system attributes their preferences for

For peer review only - http://bmjopen.bmj.com/site/about/guidelines.xhtml

For peer review only

different levels can completely reverse depending which vignette is shown. For example, for the baseline vignette, a system with more than 1000 vocab items is less likely to be chosen then one with less than 50 (OR 0.395). However, for a vignette with a receptive-expressive language gap, can use AAC for a range of functions, is motivated to use AAC and is predicted to progress, a system with more than 1000 vocab items is much more likely to be chosen (OR 22.5).

Overall, motivation to communicate with AAC has the greatest number of interactions with preferences. Motivation is also more important in terms of RIAI than language ability or previous experience with AAC, although motivation to communicate through non-AAC methods has no bearing on preferences in the final model. Motivation to communicate via AAC tended to drive participants towards what can be regarded as more "ambitious" choices, for example more vocabulary items.

Visual scene vocabulary organisation and graphic representation using photos can both involve items/scenes from an individual's own life and use literal, rather than abstract depictions. Both became less preferred for a vignette motivated to communicate via AAC, in favour of more abstract methods of organisation (taxonomic and semantic-syntactic) and graphic symbols that require more grammar (pictographs, ideographs and text). This may be interpreted as AAC professionals believing that motivated children will be better able to use more complex AAC systems. An alternative and by no means mutually exclusive interpretation is that lack of motivation requires an AAC system involving familiar cues from their everyday environment.

AAC system preferences did not significantly differ between vignettes with skills and abilities predicted to regress or plateau. This may be due to children predicted to regress not being commonly encountered. However, if a child is predicted to progress, this has a large impact on decision making, and future skills and abilities is the highest ranked attribute in terms of RIAI. As with motivation, it leads to more ambitious choices, with more vocab items preferred and pictographs depreciated as graphic symbols compared to ideographs and text. However, unless the vignette featured both predicted progress *and* motivation to

For peer review only

For peer review only - http://bmjopen.bmj.com/site/about/guidelines.xhtml

communicate via AAC, photos were still most preferred. This possibly indicates that they remain a good starting point for a child who is not engaged, regardless of prognosis.

Even in the light of potentially high rates of abandonment, AAC professionals have high expectations of motivated children expected to progress, even if their receptive and expressive language are both delayed and they have no previous AAC experience. This may be viewed in the light of official guidance [27] that AAC professionals should have such high expectations.

For many vignettes, there are non-linear preferences for vocabulary size. Between 50 and 1000 items was considered better than 50 or fewer for all child vignettes, although the difference was not always significant. This may indicate that AAC professionals do not wish to limit potential for expression, even for children with low ability and poor prognosis.

Respondents commonly preferred levels of AAC systems that can require a lot of personalisation, e.g. photographic graphic representation, pragmatic vocabulary organisation or an idiosyncratic layout. Such options require a lot of time and effort on behalf of AAC professionals, highlighting the need in real-world practice to allow sufficient time for AAC system setup. Another implication is that "off the shelf" AAC systems may not generally be suitable for children without alteration. Note that this is not necessarily a criticism of the range of AAC systems available from manufacturers.

The above results are potentially contrasted by preferences for vocabulary sets, where no pre-provided vocabulary set was always considered worse than having pre-provided sets that were either fixed or with staged progression. However, even here, AAC professionals may have intended to customise the provided sets to tailor them to the individual.

Comparing the DCE results with the previous BWS Case 1 study, some similarities may be observed. For example, graphic representation was selected as the wider research project aimed to investigate the properties of graphic symbols, and was the lowest ranked attribute in terms of importance in the BWS to be

For peer review only - http://bmjopen.bmj.com/site/about/guidelines.xhtml

included in the DCE. In concordance with this finding, if relative importance of AAC system attributes is calculated for each child vignette in the DCE, it is never the most important attribute.

Many differences can also be seen. Language abilities was the most important child attribute in the BWS, yet its RIAI in the DCE is below predicted future abilities, ranked sixth in the BWS. However, differences in results do not necessarily imply contradiction, as the two methodologies do not measure the same thing. The BWS measured the importance of AAC system attributes over the case mix AAC professionals encounter in practice, whereas for the DCE respondents were presented with a specific child vignette.

Limitations

This study has several limitations. As it was not possible to estimate a model with all interactions, results will to a certain extent be sensitive to the model selection strategy. However, the final model was selected using a well-established and widely used selection criterion (BIC). A larger sample size may have allowed robust estimation of more complex models, yet 155 participants represents a large proportion of the population of AAC professionals working in the UK, which is estimated at around 800 (Communication Matters, personal correspondence).

Respondents were more likely to choose AAC systems on the left of the screen and less likely to choose ones on the right, potentially introducing bias in estimated coefficients. However, alternative specific constants were included when modelling responses, and did not provide enough explanatory power to be included in the final model. In addition, the positions in which AAC systems were presented was randomised, mitigating any possible bias.

In some ways, the DCE task does not match how AAC professionals make decisions in practice. Typically, they work together with families and children, as well as part of an AAC team, which could include diverse areas of expertise. They also generally make recommendations, rather than unilaterally choosing a system. Similarly, the DCE tasks presented one-off static decisions, whereas in reality the process is dynamic, with a

For peer review only - http://bmjopen.bmj.com/site/about/guidelines.xhtml

For peer review only

child being reassessed several times and potentially several different AAC systems being provided. These differences are a limiting factor in the external validity of results.

Attributes and levels use a mixture of speech and language therapy terms (e.g. receptive and expressive language) and more technological language (e.g. staged progression). This may have made it difficult for respondents from any one speciality to interpret all of them. However, this issue is not limited to the current study, but reflects an ongoing struggle in AAC to establish a common language, given its interdisciplinary nature. In addition, respondents may have been unfamiliar with the generic term ideographic symbols, since only a single commercial set of ideographic symbols is in popular use (Minspeak, © Semantic Compaction Systems, Inc.).

Compared to the real children AAC professionals encounter, vignettes were simple, and lacking in details that would normally be available. A single vignette also represents potentially very different children. For example, the needs of a child who has plateaued in skills and abilities at age five will be very different to a child who has plateaued at age 15. However, this is an inherent limitation of the DCE methodology, and vignettes with a greater number of attributes and levels would have made decisions overly burdensome. In addition, significant interactions between AAC systems and child attributes implies the vignettes were meaningful enough that respondents changed their preferences in response to them, often dramatically.

For a given child vignette, it is only possible to determine relative preferences for system attributes, rather than absolute preferences. Thus it is not possible to tell how suitable a given system is for a given vignette, which is important as some vignettes presented a challenging profile for which it may be hard to find a suitable AAC system. It does not follow from this analysis that there is "something for everyone".

CONCLUSION

This is the first study to use DCE methodology to measure the preferences of AAC professionals when choosing AAC systems for children. It has shown that AAC professionals' decision making can be strongly influenced by the characteristic of the child they are providing a system for. In particular, whether a child is

For peer review only - http://bmjopen.bmj.com/site/about/guidelines.xhtml

motivated to communicate using AAC or is predicted to progress in skills and abilities has a large impact on their priorities.

Acknowledgements: Thank you to Muireann McCleary and the Speech and Language Therapy team at the Central Remedial Clinic who piloted and gave feedback on the survey, and to participants who responded to the survey.

Funding: This independent research was funded by the National Institute for Health Research, UK (Health Services & Delivery Research Project: 14/70/153 - Identifying appropriate symbol communication aids for children who are non-speaking: enhancing clinical decision-making). The views expressed in this article are those of the authors and not necessarily those of the NHS, the National Institute for Health Research or the Department of Health.

Stephane Hess acknowledges additional support by the European Research Council through the consolidator grant 615596-DECISIONS.

Competing interests: The authors have no competing interests to declare.

Author statement: All authors conceived the study and defined the study aims. EW, DM, YL, NR, SJ, JG, SM and LM developed attributes and levels. EW, DM and SH constructed the survey statistical design. EW and DM collected data. EW conducted statistical analysis. EW, YL, NR, SJ, JG, SM, LM and JM interpreted findings. EW wrote the manuscript first draft. All authors contributed to and approved the final manuscript.

Ethical approval: Ethical approval was received for the study from an NHS Research Ethics Committee (REC reference 6/NW/0165) and informed consent was obtained from participants at the start of the survey.

Data availability: Survey data is not publically available as respondent consent was not obtained for this. However, it is available on request to the corresponding author or to Leeds Institute of Health Sciences if a formal data sharing agreement is entered into.

For peer review only - http://bmjopen.bmj.com/site/about/guidelines.xhtml

REFERENCES

1. Murray J, Goldbart J. Augmentative and alternative communication: a review of current issues. Paediatrics and child health 2009;**19**(10):464-68.

2. Schlosser RW, Wendt O. Effects of augmentative and alternative communication intervention on speech production in children with autism: A systematic review. American Journal of Speech-Language Pathology 2008;**17**(3):212-30.

3. Millar DC, Light JC, Schlosser RW. The impact of augmentative and alternative communication intervention on the speech production of individuals with developmental disabilities: A research review. Journal of Speech, Language, and Hearing Research 2006;**49**(2):248-64.

4. Lund SK, Light J. Long-term outcomes for individuals who use augmentative and alternative communication: Part I–What is a "good" outcome? Augmentative and Alternative Communication 2006;**22**(4):284-99.

5. Hajjar DJ, McCarthy JW, Benigno JP, et al. "You Get More Than You Give": Experiences of Community Partners in Facilitating Active Recreation with Individuals who have Complex Communication Needs. Augmentative and Alternative Communication 2016;**32**(2):131-42.

6. Ryan SE, Shepherd T, Renzoni AM, et al. Towards Advancing Knowledge Translation of AAC Outcomes Research for Children and Youth with Complex Communication Needs. Augmentative and Alternative Communication 2015;**31**(2):137-47.

7. Dada S, Alant E. The effect of aided language stimulation on vocabulary acquisition in children with little or no functional speech. American Journal of Speech-Language Pathology 2009;**18**(1):50-64.

8. Binger C, Ball L, Dietz A, et al. Personnel roles in the AAC assessment process. Augmentative and Alternative Communication 2012;**28**(4):278-88.

9. Lindsay S. Perceptions of health care workers prescribing augmentative and alternative communication devices to children. Disability and Rehabilitation: Assistive Technology 2010;**5**(3):209-22.

10. Guidance for commissioning AAC services and equipment: NHS England, 2016.

For peer review only - http://bmjopen.bmj.com/site/about/guidelines.xhtml

11. Gross J. Augmentative and alternative communication: a report on provision for children and young people in England: Office of the Communication Champion, 2010.

12. Enderby P, Judge S, Creer S, et al. Examining the Need for and Provision of AAC Methods in the UK. Advances in Clinical Neuroscience & Rehabilitation 2013;**13**:20-23.

13. Judge S, Enderby P, Creer S, et al. Provision of powered communication aids in the United Kingdom. Augmentative and Alternative Communication 2017;**33**(3):181-87.

14. Johnson JM, Inglebret E, Jones C, et al. Perspectives of speech language pathologists regarding success versus abandonment of AAC. Augmentative and Alternative Communication 2006;**22**(2):85-99.

15. Moorcroft A, Scarinci N, Meyer C. A systematic review of the barriers and facilitators to the provision and use of low-tech and unaided AAC systems for people with complex communication needs and their families. Disability and Rehabilitation: Assistive Technology 2018:1-22.

16. Reddington J. The Domesday dataset: Linked open data in disability studies. Journal of Intellectual Disabilities 2013;**17**(2):107-21.

17. Munton T. Augmentative and Alternative Communication (AAC) support in Scotland: A review of the research literature and cost benefit analyses: NHS Education for Scotland, 2013.

18. Thistle JJ, Wilkinson KM. Building evidence-based practice in AAC display design for young children: Current practices and future directions. Augmentative and Alternative Communication 2015;**31**(2):124-36.

19. Resource manual for commissioning and planning services for SLCN: Royal College of Speech and Language Therapists, 2009.

20. Baxter S, Enderby P, Evans P, et al. Barriers and facilitators to the use of high-technology augmentative and alternative communication devices: a systematic review and qualitative synthesis. International Journal of Language & Communication Disorders 2012;**47**(2):115-29.

21. Lund SK, Quach W, Weissling K, et al. Assessment with children who need augmentative and alternative communication (AAC): Clinical decisions of AAC specialists. Language, speech, and hearing services in schools 2017;**48**(1):56-68.

For peer review only - http://bmjopen.bmj.com/site/about/guidelines.xhtml

22. Geytenbeek JJ, Vermeulen RJ, Becher JG, et al. Comprehension of spoken language in non-speaking children with severe cerebral palsy: an explorative study on associations with motor type and disabilities. Developmental Medicine & Child Neurology 2015;**57**(3):294-300.

23. Webb EJ, Meads D, Lynch Y, et al. What's important in AAC decision making for children? Evidence from a best-worst scaling survey. Augmentative and Alternative Communication Forthcoming.

24. CMC. CMC choice modelling code for R: Choice Modelling Centre, University of Leeds, 2017.

25. Holm S. A simple sequentially rejective multiple test procedure. Scandinavian journal of statistics 1979;**6**(2):65-70.

26. Hauber AB, González JM, Groothuis-Oudshoorn CG, et al. Statistical methods for the analysis of discrete choice experiments: a report of the ISPOR Conjoint Analysis Good Research Practices Task Force. Value in health 2016;**19**(4):300-15.

27. SEND Code of Practice 0–25 Years London: Department for Education, Department of Health, 2015.

For peer review only

Table 1: Child attributes and levels including brief descriptions

Child attributes and levels	Description
**Receptive and expressive language (1)**	Child's ability to understand communication from and communicate with others without AAC
*Delayed	Both receptive and expressive abilities below expectation given child's age
Receptive language exceeding expressive language	Ability to understand communication from others greater than ability to communicate with others
**Communication ability with AAC (3)**	How well a child can communicate when using AAC
*No previous AAC experience	Has never communicated using AAC before
Abe to use AAC for a few communicative functions	Can use AAC for some basic functions, e.g. simple requests
Able to use AAC for a range of communicative functions	Can use AAC for more complex tasks, e.g. constructing sentences
**Child's determination and persistence (4)**	Attitude of child towards communication and using AAC
*Does not appear motivated to communicate through any methods and means	Child is not inclined to develop communication skills
Motivated to communicate through symbol communication systems	Child has demonstrated motivation and willingness to use AAC
Only motivated to communicate through methods other than symbol communication	Child may be motivated to communicate, but is not inclined to use AAC
**Predicted future skills and abilities (6)**	Professional assessment of how child's communication abilities will develop
*Regression	Abilities projected to become worse in future, e.g. due to a degenerative condition such as Rett syndrome
Plateau	Abilities will not change significantly in future, e.g. a child aged 16-17
Progression	Communication abilities will develop in future

Note: * indicates baseline level; numbers in parentheses indicate attributes' rank in relative importance from Webb et al. [23].

For peer review only

For peer review only - http://bmjopen.bmj.com/site/about/guidelines.xhtml

Table 2: AAC System attributes and levels, including brief descriptions

AAC System attributes and levels	Description
**Vocabulary sets (1)**	Words and/or symbols pre-provided on system, usually as part of a software package
*No vocabulary set	AAC practitioners/child's support network provides all vocabulary content
Fixed vocabulary set	A single fixed set of vocabulary which may be customised
Vocabulary set with staged progression	A series of vocabulary sets with pre-determined progression through them that simulate language development. E.g. an initial set including just basic words, with subsequent sets introducing more grammatical structure. May be customised.
**Consistency of layout (2)**	How consistent positions of words/symbols are in system interface, and how consistent navigation to find different symbols is
*Consistency of some aspects of layout	Words/symbols in multiple categories appear in different positions across categories, but always in the same place in a given category
Consistency of all aspects of layout	All/nearly all words/symbols always appear in same position in interface
Idiosyncratic layout	Layout that has been personalised for an individual child
**Type of vocabulary organisation (5)**	How words/symbols are organised within the system
*Visual scene	Interface shows photos, most likely of scenes familiar to the child, with areas of it highlighted to represent words
Taxonomic	Words/symbols organised according to subject, analogous to non-fiction books in a library
Semantic-syntactic	Words/symbols organised according to sentence structure, e.g. verbs, nouns, adjectives
Pragmatic	Words/symbols organised around function in language rather than grammar, e.g. request, mood
**Size of vocabulary (7)**	How many words/symbols system can output
*Up to 50 vocabulary items	Implies only simple communication functions possible
50-1000 vocabulary items	Implies combining words/symbols to create grammatical structures
More than 1000 vocabulary items	Does not imply more complex communication than 50-1000 items, but means a greater load on child's memory
**Graphic representation (12)**	Type of symbols used by system
*Photos	Photographs, possibly of items or environments personal to the child
Pictographic symbol set	Non-photorealist pictures with specific meanings attached. May be accompanied by text
Ideographic symbol system (with rules or encoding)	Stylised symbols combined with fixed rules and grammar analogous to Chinese/Japanese characters, e.g. Minspeak
Text	Text unaccompanied by other symbols

Note: * indicates baseline level; numbers in parentheses indicate attributes' rank in relative importance from prior BWS study (reported in Webb et al. [23]).

For peer review only

Table 3: Demographics and professional experience of participants

		mean	s.e
Age (years)		40.8	11
Experience (years)		11.4	9.2
% of role relating to AAC		53.7	34.3
		N	%
Gender	Female	155	90.1
	Male	10	5.81
	Prefer not to say	7	4.07
Ethnicity	White - English/Welsh/Scottish/Northern Irish/British	149	86.6
	White – other	12	6.98
	Other	6	3.49
	White – Irish	5	2.91
Professional background	Speech and language therapist	125	72.7
	Occupational therapist	16	9.3
	Teacher	14	8.14
	Other	12	6.98
	Assistive technology specialist	5	2.91
	Clinical scientist	5	2.91
Age groups worked with	Primary school age	99	57.6
	Secondary school age	94	54.7
	Pre-school age	85	49.4
	All age groups	56	32.6
	Higher education	30	17.4
	Further education	21	12.2
	Other	12	6.98
	Adults	10	5.81
Among most common three diagnoses seen in practice	Physical disability (e.g. neuromuscular, cerebral palsy etc.)	140	81.4
	Intellectual Disability/Developmental Delay	118	68.6
	Autism spectrum disorder	113	65.7
	Syndromes	61	35.5
	Neurological	45	26.2
	Specific Speech/Language Impairment	22	12.8
	Dyspraxia	14	8.14

Note. For some questions, participants could select more than one response, thus some percentages do not sum to 100%

For peer review only

Table 4: Parameter means and standard deviations for final mixed logit model. * indicates significance at the 5% level corrected using Holm's sequential Bonferroni [25], s.e. = standard error

AAC system attribute		Child attribute	Parameter mean	s.e.	σ	s.e.
Vocabulary sets (baseline none)	Fixed	Constant	0.283*	0.0966	0.131	0.258
	Staged progression	Constant	0.364*	0.141	0.941*	0.206
		Predicted to progress	1.36*	0.221	-1.09*	0.343
Consistency of layout (baseline some aspects)	Consistency of all aspects	Constant	0.892*	0.121	0.15	0.126
	Idiosyncratic layout	Constant	1.46*	0.14	0.757*	0.134
Type of vocabulary organisation (baseline visual scene)	Taxonomic	Constant	0.0629	0.165	0.383	0.257
		Motivated to communicate through symbol communication systems	0.707*	0.206	-0.563	0.295
	Semantic-syntactic	Constant	-0.178	0.166	0.549	0.234
		Motivated to communicate through symbol communication systems	0.826*	0.197	-0.112	0.296
	Pragmatic	Constant	0.443*	0.123	0.723*	0.152
Size of vocabulary (baseline 50 items)	50-1000 items	Constant	0.131	0.143	0.43	0.166
		Motivated to communicate through symbol communication systems	1.01*	0.232	-0.731	0.329
	More than 1000 items	Constant	-0.929*	0.213	1.02*	0.33
		Receptive language exceeding expressive language	0.692*	0.186	0.489	0.367
		Able to use AAC for a range of communicative functions	1.14*	0.319	-0.419	0.762
		Motivated to communicate through symbol communication systems	1.31*	0.272	-0.751	0.556
		Predicted to progress	0.902*	0.233	0.981	0.657
Graphic representation (baseline photos)	Pictographic symbol set	Constant	-0.41	0.183	0.0722	0.248
		Motivated to communicate through symbol communication systems	1.36*	0.24	-0.363	0.428
		Predicted to progress	-0.814*	0.217	1.12	0.385
	Ideographic symbol system	Constant	-1.25*	0.207	0.823*	0.216
		Motivated to communicate through symbol communication systems	1.67*	0.268	0.069	0.297
	Text	Constant	-0.709*	0.159	0.615*	0.204
		Motivated to communicate through symbol communication systems	1.39*	0.231	-1.12*	0.282

Note. σ indicates standard deviation. Parameter variance for level l of AAC system attribute a when choosing for child c is given by $\sigma^2_{alc} = (\sigma_{al0} + \sigma_{al} Z_c)^2$.

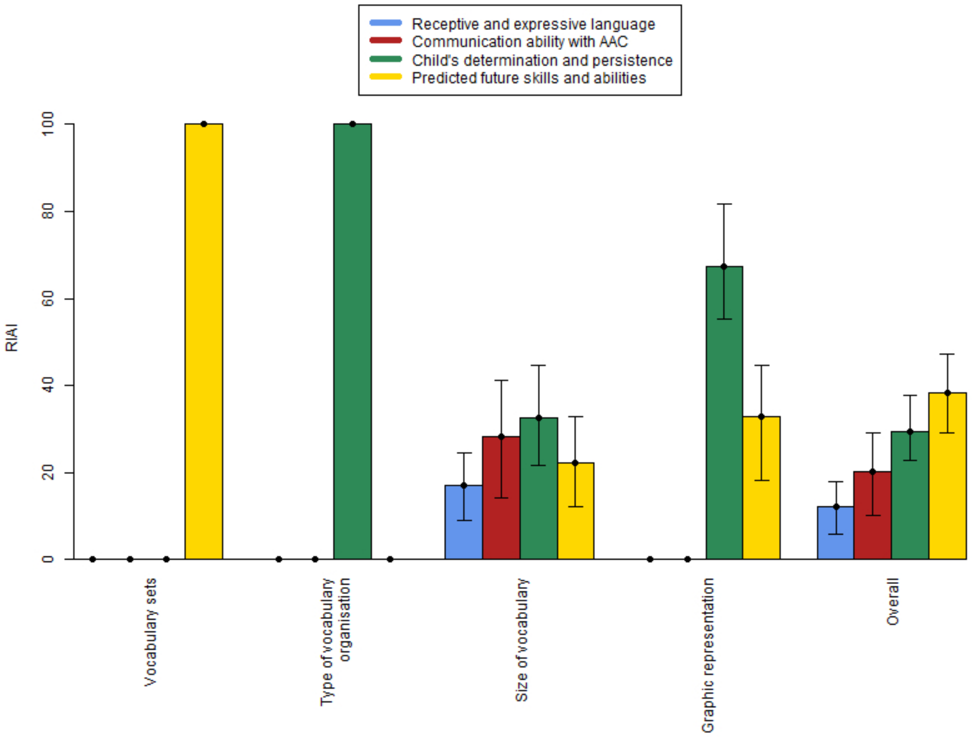

Relative interaction attribute importance for each AAC system attribute and averaged over all attributes. Note that consistency of layout is omitted as there are no interactions with child attributes. Error bars show 95% confidence intervals.

282x211mm (72 x 72 DPI)

Appendix A – Example survey

Note: the AAC system levels and child vignettes shown here are for illustrative purposes and do not represent the statistical design used in the full survey.

Instructions

Thank you for taking part in this survey.

It aims to identify what factors clinicians think are important when making decisions about aided AAC systems for children with communication difficulties.

You will be asked a series of questions. Each one has the same format. A brief description of a child will be given, along with three possible choices of aided AAC systems.

The three AAC systems are described in terms of five characteristics (the systems are identical apart from changes to these five characteristics):-

1. **Vocabulary sets**: Pre-determined vocabulary or language package provided, which can be:-
   - No commercially provided sets
   - Commercially provided sets without language progression
   - Commercially provided sets with language progression
2. **Size of vocabulary**: The size of the output vocabulary available within the aided AAC system, which can be:-
   - Up to 50 vocabulary items
   - 50-1000 vocabulary items
   - More than 1000 vocabulary items
3. **Type of vocabulary organisation**: Primary format used to organise the vocabulary within the aided AAC system, which can be:-
   - Visual scene display
   - Semantic organisation
   - Semantic syntactic organisation
   - Pragmatic organisation

For peer review only - http://bmjopen.bmj.com/site/about/guidelines.xhtml

4. **Graphic Representation**: Primary type of graphic symbol used, which can be:-
   - Photo symbols (i.e. a photo symbol set without rules or encoding)
   - Pictographic symbols (i.e. a graphic symbol set without rules or encoding)
   - Ideographic symbols (i.e. a symbol system with rules or encoding)
   - Graphic symbols with text (i.e. a system with either pictographic or ideographic symbols that incorporates an alphabet for generating text)
5. **Consistency of layout**: Consistency of layout of symbols on pages, including when navigating through pages to select desired output, which can be:-
   - Inconsistent layout
   - Somewhat consistent layout
   - Highly consistent layout

Imagine you had to choose between only these three systems. You should indicate which you would prescribe for the child described. If your preferred option is not available, pick the system from the three options that you think best matches the child's needs. There are no right or wrong answers. It is acknowledged that this may feel uncomfortable for you.

In the survey, there are three different children described. You will be asked four questions about each child (12 questions in total).

In acknowledgement of choices being uncomfortable, after each choice, you will be asked to indicate how well you think that system matches the child's needs. (1 = very unsuitable, 7 = very suitable).

This survey is part of independent research funded by the National Institute for Health Research (NIHR), Health Service and Delivery Research (HS&DR) Programme 14/70/153. The views expressed are those of the authors and do not necessarily reflect those of the NHS, the NIHR, NETSCC, the HS&DR programme or the Department of Health.

Consent

For peer review only

Your participation in this survey is voluntary. All information is collected anonymously and held in confidence. We hope you complete the survey but you are free to stop responding at any point resulting in your answers will be removed.

q I have read and understood the above and consent to taking part.

I confirm my work involves assessing children for aided AAC systems and I contribute to the decision making in relation to the language and vocabulary organisation within aided AAC systems.

q Yes

q No

If yes go to DCE questions.

If no go to a page with the following:-

Thank you for your interest in this survey. At present we are only recruiting participants who contribute to decision making in relation to the language and vocabulary organisation within aided AAC for children. Over the coming 12 months we will be recruiting people with a wider range of AAC experience to test decision making resources we are developing. If you are interested in this aspect of the project or would like to be notified when the free resources are available, there will be an opportunity at the end to submit your email address.

We would still like to ask you a few questions about your experience with AAC to check the representativeness of participants.

Then go directly to demographics questionnaire.

For peer review only

Question 1

Child A has delayed expressive and receptive language and is able to use aided AAC for a few communicative functions. Child A is motivated to communicate through symbol communication systems. Child A is predicted to regress in skills and abilities (regression).

	**System 1**	**System 2**	**System 3**
**Size of vocabulary** The size of the output vocabulary available within the aided AAC system.	50-1000 vocabulary items	50-1000 vocabulary items	50-1000 vocabulary items
**Type of vocabulary organisation** Primary format used to organise the vocabulary within the aided AAC system	Pragmatic	Visual Scene	Visual Scene
**Vocabulary sets** Pre-determined vocabulary or language package provided	No vocabulary set	Vocabulary sets with staged progression	Fixed vocabulary set
**Graphic representation** Primary type of graphic symbol used	Ideographic symbol system (with rules or encoding)	Photos	Ideographic symbol system (with rules or encoding)
**Type of vocabulary organisation** Primary format used to organise the vocabulary within the aided AAC system	Idiosyncratic layout	Idiosyncratic layout	Consistency of some aspects of layout

For this child I would choose:

- q System 1
- q System 2
- q System 3

On a scale from 1 to 7, how good a match is your chosen device for this child? (1=very unsuitable, 7=very suitable).

q	q	q	q	q	q	q
1	2	3	4	5	6	7

For peer review only

Question 2

Child A has delayed expressive and receptive language and is able to use aided AAC for a few communicative functions. Child A is motivated to communicate through symbol communication systems. Child A is predicted to regress in skills and abilities (regression).

	System 1	System 2	System 3
**Size of vocabulary**<br>The size of the output vocabulary available within the aided AAC system.	50-1000 vocabulary items	50-1000 vocabulary items	50-1000 vocabulary items
**Type of vocabulary organisation**<br>Primary format used to organise the vocabulary within the aided AAC system	Taxonomic	Visual Scene	Pragmatic
**Vocabulary sets**<br>Pre-determined vocabulary or language package provided	Vocabulary sets with staged progression	No vocabulary set	Fixed vocabulary set
**Graphic representation**<br>Primary type of graphic symbol used	Text	Pictographic symbol set	Photos
**Type of vocabulary organisation**<br>Primary format used to organise the vocabulary within the aided AAC system	Consistency of some aspects of layout	Idiosyncratic layout	Consistency of some aspects of layout

For this child I would choose:

- q System 1
- q System 2
- q System 3

On a scale from 1 to 7, how good a match is your chosen device for this child? (1=very unsuitable, 7=very suitable).

q	q	q	q	q	q	q
1	2	3	4	5	6	7

For peer review only

Question 3

Child A has delayed expressive and receptive language and is able to use aided AAC for a few communicative functions. Child A is motivated to communicate through symbol communication systems. Child A is predicted to regress in skills and abilities (regression).

	System 1	System 2	System 3
**Size of vocabulary**<br>The size of the output vocabulary available within the aided AAC system.	50-1000 vocabulary items	50-1000 vocabulary items	Up to 50 vocabulary items
**Type of vocabulary organisation**<br>Primary format used to organise the vocabulary within the aided AAC system	Semantic-Syntactic	Semantic-Syntactic	Visual Scene
**Vocabulary sets**<br>Pre-determined vocabulary or language package provided	Vocabulary sets with staged progression	No vocabulary set	Fixed vocabulary set
**Graphic representation**<br>Primary type of graphic symbol used	Text	Photos	Pictographic symbol set
**Type of vocabulary organisation**<br>Primary format used to organise the vocabulary within the aided AAC system	Consistency of all aspects of layout	Idiosyncratic layout	Consistency of some aspects of layout

For this child I would choose:

- q System 1
- q System 2
- q System 3

On a scale from 1 to 7, how good a match is your chosen device for this child? (1=very unsuitable, 7=very suitable).

q	q	q	q	q	q	q
1	2	3	4	5	6	7

For peer review only

Question 4

Child A has delayed expressive and receptive language and is able to use aided AAC for a few communicative functions. Child A is motivated to communicate through symbol communication systems. Child A is predicted to regress in skills and abilities (regression).

	System 1	System 2	System 3
**Size of vocabulary**<br>The size of the output vocabulary available within the aided AAC system.	50-1000 vocabulary items	50-1000 vocabulary items	50-1000 vocabulary items
**Type of vocabulary organisation**<br>Primary format used to organise the vocabulary within the aided AAC system	Visual Scene	Taxonomic	Pragmatic
**Vocabulary sets**<br>Pre-determined vocabulary or language package provided	Vocabulary sets with staged progression	Fixed vocabulary set	Fixed vocabulary set
**Graphic representation**<br>Primary type of graphic symbol used	Ideographic symbol system (with rules or encoding)	Text	Photos
**Type of vocabulary organisation**<br>Primary format used to organise the vocabulary within the aided AAC system	Consistency of some aspects of layout	Consistency of all aspects of layout	Idiosyncratic layout

For this child I would choose:

- q System 1
- q System 2
- q System 3

On a scale from 1 to 7, how good a match is your chosen device for this child? (1=very unsuitable, 7=very suitable).

q	q	q	q	q	q	q
1	2	3	4	5	6	7

For peer review only

Question 5

Child B has receptive language exceeding expressive language and no previous AAC experience. Child B is only motivated to communicate through methods other than symbol communication systems. Child B is predicted to maintain current skills and abilities (plateau).

	System 1	System 2	System 3
**Size of vocabulary**<br>The size of the output vocabulary available within the aided AAC system.	Up to 50 vocabulary items	50-1000 vocabulary items	More than 1000 vocabulary items
**Type of vocabulary organisation**<br>Primary format used to organise the vocabulary within the aided AAC system	Taxonomic	Visual Scene	Pragmatic
**Vocabulary sets**<br>Pre-determined vocabulary or language package provided	No vocabulary set	Fixed vocabulary set	Vocabulary sets with staged progression
**Graphic representation**<br>Primary type of graphic symbol used	Photos	Text	Ideographic symbol system (with rules or encoding)
**Type of vocabulary organisation**<br>Primary format used to organise the vocabulary within the aided AAC system	Consistency of all aspects of layout	Idiosyncratic layout	Consistency of some aspects of layout

For peer review only

For this child I would choose:

- q System 1
- q System 2
- q System 3

On a scale from 1 to 7, how good a match is your chosen device for this child? (1=very unsuitable, 7=very suitable).

q	q	q	q	q	q	q
1	2	3	4	5	6	7

Question 6

Child B has receptive language exceeding expressive language and no previous AAC experience. Child B is only motivated to communicate through methods other than symbol communication systems. Child B is predicted to maintain current skills and abilities (plateau).

	**System 1**	**System 2**	**System 3**
**Size of vocabulary**<br>The size of the output vocabulary available within the aided AAC system.	More than 1000 vocabulary items	Up to 50 vocabulary items	50-1000 vocabulary items
**Type of vocabulary organisation**<br>Primary format used to organise the vocabulary within the aided AAC system	Semantic-Syntactic	Semantic-Syntactic	Taxonomic
**Vocabulary sets**<br>Pre-determined vocabulary or language package provided	Vocabulary sets with staged progression	Fixed vocabulary set	No vocabulary set
**Graphic representation**<br>Primary type of graphic symbol used	Ideographic symbol system (with rules or encoding)	Photos	Ideographic symbol system (with rules or encoding)
**Type of vocabulary organisation**<br>Primary format used to organise the vocabulary within the aided AAC system	Idiosyncratic layout	Consistency of some aspects of layout	Consistency of some aspects of layout

For this child I would choose:

- q System 1
- q System 2
- q System 3

On a scale from 1 to 7, how good a match is your chosen device for this child? (1=very unsuitable, 7=very suitable).

q	q	q	q	q	q	q
1	2	3	4	5	6	7

For peer review only

Question 7

Child B has receptive language exceeding expressive language and no previous AAC experience. Child B is only motivated to communicate through methods other than symbol communication systems. Child B is predicted to maintain current skills and abilities (plateau).

	System 1	System 2	System 3
**Size of vocabulary**<br>The size of the output vocabulary available within the aided AAC system.	Up to 50 vocabulary items	More than 1000 vocabulary items	50-1000 vocabulary items
**Type of vocabulary organisation**<br>Primary format used to organise the vocabulary within the aided AAC system	Taxonomic	Pragmatic	Taxonomic
**Vocabulary sets**<br>Pre-determined vocabulary or language package provided	Fixed vocabulary set	Vocabulary sets with staged progression	No vocabulary set
**Graphic representation**<br>Primary type of graphic symbol used	Photos	Ideographic symbol system (with rules or encoding)	Text
**Type of vocabulary organisation**<br>Primary format used to organise the vocabulary within the aided AAC system	Consistency of all aspects of layout	Consistency of some aspects of layout	Idiosyncratic layout

For this child I would choose:

- q System 1
- q System 2
- q System 3

On a scale from 1 to 7, how good a match is your chosen device for this child? (1=very unsuitable, 7=very suitable).

q	q	q	q	q	q	q
1	2	3	4	5	6	7

For peer review only

Question 8

Child B has receptive language exceeding expressive language and no previous AAC experience. Child B is only motivated to communicate through methods other than symbol communication systems. Child B is predicted to maintain current skills and abilities (plateau).

	System 1	System 2	System 3
**Size of vocabulary**<br>The size of the output vocabulary available within the aided AAC system.	Up to 50 vocabulary items	More than 1000 vocabulary items	50-1000 vocabulary items
**Type of vocabulary organisation**<br>Primary format used to organise the vocabulary within the aided AAC system	Pragmatic	Semantic-Syntactic	Visual Scene
**Vocabulary sets**<br>Pre-determined vocabulary or language package provided	Fixed vocabulary set	Fixed vocabulary set	Vocabulary sets with staged progression
**Graphic representation**<br>Primary type of graphic symbol used	Ideographic symbol system (with rules or encoding)	Ideographic symbol system (with rules or encoding)	Photos
**Type of vocabulary organisation**<br>Primary format used to organise the vocabulary within the aided AAC system	Consistency of some aspects of layout	Consistency of all aspects of layout	Idiosyncratic layout

For this child I would choose:

- q System 1
- q System 2
- q System 3

On a scale from 1 to 7, how good a match is your chosen device for this child? (1=very unsuitable, 7=very suitable).

q	q	q	q	q	q	q
1	2	3	4	5	6	7

For peer review only

Question 9

Child C has delayed expressive and receptive language and no previous AAC experience. Child C is only motivated to communicate through methods other than symbol communication systems. Child C is predicted to maintain current skills and abilities (plateau).

	System 1	System 2	System 3
**Size of vocabulary** The size of the output vocabulary available within the aided AAC system.	50-1000 vocabulary items	More than 1000 vocabulary items	Up to 50 vocabulary items
**Type of vocabulary organisation** Primary format used to organise the vocabulary within the aided AAC system	Visual Scene	Pragmatic	Semantic-Syntactic
**Vocabulary sets** Pre-determined vocabulary or language package provided	Fixed vocabulary set	Fixed vocabulary set	Fixed vocabulary set
**Graphic representation** Primary type of graphic symbol used	Pictographic symbol set	Pictographic symbol set	Text
**Type of vocabulary organisation** Primary format used to organise the vocabulary within the aided AAC system	Idiosyncratic layout	Consistency of some aspects of layout	Consistency of all aspects of layout

For this child I would choose:
- q System 1
- q System 2
- q System 3

On a scale from 1 to 7, how good a match is your chosen device for this child? (1=very unsuitable, 7=very suitable).

q	q	q	q	q	q	q
1	2	3	4	5	6	7

For peer review only

Question 10

Child C has delayed expressive and receptive language and no previous AAC experience. Child C is only motivated to communicate through methods other than symbol communication systems. Child C is predicted to maintain current skills and abilities (plateau).

	System 1	System 2	System 3
**Size of vocabulary**<br>The size of the output vocabulary available within the aided AAC system.	Up to 50 vocabulary items	More than 1000 vocabulary items	Up to 50 vocabulary items
**Type of vocabulary organisation**<br>Primary format used to organise the vocabulary within the aided AAC system	Taxonomic	Semantic-Syntactic	Taxonomic
**Vocabulary sets**<br>Pre-determined vocabulary or language package provided	No vocabulary set	Vocabulary sets with staged progression	No vocabulary set
**Graphic representation**<br>Primary type of graphic symbol used	Ideographic symbol system (with rules or encoding)	Ideographic symbol system (with rules or encoding)	Photos
**Type of vocabulary organisation**<br>Primary format used to organise the vocabulary within the aided AAC system	Consistency of all aspects of layout	Consistency of some aspects of layout	Idiosyncratic layout

For this child I would choose:

- q System 1
- q System 2
- q System 3

On a scale from 1 to 7, how good a match is your chosen device for this child? (1=very unsuitable, 7=very suitable).

q	q	q	q	q	q	q
1	2	3	4	5	6	7

For peer review only

Question 11

Child C has delayed expressive and receptive language and no previous AAC experience. Child C is only motivated to communicate through methods other than symbol communication systems. Child C is predicted to maintain current skills and abilities (plateau).

	System 1	System 2	System 3
**Size of vocabulary** The size of the output vocabulary available within the aided AAC system.	Up to 50 vocabulary items	50-1000 vocabulary items	50-1000 vocabulary items
**Type of vocabulary organisation** Primary format used to organise the vocabulary within the aided AAC system	Visual Scene	Semantic-Syntactic	Semantic-Syntactic
**Vocabulary sets** Pre-determined vocabulary or language package provided	No vocabulary set	Vocabulary sets with staged progression	No vocabulary set
**Graphic representation** Primary type of graphic symbol used	Photos	Pictographic symbol set	Text
**Type of vocabulary organisation** Primary format used to organise the vocabulary within the aided AAC system	Idiosyncratic layout	Consistency of some aspects of layout	Consistency of all aspects of layout

For this child I would choose:

- q System 1
- q System 2
- q System 3

On a scale from 1 to 7, how good a match is your chosen device for this child? (1=very unsuitable, 7=very suitable).

q	q	q	q	q	q	q
1	2	3	4	5	6	7

For peer review only

Question 12

Child C has delayed expressive and receptive language and no previous AAC experience. Child C is only motivated to communicate through methods other than symbol communication systems. Child C is predicted to maintain current skills and abilities (plateau).

	**System 1**	**System 2**	**System 3**
**Size of vocabulary** The size of the output vocabulary available within the aided AAC system.	Up to 50 vocabulary items	More than 1000 vocabulary items	50-1000 vocabulary items
**Type of vocabulary organisation** Primary format used to organise the vocabulary within the aided AAC system	Semantic-Syntactic	Semantic-Syntactic	Visual Scene
**Vocabulary sets** Pre-determined vocabulary or language package provided	No vocabulary set	Vocabulary sets with staged progression	Fixed vocabulary set
**Graphic representation** Primary type of graphic symbol used	Ideographic symbol system (with rules or encoding)	Text	Photos
**Type of vocabulary organisation** Primary format used to organise the vocabulary within the aided AAC system	Consistency of all aspects of layout	Idiosyncratic layout	Consistency of some aspects of layout

For this child I would choose:

- q System 1
- q System 2
- q System 3

On a scale from 1 to 7, how good a match is your chosen device for this child? (1=very unsuitable, 7=very suitable).

q	q	q	q	q	q	q
1	2	3	4	5	6	7

For peer review only

Questionnaire

In this final part of the survey, we'd like to ask you for some information about yourself and your experience with AAC.

We would like to know about the characteristics of the people who complete this survey to check that we have a representative sample. We would also like to check if people with different professional experiences have different opinions.

All responses will be held anonymously and we have no way of tracing your responses back to you as an individual.

Q. What is your age? _______ years.

Q. What is your gender?

q Male q Female q Other q Prefer not to say

Q. How would you describe your ethnicity?

q White - English/Welsh/Scottish/Northern Irish/British

q White -Irish

q White - Gypsy or Irish Traveller

q White - Any other White background

q Mixed/Multiple ethnic group - White and Black Caribbean

q Mixed/Multiple ethnic group - White and Black African

q Mixed/Multiple ethnic group - White and Asian

q Mixed/Multiple ethnic group - Any other Mixed/Multiple ethnic background

q Asian/Asian British - Indian

q Asian/Asian British - Pakistani

q Asian/Asian British – Bangladeshi

q Asian/Asian British - Chinese

q Asian/Asian British - Any other Asian background

q Black/ African/Caribbean/Black British - African

For peer review only

q Black/ African/Caribbean/Black British - Caribbean

q Black/ African/Caribbean/Black British - Any other Black/African/Caribbean background

q Arab

q Other

Q. For how many years have you worked with AAC? _______ years.

Q. What is your professional background? You may select more than one option if applicable.

q Occupational therapist

q Speech and language therapist

q Assistive technology specialist

q Clinical scientist

q Teacher

q Other

Q. If you selected Other, please specify.

____

Q. How much of your role relates to AAC? _______%.

(e.g. 1 day per week = 20%, 2 days a week = 40%, etc.)

Q. How would you characterise your role? Pick the one that best describes your role.

q I refer on anyone who may benefit from AAC

q I assess and implement AAC. I seek support from within my own team for decisions made

q I assess and implement AAC. I seek support from outside my own team for

q decisions made

q I assess and implement AAC. I act as a support for others in relation to AAC

q decision making

q I assess only. I provide support to others outside my team in relation to

q AAC decision making

q Other

Q. If you selected Other, please specify.

____

Q. Out of the list below, select the three most common diagnoses you encounter in your work.

q Autism Spectrum Disorder

q Physical disability (e.g. neuromuscular, cerebral palsy etc.)

q Dyspraxia

q Intellectual Disability/Developmental Delay

q Neurological

q Specific Speech/Language Impairment

q Syndromes

q Unknown

q Other

Q. If you selected Other, please specify.

____

Q. Who do you provide services for? (Please choose all that apply.)

q All age groups

q Preschool age

q Primary school age

q Secondary school age

q Higher education

q Further education

q Adults

q Other

Q. If you selected Other, please specify.

____

Q. What is the geographical area covered by your service? (Please choose all that apply.

q North West England

q North East England

q Yorkshire and Humber

q West Midlands

q East Midlands

q East of England

For peer review only

- q South West England
- q South East England
- q London
- q Northern Ireland
- q North Wales
- q South Wales
- q Mid-Wales
- q Southern Scotland
- q Central Scotland
- q Northern Scotland
- q Non-UK

End of survey

Thank you for your participation in this survey.

Your responses will contribute to the results of the I-ASC project and support the development of decision making resources for use in AAC assessments.

You can follow the progress of our research project on our website, on Facebook or on Twitter.

For peer review only

Figure A 1: Example discrete choice experiment task

Child B has delayed expressive and receptive language and able to use aided AAC for a few communicative functions. Child B is only motivated to communicate through methods other than symbol communication systems. Child B is predicted to maintain current skills and abilities (plateau).

	System 1	System 2	System 3
Graphic Representation<br>Primary type of graphic symbol used	Ideographic symbols	Photo symbols	Pictographic symbols
Consistency of layout<br>Consistency of layout of symbols on pages, including when navigating through pages to select desired output.	Highly consistent layout	Somewhat consistent layout	Inconsistent layout
Vocabulary sets<br>Pre-determined vocabulary or language package provided	Commercially provided sets without language progression	Commercially provided sets with language progression	Commercially provided sets without language progression
Size of vocabulary<br>The size of the output vocabulary available within the aided AAC system.	More than 1000 vocabulary items	50-1000 vocabulary items	Up to 50 vocabulary items
Type of vocabulary organisation<br>Primary format used to organise the vocabulary within the aided AAC system	Pragmatic organisation	Semantic syntactic organisation	Visual scene display

For this child I would choose: ○ ○ ○

On a scale from 1 to 7, how good a match is your chosen device for this child? (1=very unsuitable, 7=very suitable)

1 = very unsuitable	2	3	4	5	6	7 = very suitable
○	○	○	○	○	○	○

35%

Appendix B – Attributes from best-worst scaling case 1 study

Table B 1: Attributes used in best-worst scaling case 1 survey in Webb et al. [23] and rank in terms of relative importance score

Child attribute	Rank
*Child’s receptive and expressive language abilities	1
Support for AAC from communication partners	2
*Communication ability with aided AAC	3
*Child's determination and persistence	4
Physical abilities for access	5
*Predicted future needs and abilities	6
Level of learning ability	7
Insight into own communicative skills	8
Attention level	9
Access to professional AAC support	10
Speech skills and intelligibility	11
Functional visual skills	12
History of aided AAC use	13
Presence of additional diagnoses	14
Level of fatigue	15
Literacy ability	16
Educational stage	17
Primary diagnosis	18
Mobility	19
**AAC system attributes**	**Rank**
*Vocabulary or language package(s)	1
*Consistency of layout and navigation	2
Ease of customization	3
Durability and reliability	4
*Type of vocabulary organization	5
Number of key presses required to generate symbol or text output	6
*Size of output vocabulary	7
Range of access methods	8
Number of cells per page	9
Portability	10
*Graphic representation	11
Battery life	12
Supplier support	13
Ease of mounting on a range of equipment	14
Cost	15
Additional assistive technology functions	16
Voice	17
Appearance	18

Note. Asterisk indicates attribute included in discrete choice experiment.

For peer review only

Appendix C – Final preferred model selection process

A full model with all interaction terms and two alternative specific constants implies 98 parameters, which is too many to reliably estimate given the amount of data collected and given that many interactions are expected to be of very low magnitude. Thus, a strategy was required to identify a suitable model with fewer parameters.

The first stage was estimating a series of stepwise multinomial logit (MNL) models, beginning with a model with all 98 parameters. The parameter with the highest p-value, excluding the γ_0 constant terms, was eliminated, and a model with 97 parameters was estimated. Then the parameter with the lowest p-value was excluded and a new model run, and so on in an iterative process until only the 12 γ_0 constant terms remained (one for each non-baseline system level).

The Bayesian Information Criterion (BIC) was used to select the preferred MNL model. This model was then re-estimated as a mixed logit (MIXL) model to account for participant heterogeneity. (The process did not begin by estimating a series of stepwise MIXL models due to the difficulty and greatly increased computational resources required to estimate MIXL models with a large number of parameters.) The β \coefficients on system attribute levels were assumed to be drawn from normal distributions with means given by

$$\bar{\beta}_{alc} = \gamma_{al0} + \gamma_{al} z_c$$

and variances given by

$$\sigma_{alc}^2 = (\sigma_{al0} + \sigma_{al} z_c)^2.$$

If p is the number of parameters of the preferred MNL model, then models with between $p - 3$ and $p + 3$ parameters were re-estimated as MIXL models. The BIC for each MIXL model is given in **Error! Reference source not found.**.

The MIXL model minimising the BIC was chosen as the final preferred model.

For peer review only

Table C 1: Bayesian information criteria (BIC) for estimated mixed logit models

Number of parameters	BIC
22	3502.25
23	3487.80
24	3482.30
25	3489.18
26	3493.07
27	3502.28
28	3509.34

For peer review only

Appendix D – Relative interaction attribute importance

Relative information attribute importance (RIAI) measures the amount that preferences for attributes of choice objects are impacted by a given interaction attribute associated with a choice situation relative to other interaction attributes. It may be calculated either with respect to a single choice object attribute or overall with respect to all choice object attributes.

RIAI is calculated with respect to a single choice object attribute by taking the difference between the greatest increase an interaction attribute causes to a choice object attribute's part worth utility and the greatest decrease, expressed as a percentage of the differences for all interaction attributes. Formally, the RIAI for interaction attribute i with respect to choice attribute c is

$$RIAI_{ic} = 100\left(\frac{\gamma_{ic}^{max} - \gamma_{ic}^{min}}{\sum_{j=1}^{N_I}\gamma_{jc}^{max} - \gamma_{jc}^{min}}\right)$$

where γ_{ic}^{max} and γ_{ic}^{min} are respectively the maximum and minimum coefficients for interaction attribute i with respect to choice attribute c and N_I is the number of interaction attributes. The overall RIAI for i is similarly calculated as

$$RIAI_{i} = 100\left(\frac{\gamma_{i}^{max} - \gamma_{i}^{min}}{\sum_{j=1}^{N_I}\gamma_{j}^{max} - \gamma_{j}^{min}}\right)$$

Where now γ_{i}^{max} and γ_{i}^{min} are respectively the maximum and minimum coefficients for interaction attribute i across all choice attributes.

For peer review only

BMJ Open

Finding the best fit: A discrete choice experiment on the decision making of augmentative and alternative communication professionals

Journal:	*BMJ Open*
Manuscript ID	bmjopen-2019-030274.R1
Article Type:	Original research
Date Submitted by the Author:	30-Sep-2019
Complete List of Authors:	Webb, Edward; University of Leeds, Leeds Institute of Health Sciences<br>Lynch, Yvonne; Manchester Metropolitan University, Faculty of Health, Psychology and Social Care<br>Meads, David; University of Leeds, Leeds Institute of Health Sciences<br>Judge, Simon; Barnsley Hospital NHS Foundation Trust, Barnsley Assistive Technology Team<br>Randall, Nicola; Barnsley Hospital NHS Foundation Trust, Barnsley Assistive Technology Team<br>Goldbart, Juliet; Manchester Metropolitan University, Faculty of Health, Psychology and Social Care<br>Meredith, Stuart; Manchester Metropolitan University, Faculty of Health, Psychology and Social Care<br>Moulam, Liz; Manchester Metropolitan University, Faculty of Health, Psychology and Social Care<br>Hess, Stephane; University of Leeds, Choice Modelling Centre and Institute of Transport Studies<br>Murray, Janice; Manchester Metropolitan University, Faculty of Health, Psychology and Social Care
**Primary Subject Heading**:	Communication
Secondary Subject Heading:	Health economics, Health services research
Keywords:	discrete choice experiment, augmentative and alternative communication, clinical decision making

SCHOLARONE™
Manuscripts

Finding the best fit: A discrete choice experiment on the decision making of augmentative and alternative communication professionals

Edward J.D. Webb[i,ii] Yvonne Lynch[iii,iv] David Meads[ii,v] Simon Judge[iii,vi,vii]

Nicola Randall[iii,vi,viii] Juliet Goldbart[iii,ix] Stuart Meredith[iii,x]

Liz Moulam[iii,xi] Stephane Hess[xii,xiii] Janice Murray[iii,xiv]

For peer review only

Keywords: discrete choice experiment; augmentative and alternative communication; clinical decision making

Word count: 4950

[i] Corresponding author; e.j.d.webb@leeds.ac.uk; +44 113 343 2982; Leeds Institute of Health Sciences (LIHS), Level 10 Worsley Building, Clarendon Way, Leeds, LS2 9NL, UK. OrcID: 0000-0001-7918-839X
[ii] Leeds Institute of Health Sciences, University of Leeds
[iii] Faculty of Health, Psychology and Social Care, Manchester Metropolitan University
[iv] lynchyv@tcd.ie
[v] d.meads@leeds.ac.uk
[vi] Barnsley Assistive Technology Team, Barnsley Hospital NHS Foundation Trust
[vii] Simon.judge@nhs.net
[viii] nicola.randall1@nhs.net
[ix] j.goldbart@mmu.ac.uk
[x] meredithsn@hotmail.com
[xi] lizmoulam@aol.com
[xii] Choice Modelling Centre and Institute for Transport Studies, University of Leeds
[xiii] s.hess@leeds.ac.uk
[xiv] j.murray@mmu.ac.uk

For peer review only - http://bmjopen.bmj.com/site/about/guidelines.xhtml

ABSTRACT

Objectives: Many children can benefit from augmentative and alternative communication (AAC) systems for a wide range of medical reasons (e.g. cerebral palsy, autism). However, little is known about professionals' decision-making when recommending symbol based AAC systems for children. This study examines AAC professionals' preferences for attributes of AAC systems and how they interact with child characteristics.

Design: AAC professionals answered a discrete choice experiment (DCE) survey with AAC system and child-related attributes, where participants chose an AAC system for a child vignette.

Setting: The survey was administered online in the UK.

Participants: 155 UK-based AAC professionals were recruited between 20/10/17 and 4/3/18.

Outcomes: The study outcomes were AAC professionals' preferences as quantified using a mixed logit model, with model selection performed using a stepwise procedure and the Bayesian Information Criterion.

Results: Significant differences were observed in preferences for AAC system attributes, and large interactions were seen between child attributes included in the child vignettes, e.g., participants made more ambitious choices for children who were motivated to communicate using AAC, and predicted to progress in skills and abilities. These characteristics were perceived as relatively more important than language ability and previous AAC experience.

Conclusions: AAC professionals make trade-offs between attributes of AAC systems, and these trade-offs change depending on the characteristics of the child for whom the system is being provided.

STRENGTHS AND LIMITATIONS OF THIS STUDY

- This is the first discrete choice experiment, and only the second stated preferences study in the field of augmentative and alternative communication.
- The study used unusual and innovative methodology by (1) using a Best-worst Scaling case 1 study in attribute selection; (2) having AAC system choices be made in the context of a child vignette

For peer review only - http://bmjopen.bmj.com/site/about/guidelines.xhtml

formed from a set of attributes; and (3) introducing a new measure termed relative interaction attribute importance to interpret results.

- Child vignettes were relatively simple, and a single vignette could represent children with very different needs.
- In some ways, the discrete choice experiment task differed from how augmentative and alternative professionals make decisions in practice.

INTRODUCTION

Many people struggle to produce intelligible speech for a wide range of reasons, including cerebral palsy, intellectual/developmental delays and autism spectrum. Even within diagnoses, individuals' communication related needs and abilities are extremely varied. Augmentative and alternative communication (AAC) refers to methods of supporting communication. AAC systems encompass unaided methods including signing, facial expressions, body language, as well as the use of aided systems.[1] This article focuses on aided systems, also known as communication aids, which include high-tech electronic devices, such as those used by Stephen Hawking or Britain's Got Talent winner Lee Ridley, as well as low-tech systems, such as boards and communication books.

AAC can improve the lives of people with communication disabilities.[2-4] Appropriate AAC is especially important for the estimated 1 in 200 children in the UK[5-7] who require these kind of supports. Not only are their language and communication abilities still developing and their needs evolving[8-10], the systems used in childhood can potentially have impacts lasting a whole lifetime.[4]

Major advances in the AAC landscape have occurred in recent years.[11 12] These include technological innovation, for example iPads and eye-tracking, though low-tech systems may still offer the best solution in many cases.[13 14] Another development within services is a greater expectation of participation in all aspects of life for people who use AAC,[11 15-17] coupled with advocacy for the right to communicate.[14] New possibilities for AAC have been created by new communication methods such as text messaging[18], email[19] and social media.[20 21]

For peer review only - http://bmjopen.bmj.com/site/about/guidelines.xhtml

Despite the benefits AAC can offer, high rates of abandonment (30-50%) of AAC systems by children have been observed[22 23], with causes of abandonment not well understood. AAC systems can be costly (up to £10,000) and require a large amount of professional support.[24] However, when recommended appropriately and well implemented, AAC systems have been suggested to be a cost-effective use of the UK's National Health Service (NHS) resources.[25]

The process through which children receive AAC systems varies, both across and within countries.[26-28] In the UK, the context for this study, children's needs and abilities are commonly assessed by a team of AAC professionals, which may include speech and language therapists, occupational therapists and/or specialist teachers.[29 30] Final recommendations and decision-making about AAC systems are made with variable input from the child and family.

Choosing an AAC system requires consideration of many features. For example, what type of graphic symbols (e.g. photos, stylized pictures, words) to use, how many symbols are available, how they are organized, and how they are accessed.[10 31 32] The large degree of heterogeneity in the population of people who benefit from AAC, and in the systems available, means the assessment and subsequent matching of individual and system is a complex task and unique to each person.[26 28 33]

There is currently a lack of documented evidence for assessment and decision-making processes,[33-35] and what does exist is largely individual case studies.[3 25 36] AAC professionals must often make difficult and complex decisions in a complicated, heterogeneous and rapidly evolving environment, balancing the needs of an individual child, and available resources.[30 37] They must also take account of the cultural and contextual influences shaping each assessment.[13 38] While there are studies which highlight some important factors in decision-making,[6 33 39] available guidelines tend to focus on the organisational structure of services, rather than decision-making as such.[29 34 40]

This study addresses the knowledge gaps by providing quantitative evidence about AAC professionals' decision-making using a survey method termed a discrete choice experiment (DCE). DCEs are commonly used in healthcare,[41-43] and can quantify the preferences of patients, health professionals and the public for

For peer review only - http://bmjopen.bmj.com/site/about/guidelines.xhtml

treatments, service delivery methods, policies, or other things. In this case, the goal was measuring the preferences of AAC professionals when choosing AAC systems.

This study was part of a wider project entitled *Identifying Appropriate Symbol Communication aids for children who are non-speaking: enhancing clinical decision making* (I-ASC), which examined provision of AAC systems for children in the UK. I-ASC had several components, using different research methods[30 37 44 45] to generate a body of evidence on current practice and recommendations for best practice. This has resulted in resources to aid AAC decision-making available here: https://iasc.mmu.ac.uk.

Although there is a lack of robust evidence surrounding the decision-making process, some factors in successful adoption of AAC have been identified. An AAC system is more likely to be adopted by a motivated child[22] with good support from the child's network.[27 33 44] The AAC system must also meet a child's individual needs and circumstances, which will be unique to every child.[14 22 46]

A previous study from the current research project investigated the AAC decision-making process using a Best-worst Scaling (BWS) case 1 survey.[45] This method was chosen as it could quantify which of several child and AAC system related factors (37 in total) AAC professionals considered most and least important in decision-making.

The current study sought to complement the previous work by examining fewer factors in more detail using a DCE.[43] It aimed to quantify the clinical judgements and trade-offs AAC professionals make between different attributes of AAC systems, and how those trade-offs change depending on children's characteristics, things not possible using BWS case 1. This is the first DCE carried out in AAC, and there were challenges associated with performing a DCE with a target population of AAC professionals (for details see discussion). Thus, an additional goal was to establish the feasibility of using DCEs as a research tool in AAC.

METHODS

Survey development

For peer review only - http://bmjopen.bmj.com/site/about/guidelines.xhtml

A DCE on AAC professionals' decision making

No stated preference work existed in AAC prior to this project, and there were a large number of potential attributes with little evidence as to which to include in a DCE. A BWS case 1 study was hence performed initially and the results used to guide attribute selection for the DCE. In line with good practice and to ensure attributes were meaningful and relevant, qualitative methods were used to generate attributes.[47 48] Attributes for the BWS study were generated through focus groups and interviews with AAC professionals, people who use AAC, their families, and other stakeholders; systematic literature reviews; and input from an expert panel. For more details see section 2 of Webb et al. [45]

The BWS study produced relative importance scores for 19 child and 18 system attributes given in Appendix A. DCE attributes were selected from these during consensus discussions between authors with expertise in AAC, speech and language therapy, and health economics. The selection criteria were that attributes should: (1) form coherent and realistic descriptions of children and systems; (2) address the research aims of the wider research project, e.g., a focus on symbol communication systems; (3) include mainly attributes with high relative importance scores in the BWS study; and (4) be small in number so choice tasks would not over-burden respondents. Consensus was achieved via unstructured discussions until all authors were in agreement. This resulted in four child and five system attributes. The attributes are listed in Tables 1 and 2, together with non-specialist descriptions for the benefit of the general reader. (For a further introduction see Beukelman and Mirenda.[17])

In summary, the child attributes capture a child's language ability, experience with AAC, attitude/motivation to communicate with AAC, and whether the child is expected to regress, plateau or progress in communication ability. A total of 54 child vignettes were formed from the set of child attributes. Authors with expertise in AAC and speech and language therapy identified and removed 18 child vignettes representing unrealistic combinations, leaving 36.

AAC system attributes broadly captured the vocabulary set(s) provided by manufacturers, vocabulary size and organisation, type of graphic symbols used, and how consistent the navigational layout of words/symbols is when accessing items. It was not stated whether a system was high-tech or low-tech, although certain levels

For peer review only - http://bmjopen.bmj.com/site/about/guidelines.xhtml

(e.g., vocabulary sets with staged progression) are more common with high-tech systems. Authors with experience in AAC and speech and language therapy removed 158 unrealistic combinations from the 432 AAC systems which could be formed from the system attributes, leaving 274.

Prior experience from the BWS study suggested it would be difficult to recruit a large respondent sample, so to maximise the information captured a relatively heavy response burden of 12 choices between three systems was selected for the DCE. Participants were shown three child vignettes, referred to as Child A, B and C, and made four choices for each child vignette. An example task is shown in Appendix B.

The survey's statistical design (i.e., which levels of the AAC system attributes were presented in each question) was generated using NGene[i], with 60 choice tasks split into five blocks. The design sought to maximise D-efficiency, a measure of how much information it is possible to extract from survey responses.[49]

The survey was piloted by five AAC professionals and consequently the wording of some attributes and levels altered.

Survey administration

The DCE was administered online for ease of recruitment. Recruitment was carried out via AAC professionals email distribution lists (the project's own list and the mailing list of the UK wide charity Communication Matters www.communicationmatters.org.uk). In addition, invitations were sent via publicly available lists and websites, and the professional contacts of authors. Adverts were also placed on the project website and online media. Responses were collected between 20/10/17 and 4/3/18. Ethical approval was received from an NHS Research Ethics Committee (REC reference 6/NW/0165) and informed consent obtained from participants at the start of the survey.

[i] ©ChoiceMetrics

For peer review only - http://bmjopen.bmj.com/site/about/guidelines.xhtml

Participants began by confirming they contributed towards AAC decision-making for children, and those who indicated they did not answered only demographic questions.[i] Three child vignettes and one survey block were randomly allocated to each participant. The order of system attributes was randomised between participants, but consistent within participants, and which systems appeared on the left, middle and right of the screen was also randomised. At the end of the survey, participants answered demographic questions (for details, see Appendix A).

Analysis

Analysis of participants' choices was grounded in random utility theory. This standard approach[50] assumes participants choose the object which maximises their utility. The utility of an object is modelled as depending partly on the object's attributes and partly random, the latter component capturing the influence of all factors not included in the model. In a given choice scenario t, participant i chooses which of three AAC systems to allocate to child c. The utility to participant i of allocating AAC system $s \in \{1,2,3\}$ to child c in choice scenario t is

$$u_{isc} = \alpha_s + \beta_{ic} x_s + \varepsilon_i$$

where α_s is an alternative specific constant for AAC system s, x_s is a vector of dummy variables indicating AAC system levels, β_{ic} is a vector of coefficients which differ across participants and children, and ε_i is a random error term.

[i] The precise wording of the question was: *"I confirm my work involves assessing children for aided AAC systems and I contribute to the decision making in relation to the language and vocabulary organisation within AAC systems."* During testing it was revealed that some AAC professionals did not have sufficient input into the decision making process in their day-to-day practice for the DCE questions to be meaningful (e.g., occupational therapists specialising in optimising physical access to an AAC system recommended by other members of the team), and this question was designed to filter out such respondents.

For peer review only - http://bmjopen.bmj.com/site/about/guidelines.xhtml

The coefficient on level l of system attribute a, β_{ialc}, depends of the characteristics of the child vignette according to

$$\beta_{ialc} = \gamma_{ial0} + \gamma_{ial} z_c$$

where γ_{ial0} is a constant giving the preference for a system attribute at baseline child levels, z_c is a vector of dummy variables indicating vignette levels and γ_{ial} is a vector of coefficients, allowing for heterogeneity in relative preference for AAC system attributes depending on child characteristics.

A full model with all interaction terms included too many parameters to estimate reliably. Thus, parameters were eliminated in a stepwise process and a final preferred mixed logit model was identified using the Bayesian Information Criterion. The mixed logit model incorporates participant heterogeneity by allowing AAC system attribute parameters to be random, following a normal distribution with both means and variances depending on child characteristics. For details, see Appendix C.

Models were estimated using the CMC Choice Modelling Centre Code for R version 1.1[51] and all analysis was carried out using R version 3.3.1. Statistical significance was assessed at the 5% level after adjusting for multiple testing using Holm's sequential Bonferroni correction.[52]

Results are presented using a new measure termed *relative interaction attribute importance* (RIAI) which assesses how big an impact child attributes have on AAC professionals' decision-making. It is analogous to relative attribute importance, often used to present DCE results, [53] and may be calculated either with respect to a single choice object attribute or overall with respect to all choice object attributes. For a formal definition of RIAI, see Appendix D.

Patient and public involvement

One author (SM) is an AAC user, and one (LM) is the parent of an AAC user, and both were involved in all stages of research development and delivery.

RESULTS

For peer review only - http://bmjopen.bmj.com/site/about/guidelines.xhtml

A DCE on AAC professionals' decision making

A total of 172 participants completed the survey, of whom 155 indicated they contributed to decision-making regarding AAC systems and answered DCE questions. Summary statistics of their demographics and professional experience are given in Table 3. Most participants were female (~90%) and white. We believe this to be reasonably representative of the population of AAC professionals in the UK.[i] The mean age of DCE participants was around 40, with a range from 24 to 65, and they had 10 years' experience on average of AAC. Around 75% of DCE participants had a speech and language therapy background, with no other background reported by more than 10%. Those who did not answer DCE questions were less likely to have a speech and language therapy background (~50%), with teacher (~20%) and occupational therapist (~30%) more common.

Approximately 30% of the sample worked with all age groups, while 50-60% worked with pre-school, primary school and secondary school aged children. Participants were asked for the three most common diagnoses encountered in their work, with ~80% stating physical disability, 70% stating intellectual disability/developmental delay and 65% stating autism spectrum.

Turning to DCE responses, respondents chose the left-hand option 37.6% of the time, and the central and right-hand options 33.1% and 29.2% of the time respectively, significantly different from an equal distribution (one sample Kolmogorov-Smirnov p = 0.002).

Table 4 contains the results of the final preferred model, with 24 coefficients. Figure 1 illustrates the RIAI of child attributes for each system attribute and overall. The "constant" terms in Table 4 give participants' preferences for AAC system allocation when shown a child vignette with all attributes at baseline levels, which represents what was considered by the researchers as the most challenging profile that can be formed from the set of child attributes. This baseline vignette is as follows: *"Child A/B/C has delayed expressive and receptive language and no previous AAC experience. Child A/B/C does not appear motivated to communicate through any methods and means. Child A/B/C is predicted to regress in skills and abilities (regression)."*

[i] E.g., data from the Health and Care Professionals Council showed speech and language therapists in the UK were 96% female and the Higher Education Statistics Agency found speech and language therapy students in 2017/18 were 79% white. Source: Royal College of Speech and Language Therapists, personal communication.

For peer review only - http://bmjopen.bmj.com/site/about/guidelines.xhtml

The interaction terms represent how respondents' preferences for AAC systems change if choosing for a child vignette which differs on a given child attribute.

Vocabulary Sets

For the baseline child vignette, vocabulary sets which are fixed or have staged progression were preferred to no pre-installed vocabulary. Only a single child attribute influenced preferences: Professionals were much more likely compared to the baseline to choose systems with staged progression vocabulary sets over no pre-installed set if the child vignette was predicted progress in skills and ability (odds ratio, OR 3.88) (Table 4).

Consistency of layout,

For the baseline child vignette, consistent layout or an idiosyncratic layout was preferred to only having some aspects of system layout consistent for use, with no interactions with child attributes (Table 4).

Vocabulary organisation

For the baseline child vignette there was no significant preference between visual scene, taxonomic or semantic-syntactic vocabulary organisation, whilst pragmatic organisation was preferred. Two significant interactions exist between vocabulary organisation and motivation. A child vignette with motivation to communicate using AAC became more likely to be allocated a system with taxonomic (OR 2.03), or semantic-syntactic (OR 2.29) organisation compared to visual scene layout (Table 4).

Size of vocabulary

For the baseline child vignette there were no significant differences in preferences between up to 50 and between 50-1000 vocabulary items, but over 1000 items were considered significantly less appropriate. A mid-size vocabulary (50-1000 items) became more preferable compared to 50 or fewer for a child vignette motivated to communicate using AAC. Over 1000 items became significantly more preferable for child vignettes with each of the following characteristics: receptive language exceeding expressive language, an ability to use a range of AAC functions, motivated to communicate using AAC and predicted to progress

For peer review only - http://bmjopen.bmj.com/site/about/guidelines.xhtml

(Table 4). All child attributes influenced preferences for vocabulary size. As measured using RIAI, communication ability with AAC (32%) and determination and persistence (28%) were relatively more important than future skills and abilities (22%) and receptive and expressive language (17%) (Figure 1).

Graphic representation

For the baseline child vignette there was no preference between graphic representation using photos or pictographs, but text was less preferred than either, and idiographic symbols were even less preferred. Interactions were seen with two child attributes. Motivation to communicate using AAC increased the probability of choosing pictographic symbols (OR 3.88), idiographic symbols (OR 5.31) or text (OR 4.00) rather than photos. However, being predicted to progress made pictographic symbols less preferable (Table 4).

Overall RIAI of child attributes

Overall, future skills and abilities had the highest RIAI (38%), followed by child's determination and persistence (19%), communication ability with AAC (20%), and receptive and expressive language (12%) (Figure 1).

DISCUSSION

This DCE has revealed AAC professionals' priorities when choosing AAC systems for children, and shown that these priorities change when faced with children with different characteristics. This is not unexpected, and in line with previous research showing that AAC professionals recognise the importance of matching an AAC system to an individual person's needs.[22 54] However, this study builds on previous findings by showing the magnitude of preference changes, as for some system attributes their preferences for different levels could completely reverse depending which child vignette was shown. For example, for the baseline child vignette (see Table 4), a system with more than 1000 vocabulary items was less likely to be chosen than one with less than 50 (OR 0.395). However, for a child vignette describing a receptive-expressive language gap, the ability to use AAC for a range of functions, motivation to use AAC and predicted progression, a system with more

For peer review only - http://bmjopen.bmj.com/site/about/guidelines.xhtml

than 1000 vocabulary items was more likely to be chosen (OR 22.5). Such flexibility is encouraging, as it is in line with one of Williams et al.'s[14] five principles for AAC application: "AAC systems must be highly individualised and appropriate to individual needs" (p.195).

A key finding was that the attribute of the child's determination and persistence had the greatest number of interactions with preferences and was more important in terms of RIAI than language ability or previous experience with AAC. Specifically, the attribute level motivation to communicate using AAC tended to drive participants towards what can be regarded as more "ambitious" choices, for example more vocabulary items. It may be that participants believed that motivated children are more likely to succeed with such AAC systems, in line with previous findings that attitude towards AAC, and valuing an AAC system are important factors in successful adoption of AAC.[22 54]

Visual scene vocabulary organisation and graphic representation using photos can both involve items/scenes from an individual's own life, and use literal, rather than abstract depictions. Both were less preferred for child vignettes motivated to communicate via AAC. Rather, participants favoured more abstract methods of organisation (taxonomic and semantic-syntactic) and graphic symbols that require more grammar (pictographs, ideographs and text). This may be interpreted as an (unfounded[55]) belief that motivated children will be better able to use more complex AAC systems. An alternative and by no means mutually exclusive interpretation is that lack of motivation requires an AAC system involving familiar cues from their everyday environment.

Previous studies have also studied how AAC professionals choose graphic symbols for children.[56] For example, Thistle and Wilkinson[33] found that cognitive abilities are an important factor, as did Dada et al.[46] The advantage of a DCE is that the precise interactions between child characteristics and symbol type have been enumerated, showing, e.g., which children were more likely to be given AAC systems with photos, and which were more likely to be given systems with text.AAC system preferences did not significantly differ between child vignettes where their skills and abilities were predicted to regress or plateau. However, if a child was predicted to progress, this had a large impact on professional decision-making, with anticipated future skills and abilities ranked as the highest attribute in terms of RIAI. As with motivation, skills and

For peer review only - http://bmjopen.bmj.com/site/about/guidelines.xhtml

abilities led to more ambitious choices, with more vocabulary items preferred and pictographs depreciated compared to ideographs and text. This could reflect participants wishing to provide AAC systems that would fulfil the future needs of children who are anticipated to progress, given the large investment that goes into learning a new AAC system.[57-59] With plateau or regression this was less of a concern.

Photos were still the most preferred aided communication mode unless a child vignette featured both predicted progress *and* motivation to communicate via AAC. This finding possibly indicates that photos remain a good starting point for a child who is not engaged, regardless of prognosis, and may reflect recommendations that recognise the need to reduce the learning demands of AAC systems for some children.[12 60]

Despite unwelcome rates of abandonment, AAC professionals had high expectations of motivated children who were expected to progress, even if their receptive and expressive language were both delayed and they had no previous AAC experience. It has previously been noted that people who use AAC experience an asymmetry between the language they receive and the language they are able to express.[61] One interpretation is that participants wished to minimise asymmetries by choosing text as the expressive output for children they believed could cope with it. These ambitious choices are also encouraging given the greatly increased aspirations for effective societal participation of AAC users.[11 15 16] It is also in line with official guidance[62] and one of Williams et al.'s[14] five principles for AAC: "AAC must support full participation in all aspects of 21st century life" (p.195).

For many of the child vignettes there were non-linear preferences for vocabulary size. Offering between 50 and 1000 items was considered better than 50 or fewer for all child vignettes, although the difference was not always significant. This finding may indicate that participants were mindful of limiting children's potential for expression, even for children with lower cognitive ability and poor prognosis.

Findings suggest that respondents preferred levels of AAC systems that require personalisation, e.g., pragmatic vocabulary organisation or an idiosyncratic layout. This is in line with previous findings that personalisation is important in successful AAC adoption.[28] It indicates that it is not possible to achieve the

For peer review only - http://bmjopen.bmj.com/site/about/guidelines.xhtml

goal of AAC systems being closely tailored to individuals' needs[14 63] with "off-the-shelf" AAC systems: in other words, some personalisation is always necessary.[64 65]

Pre-installed vocabulary sets were always preferred over no pre-provided set, in line with other studies showing that selecting core vocabulary was an important part of AAC professionals' decision-making process.[33 37]

Comparing the DCE results with the previous BWS Case 1 study,[45] some similarities may be observed. For example, graphic representation was the lowest ranked attribute in terms of importance in the BWS to be included in the DCE. In concordance with this finding, when the relative importance of AAC system attributes was calculated for each child vignette in the DCE, graphic representation was never the most important attribute. This raises debate about the fundamental components of language construction through aided means and suggests much further research is required.

Many differences to the BWS findings can also be seen. Language abilities were the most important child attribute in the BWS, yet its RIAI in the DCE was below predicted future abilities, ranked sixth in the BWS. However, differences do not necessarily imply contradiction, as the two methodologies do not measure the same thing. The BWS measured the importance of AAC system attributes over the case mix AAC professionals encounter in practice, whereas for the DCE respondents were presented with a specific child vignette.

Receptive and expressive language had the lowest RIAI overall, with only a single interaction term in the final model. This contrasts with some previous findings that a child's language abilities play a large role in selecting an appropriate AAC System.[13 28 30 37] One possible explanation is that the aspects of language ability which were most relevant were captured in this study by other child attributes, but this remains a question to be addressed by future research.

This study has demonstrated the feasibility of conducting a DCE with a target population of AAC professionals. This is noteworthy given the relative rarity of DCEs studying health professionals' decision-making. For

For peer review only - http://bmjopen.bmj.com/site/about/guidelines.xhtml

example in a systematic review[41] of DCEs in health published between 2013 and 2017, only 13% included a sample of health professionals. In addition, there were particular challenges associated with performing a DCE with AAC professionals. The target population in the UK is small, meaning it was uncertain that sufficient participants for a successful study could be recruited. There were also concerns that participants might not find the DCE format acceptable, as they might reject having to make compromises between AAC system attributes in the context of providing a system for a child. Yet despite informal feedback that some respondents found the tasks uncomfortable, many were still willing to complete them. Finally, as interactions between child characteristics and AAC systems are so important, it was necessary to present hypothetical child vignettes, making tasks more complicated than in a typical DCE.

Despite these potential pitfalls, the DCE was successfully carried out, and having demonstrated the feasibility of the method in this area, further DCE studies should be considered in future.

Limitations

This study has several limitations. The sample size was relatively small (155 participants, compared to a median for healthcare DCEs of 401[41]). However, many studies exist with smaller sample sizes (e.g., Spinks et al.[66] with 35), and it was possible to estimate robust statistical models. Furthermore, it would have been difficult to collect a larger sample, as 155 participants represents a large proportion of the population of AAC professionals in the UK working with children, which is estimated at around 800.[i]

The DCE task may not match how UK AAC professionals make decisions in practice. Typically, many participants have the opportunity to work with families and children, as well as part of an AAC team, which could include diverse areas of clinical and personal expertise. Teams also generally make recommendations, rather than unilaterally choosing a system. However, there is evidence that AAC professionals compare the attributes of AAC systems in everyday practice,[13] and that they make trade-offs between system attributes,[37] akin to DCE tasks. In addition, it is still useful to study the individual decision-making of AAC professionals.

[i] Communication Matters, personal correspondence

For peer review only - http://bmjopen.bmj.com/site/about/guidelines.xhtml

Lynch et al.[30] report that a wide variety of team structures are used, and the mode of service delivery can have an influence on outcomes. Gathering evidence on individual-level decision-making can thus inform an assessment on how different ways of organising services influence decisions.

The DCE tasks presented one-off static decisions made by a single individual. In reality the decision-making environment is dynamic, with children developing over time, and often having two or more devices over the course of their childhood. These differences are a limiting factor in the external validity of results.

Attributes and levels use a mixture of speech and language therapy terms (e.g., receptive and expressive language) and more AAC specific language (e.g., staged vocabulary progression). This may have made it more difficult for respondents from any one professional speciality to interpret all of them.[44] However, this issue is not limited to the current study, but reflects an ongoing struggle in AAC to establish a common language.[44] In addition, respondents may have been unfamiliar with the generic term ideographic symbols, since only a single commercial set of ideographic symbols is in popular use in the UK (Minspeak[i]).

Respondents were more likely to choose AAC systems on the left of the screen and less likely to choose ones on the right. However, the risk of bias was mitigated by allowing for alternative specific constants and randomising the position in which AAC systems were presented.

Compared to the real children AAC professionals encounter, the child vignettes were simple, and lacked information which influences decision-making, such as the child's preferences[33] and contextual factors.[30] However, this is an inherent limitation of the DCE methodology, and vignettes with a greater number of attributes and levels would have made decisions overly burdensome, and therefore were not included. Significant interactions between AAC systems and child attributes implied that the vignettes were meaningful enough that respondents changed their preferences in response to them, often dramatically.

For a given child vignette, it was only possible to determine relative preferences for system attributes, rather than absolute preferences. Consequently, it is not possible to tell how suitable a given system is for a given

[i] © Semantic Compaction Systems, Inc.

For peer review only - http://bmjopen.bmj.com/site/about/guidelines.xhtml

child vignette, which is important as some presented a challenging profile for which it may be hard to find a suitable AAC system.

CONCLUSION

A lack of rigorous evidence on how to best assess and provide AAC systems for children has previously been identified,[25 34 44] as well as a gap between research and current practice.[11] In the light of this, the current study's results are encouraging, as it shows AAC professionals following best practice in many areas, for example ensuring AAC systems suit individual needs, and having high expectations for many children.

However, there is still demand from AAC professionals for better support in decision-making,[33 37] and undoubtedly current practice could be improved. The results of this study, together with evidence from the wider research project, have been used to create a heuristic and suite of resources, available at https://iasc.mmu.ac.uk. It is hoped these resources will aid AAC professionals in their clinical practice and help them provide the best possible service for children.

Acknowledgements: Thank you to Muireann McCleary and the Speech and Language Therapy team at the Central Remedial Clinic, Dublin, who piloted and gave feedback on the survey, and to participants who responded to the survey. Thanks to Mark Jayes (Manchester Metropolitan University) and Berenice Napier (Royal College of Speech and Language Therapists) for assistance in finding demographic statistics on speech and language therapists in the UK.

Funding: This independent research was funded by the National Institute for Health Research, UK (Health Services & Delivery Research Project: 14/70/153 - Identifying appropriate symbol communication aids for children who are non-speaking: enhancing clinical decision-making). The views expressed in this article are those of the authors and not necessarily those of the NHS, the National Institute for Health Research or the Department of Health.

Stephane Hess acknowledges additional support by the European Research Council through the consolidator grant 615596-DECISIONS.

For peer review only - http://bmjopen.bmj.com/site/about/guidelines.xhtml

Competing interests: The authors have no competing interests to declare.

Author statement: All authors conceived the study and defined the study aims. EW, DM, YL, NR, SJ, JG, SM, LM and JM developed attributes and levels. EW, DM and SH constructed the survey statistical design. EW and DM collected data. EW conducted statistical analysis. EW, YL, NR, SJ, JG, SM, LM and JM interpreted findings. EW wrote the manuscript first draft. All authors contributed to and approved the final manuscript.

Ethical approval: Ethical approval was received for the study from an NHS Research Ethics Committee (REC reference 6/NW/0165) and informed consent was obtained from participants at the start of the survey.

Data availability: Survey data is not publicly available as respondent consent was not obtained for this. However, it is available on request to the corresponding author or to Leeds Institute of Health Sciences if a formal data sharing agreement is entered into.

For peer review only

For peer review only - http://bmjopen.bmj.com/site/about/guidelines.xhtml

REFERENCES

1. Murray J, Goldbart J. Augmentative and alternative communication: a review of current issues. *Paediatrics and child health* 2009;19(10):464-68.

2. Schlosser RW, Wendt O. Effects of augmentative and alternative communication intervention on speech production in children with autism: A systematic review. *American Journal of Speech-Language Pathology* 2008;17(3):212-30.

3. Millar DC, Light JC, Schlosser RW. The impact of augmentative and alternative communication intervention on the speech production of individuals with developmental disabilities: A research review. *Journal of Speech, Language, and Hearing Research* 2006;49(2):248-64.

4. Lund SK, Light J. Long-term outcomes for individuals who use augmentative and alternative communication: Part I–What is a "good" outcome? *Augmentative and Alternative Communication* 2006;22(4):284-99.

5. Gross J. Augmentative and alternative communication: a report on provision for children and young people in England: Office of the Communication Champion, 2010.

6. Enderby P, Judge S, Creer S, et al. Examining the Need for and Provision of AAC Methods in the UK. *Advances in Clinical Neuroscience & Rehabilitation* 2013;13:20-23.

7. Judge S, Enderby P, Creer S, et al. Provision of powered communication aids in the United Kingdom. *Augmentative and Alternative Communication* 2017;33(3):181-87.

8. Hajjar DJ, McCarthy JW, Benigno JP, et al. "You Get More Than You Give": Experiences of Community Partners in Facilitating Active Recreation with Individuals who have Complex Communication Needs. *Augmentative and Alternative Communication* 2016;32(2):131-42.

9. Ryan SE, Shepherd T, Renzoni AM, et al. Towards Advancing Knowledge Translation of AAC Outcomes Research for Children and Youth with Complex Communication Needs. *Augmentative and Alternative Communication* 2015;31(2):137-47.

For peer review only - http://bmjopen.bmj.com/site/about/guidelines.xhtml

10. Dada S, Alant E. The effect of aided language stimulation on vocabulary acquisition in children with little or no functional speech. *American Journal of Speech-Language Pathology* 2009;18(1):50-64. doi: 10.1044/1058-0360(2008/07-0018)

11. Light J, McNaughton D, Beukelman D, et al. Challenges and opportunities in augmentative and alternative communication: Research and technology development to enhance communication and participation for individuals with complex communication needs. *Augmentative and Alternative Communication* 2019;35(1):1-12.

12. Light J, McNaughton D, Caron JJA, et al. New and emerging AAC technology supports for children with complex communication needs and their communication partners: State of the science and future research directions. *Augmentative and Alternative Communication* 2019;35(1):26-41.

13. Lund SK, Quach W, Weissling K, et al. Assessment with children who need augmentative and alternative communication (AAC): Clinical decisions of AAC specialists. *Language, Speech, and нearing Services in Schools* 2017;48(1):56-68.

14. Williams MB, Krezman C, McNaughton DJA, et al. "Reach for the stars": Five principles for the next 25 years of AAC. *Augmentative and Alternative Communication* 2008;24(3):194-206.

15. Calculator SNE. Augmentative and alternative communication (AAC) and inclusive education for students with the most severe disabilities. *International Journal of Inclusive Education* 2009;13(1):93-113.

16. Isakson CL, Burghstahler S, Arnold AJATO, et al. AAC, Employment, and Independent Living: A Success Story. *Assistive Technology Outcomes and Benefits* 2006;3(1):67-79.

17. Beukelman DR, Mirenda P. Augmentative and alternative communication: Supporting children and adults with complex communication needs: Paul H. Brookes Pub. 2013.

18. Williams M, Beukelman D, Ullman C. AAC text messaging. *Perspectives on Augmentative and Alternative Communication* 2012;21(2):56-59.

19. Sundqvist A, Rönnberg JJA, communication a. A qualitative analysis of email interactions of children who use augmentative and alternative communication. *Augmentative and Alternative Communication* 2010;26(4):255-66.

For peer review only

20. Hemsley B, Murray J. Distance and proximity: research on social media connections in the field of communication disability. *Disability and Rehabilitation* 2015; 37:17, 1509-1510.

21. Hynan A, Goldbart J, Murray J. A grounded theory of Internet and social media use by young people who use augmentative and alternative communication (AAC). *Disability and Rehabilitation* 2015;37(17):1559-75.

22. Johnson JM, Inglebret E, Jones C, et al. Perspectives of speech language pathologists regarding success versus abandonment of AAC. *Augmentative and Alternative Communication* 2006;22(2):85-99.

23. Moorcroft A, Scarinci N, Meyer C. A systematic review of the barriers and facilitators to the provision and use of low-tech and unaided AAC systems for people with complex communication needs and their families. *Disability and Rehabilitation: Assistive Technology* 2018:1-22.

24. Reddington J. The Domesday dataset: Linked open data in disability studies. *Journal of Intellectual Disabilities* 2013;17(2):107-21.

25. Munton T. Augmentative and Alternative Communication (AAC) support in Scotland: A review of the research literature and cost benefit analyses: NHS Education for Scotland, 2013.

26. Binger C, Ball L, Dietz A, et al. Personnel roles in the AAC assessment process. *Augmentative and Alternative Communication* 2012;28(4):278-88.

27. Lindsay S. Perceptions of health care workers prescribing augmentative and alternative communication devices to children. *Disability and Rehabilitation: Assistive Technology* 2010;5(3):209-22.

28. Dietz A, Quach W, Lund SK, et al. AAC assessment and clinical-decision making: The impact of experience. *Augmentative and Alternative Communication* 2012;28(3):148-59.

29. Guidance for commissioning AAC services and equipment: NHS England, 2016.

30. Lynch Y, Murray J, Moulam L, et al. Decision-making in communication aid recommendations in the UK: cultural and contextual influencers. *Augmentative and Alternative Communication* 2019:1-13.

31. Binger C, Light J. The effect of aided AAC modeling on the expression of multi-symbol messages by preschoolers who use AAC. *Augmentative and Alternative Communication* 2007;23(1):30-43.

For peer review only - http://bmjopen.bmj.com/site/about/guidelines.xhtml

32. Binger C, Light J. The morphology and syntax of individuals who use AAC: Research review and implications for effective practice. *Augmentative and Alternative Communication* 2008;24(2):123-38.

33. Thistle JJ, Wilkinson KM. Building evidence-based practice in AAC display design for young children: Current practices and future directions. *Augmentative and Alternative Communication* 2015;31(2):124-36.

34. Resource manual for commissioning and planning services for SLCN: Royal College of Speech and Language Therapists, 2009.

35. Batorowicz B, Shepherd TA. Teamwork in AAC: Examining clinical perceptions. *Augmentative and Alternative Communication* 2011;27(1):16-25.

36. Bryen DN, Chung Y, Lever SJPoA, et al. What you might not find in a typical transition plan! Some important lessons from adults who rely on augmentative and alternative communication. *Perspectives on Augmentative and Alternative Communication* 2010;19(2):32-40.

37. Murray J, Lynch Y, Meredith S, et al. Professionals' decision-making in recommending communication aids in the UK: competing considerations. *Augmentative and Alternative Communication* 2019:1-13.

38. Baxter S, Enderby P, Evans P, et al. Barriers and facilitators to the use of high-technology augmentative and alternative communication devices: a systematic review and qualitative synthesis. *International Journal of Language & Communication Disorders* 2012;47(2):115-29. doi: 10.1111/j.1460-6984.2011.00090.x

39. Geytenbeek JJ, Vermeulen RJ, Becher JG, et al. Comprehension of spoken language in non-speaking children with severe cerebral palsy: an explorative study on associations with motor type and disabilities. *Developmental Medicine & Child Neurology* 2015;57(3):294-300.

40. Choi BC, Pak AW. Multidisciplinarity, interdisciplinarity and transdisciplinarity in health research, services, education and policy: 1. Definitions, objectives, and evidence of effectiveness. *Clinical and Investigative Medicine* 2006;29(6):351.

41. Soekhai V, de Bekker-Grob EW, Ellis AR, et al. Discrete choice experiments in health economics: past, present and future. *PharmacoEconomics* 2019;37(2):201-26.

For peer review only - http://bmjopen.bmj.com/site/about/guidelines.xhtml

42. Clark MD, Determann D, Petrou S, et al. Discrete Choice Experiments in Health Economics: A Review of the Literature. *PharmacoEconomics* 2014;32(9):883-902. doi: 10.1007/s40273-014-0170-x

43. Ryan M. Discrete choice experiments in health care. *BMJ* 2004;328:360.

44. Judge S, Randall N, Goldbart J, et al. The language and communication attributes of graphic symbol communication aids–a systematic review and narrative synthesis. *Disability and Rehabilitation: Assistive Technology* 019:1-11.

45. Webb EJ, Meads D, Lynch Y, et al. What's important in AAC decision making for children? Evidence from a best-worst scaling survey. *Augmentative and Alternative Communication* 2019· 35(2):80-94.

46. Dada S, Murphy Y, Tönsing KJA, et al. Augmentative and alternative communication practices: A descriptive study of the perceptions of South African speech-language therapists. *Augmentative and Alternative Communication* 2017;33(4):189-200.

47. Coast J, Al-Janabi H, Sutton EJ, et al. Using qualitative methods for attribute development for discrete choice experiments: issues and recommendations. *Health Economics* 2012;21(6):730-41. doi: 10.1002/hec.1739

48. Kløjgaard ME, Bech M, Søgaard R. Designing a stated choice experiment: the value of a qualitative process. *Journal of Choice Modelling* 2012;5(2):1-18.

49. Kuhfeld WF, Tobias RD, Garratt M. Efficient experimental design with marketing research applications. *Journal of Marketing Research* 1994;31(4):545-57.

50. Louviere JJ, Hensher DA, Swait JD. Stated choice methods: analysis and applications: Cambridge University Press 2000.

51. CMC. CMC choice modelling code for R: Choice Modelling Centre, University of Leeds, 2017.

52. Holm S. A simple sequentially rejective multiple test procedure. *Scandinavian Journal of Statistics* 1979;6(2):65-70.

53. Hauber AB, González JM, Groothuis-Oudshoorn CG, et al. Statistical methods for the analysis of discrete choice experiments: a report of the ISPOR Conjoint Analysis Good Research Practices Task Force. *Value in Health* 2016;19(4):300-15.

For peer review only - http://bmjopen.bmj.com/site/about/guidelines.xhtml

54. Light J, McNaughton D. Communicative competence for individuals who require augmentative and alternative communication: A new definition for a new era of communication? Augmentative and Alternative Communication, 2014;30:1, 1-18.

55. von Tetzchner S. Introduction to the special issue on aided language processes, development, and use: an international perspective. *Augmentative and Alternative Communication* 2018;34(1):1-15.

56. Lynch Y, McCleary M, Smith M, et al. Instructional strategies used in direct AAC interventions with children to support graphic symbol learning: A systematic review. *Child Language Teaching and Therapy* 2018;34(1):23-36.

57. Getting the literacy and language skills needed for employment: Teaching is the solution. Proceedings of the Eighth Pittsburgh Employment Conference for Augmented Communicators; 2001. Shout Press Pittsburgh, PA.

58. Rackensperger T, Krezman C, Mcnaughton D, et al. "When I first got it, I wanted to throw it off a cliff": The challenges and benefits of learning AAC technologies as described by adults who use AAC. *Augmentative and Alternative Communication* 2005;21(3):165-86.

59. Bailey RL, Parette, H Jr, Stoner JB, et al. Family members' perceptions of augmentative and alternative communication device use. *Language, Speech, and Hearing Services in Schools* 2006

60. Light J, Wilkinson KM, Thiessen A, et al. Designing effective AAC displays for individuals with developmental or acquired disabilities: State of the science and future research directions. *Augmentative and Alternative Communication* 2019;35(1):42-55.

61. Smith MM, Grove NCJCcfiwuAFrtep. Asymmetry in input and output for individuals who use AAC. *Communicative competence for individuals who use AAC: From research to effective practice* 2003:163-95.

62. SEND Code of Practice 0–25 Years London: Department for Education, Department of Health, 2015.

63. Light J, McNaughton DJA, Communication A. Putting people first: Re-thinking the role of technology in augmentative and alternative communication intervention. *Augmentative and Alternative Communication* 2013;29(4):299-309.

For peer review only - http://bmjopen.bmj.com/site/about/guidelines.xhtml

64. King G, Batorowicz B, Shepherd T, et al. Expertise in research-informed clinical decision making: Working effectively with families of children with little or no functional speech. *Evidence-Based Communication Assessment and Intervention* 2008;2(2):106-16.

65. Parette P, VanBiervliet A, Hourcade J. Family-centered decision making in assistive technology. *Journal of Special Education Technology* 1999;15(1):45-55.

66. Spinks J, Janda M, Soyer HP, et al. Consumer preferences for teledermoscopy screening to detect melanoma early. *Journal of Telemedicine and Telecare* 2016;22(1):39-46.

For peer review only

Table 1: Child attributes and levels including brief descriptions

Child attributes and levels	Description†
**Receptive and expressive language (1)**	Child's ability without AAC to understand communication from (receptive) and communicate with others (expressive)
*Delayed	Both receptive and expressive abilities below expectation given child's age
Receptive language exceeding expressive language	Ability to understand communication from others greater than ability to communicate with others
**Communication ability with AAC (3)**	How well a child can communicate when using AAC
*No previous AAC experience	Has never communicated using AAC before
Able to use AAC for a few communicative functions	Can use AAC for some basic functions, e.g. simple requests
Able to use AAC for a range of communicative functions	Can use AAC for more complex tasks, e.g. constructing sentences
**Child's determination and persistence (4)**	Attitude of child towards communication and using AAC
*Does not appear motivated to communicate through any methods and means	Child is not inclined to develop communication skills
Motivated to communicate through symbol communication systems	Child has demonstrated motivation and willingness to use AAC
Only motivated to communicate through methods other than symbol communication	Child may be motivated to communicate, but is not inclined to use AAC
**Predicted future skills and abilities (6)**	Professional assessment of how child's communication abilities will develop
*Regression	Abilities projected to become worse in future, e.g. due to a degenerative condition such as Rett syndrome
Plateau	Abilities will not change significantly in future, e.g. a child aged 16-17
Progression	Communication abilities will develop in future

Note: * indicates baseline level; numbers in parentheses indicate attributes' rank in relative importance from Webb et al. [45] †Descriptions are not intended as rigorous definitions of AAC terminology, but as a rough guide for the non-AAC specialist reader.

For peer review only - http://bmjopen.bmj.com/site/about/guidelines.xhtml

Table 2: AAC System attributes and levels, including brief descriptions

AAC System attributes and levels	Description†
**Vocabulary sets (1)**	Words and/or symbols pre-provided with system "out of the box", e.g. as part of a software package for a high-tech system
*No vocabulary set	AAC practitioners/child's support network provides all vocabulary content
Fixed vocabulary set	A single fixed set of vocabulary which may be customised
Vocabulary set with staged progression	A series of vocabulary sets with pre-determined progression through them that simulate language development. E.g. an initial set including just basic words, with subsequent sets introducing more grammatical structure. May be customised.
**Consistency of layout (2)**	How consistent positions of words/symbols are in system interface, and how consistent navigation to find different symbols is
*Consistency of some aspects of layout	Words/symbols in multiple categories appear in different positions across categories, but always in the same place in a given category
Consistency of all aspects of layout	All/nearly all words/symbols always appear in same position in interface
Idiosyncratic layout	Layout that has been personalised for an individual child
**Type of vocabulary organisation (5)**	How words/symbols are organised within the system
*Visual scene	Interface shows photos, most likely of scenes familiar to the child, with areas of it highlighted to represent words
Taxonomic	Words/symbols organised according to subject, analogous to non-fiction books in a library
Semantic-syntactic	Words/symbols organised according to sentence structure, e.g. verbs, nouns, adjectives
Pragmatic	Words/symbols organised around function in language rather than grammar, e.g. request, mood
**Size of vocabulary (7)**	How many words/symbols system can output
*Up to 50 vocabulary items	Implies only simple communication functions possible
50-1000 vocabulary items	Implies combining words/symbols to create grammatical structures
More than 1000 vocabulary items	Does not imply more complex communication than 50-1000 items, but means a greater load on child's memory
**Graphic representation (12)**	Type of symbols used by system
*Photos	Photographs, possibly of items or environments personal to the child
Pictographic symbol set	Non-photorealist pictures with specific meanings attached. May be accompanied by text
Ideographic symbol system (with rules or encoding)	Stylised symbols combined with fixed rules and grammar analogous to Chinese/Japanese characters, e.g. Minspeak
Text	Text unaccompanied by other symbols

Note: * indicates baseline level; numbers in parentheses indicate attributes' rank in relative importance from prior BWS study (reported in Webb et al. [45]). †Descriptions are not intended as rigorous definitions of AAC terminology, but as a rough guide for the non-AAC specialist reader.

For peer review only - http://bmjopen.bmj.com/site/about/guidelines.xhtml

Table 3: Demographics and professional experience of participants

		mean	s.e
Age (years)		40.8	11
Experience (years)		11.4	9.2
% of role relating to AAC		53.7	34.3
		N	%
Gender	Female	155	90.1
	Male	10	5.81
	Prefer not to say	7	4.07
Ethnicity	White - English/Welsh/Scottish/Northern Irish/British	149	86.6
	White – other	12	6.98
	Other	6	3.49
	White – Irish	5	2.91
Professional background	Speech and language therapist	125	72.7
	Occupational therapist	16	9.3
	Teacher	14	8.14
	Other	12	6.98
	Assistive technology specialist	5	2.91
	Clinical scientist	5	2.91
Age groups worked with	Primary school age	99	57.6
	Secondary school age	94	54.7
	Pre-school age	85	49.4
	All age groups	56	32.6
	Higher education	30	17.4
	Further education	21	12.2
	Other	12	6.98
	Adults	10	5.81
Among most common three diagnoses seen in practice	Physical disability (e.g. neuromuscular, cerebral palsy etc.)	140	81.4
	Intellectual Disability/Developmental Delay	118	68.6
	Autism spectrum disorder	113	65.7
	Syndromes	61	35.5
	Neurological	45	26.2
	Specific Speech/Language Impairment	22	12.8
	Dyspraxia	14	8.14

Note. For some questions, participants could select more than one response, thus some percentages do not sum to 100%

For peer review only

For peer review only - http://bmjopen.bmj.com/site/about/guidelines.xhtml

Table 4: Parameter means and standard deviations for final mixed logit model. * indicates significance at the 5% level corrected using Holm's sequential Bonferroni [52], s.e. = standard error

AAC system attribute		Child attribute	Parameter mean	s.e.	σ	s.e.
Vocabulary sets (baseline none)	Fixed	Constant	0.283*	0.0966	0.131	0.258
	Staged progression	Constant	0.364*	0.141	0.941*	0.206
		Predicted to progress	1.36*	0.221	-1.09*	0.343
Consistency of layout (baseline some aspects)	Consistency of all aspects	Constant	0.892*	0.121	0.15	0.126
	Idiosyncratic layout	Constant	1.46*	0.14	0.757*	0.134
Type of vocabulary organisation (baseline visual scene)	Taxonomic	Constant	0.0629	0.165	0.383	0.257
		Motivated to communicate through symbol communication systems	0.707*	0.206	-0.563	0.295
	Semantic-syntactic	Constant	-0.178	0.166	0.549	0.234
		Motivated to communicate through symbol communication systems	0.826*	0.197	-0.112	0.296
	Pragmatic	Constant	0.443*	0.123	0.723*	0.152
Size of vocabulary (baseline 50 items)	50-1000 items	Constant	0.131	0.143	0.43	0.166
		Motivated to communicate through symbol communication systems	1.01*	0.232	-0.731	0.329
	More than 1000 items	Constant	-0.929*	0.213	1.02*	0.33
		Receptive language exceeding expressive language	0.692*	0.186	0.489	0.367
		Able to use AAC for a range of communicative functions	1.14*	0.319	-0.419	0.762
		Motivated to communicate through symbol communication systems	1.31*	0.272	-0.751	0.556
		Predicted to progress	0.902*	0.233	0.981	0.657
Graphic representation (baseline photos)	Pictographic symbol set	Constant	-0.41	0.183	0.0722	0.248
		Motivated to communicate through symbol communication systems	1.36*	0.24	-0.363	0.428
		Predicted to progress	-0.814*	0.217	1.12	0.385
	Ideographic symbol system	Constant	-1.25*	0.207	0.823*	0.216
		Motivated to communicate through symbol communication systems	1.67*	0.268	0.069	0.297
	Text	Constant	-0.709*	0.159	0.615*	0.204
		Motivated to communicate through symbol communication systems	1.39*	0.231	-1.12*	0.282

Note. Constants give preferences when choosing for the baseline child vignette: *"Child A/B/C has delayed expressive and receptive language and no previous AAC experience. Child A/B/C does not appear motivated to communicate through any methods and means. Child A/B/C is predicted to regress in skills and abilities (regression)."*
σ indicates standard deviation. Parameter variance for level l of AAC system attribute a when choosing for child c is given by $\sigma^2_{alc} = (\sigma_{al0} + \sigma_{al} Z_c)^2$.

For peer review only - http://bmjopen.bmj.com/site/about/guidelines.xhtml

For peer review only

Figure 1: Relative interaction attribute importance for each AAC system attribute and averaged over all attributes. Note that consistency of layout is omitted as there are no interactions with child attributes. Error bars show 95% confidence intervals.

For peer review only

For peer review only - http://bmjopen.bmj.com/site/about/guidelines.xhtml

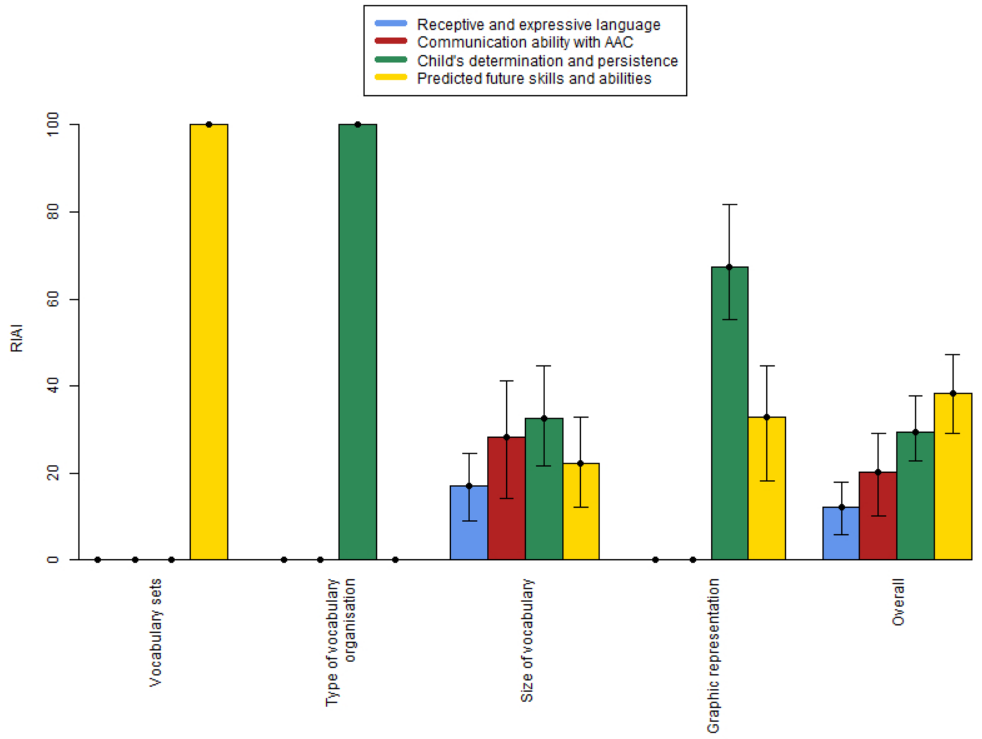

Relative interaction attribute importance for each AAC system attribute and averaged over all attributes. Note that consistency of layout is omitted as there are no interactions with child attributes. Error bars show 95% confidence intervals.

282x211mm (72 x 72 DPI)

Appendix A – Attributes from best-worst scaling case 1 study

Table B 1: Attributes used in best-worst scaling case 1 survey in Webb et al. [23] and rank in terms of relative importance score

Child attribute	Rank
*Child's receptive and expressive language abilities	1
Support for AAC from communication partners	2
*Communication ability with aided AAC	3
*Child's determination and persistence	4
Physical abilities for access	5
*Predicted future needs and abilities	6
Level of learning ability	7
Insight into own communicative skills	8
Attention level	9
Access to professional AAC support	10
Speech skills and intelligibility	11
Functional visual skills	12
History of aided AAC use	13
Presence of additional diagnoses	14
Level of fatigue	15
Literacy ability	16
Educational stage	17
Primary diagnosis	18
Mobility	19
**AAC system attributes**	**Rank**
*Vocabulary or language package(s)	1
*Consistency of layout and navigation	2
Ease of customization	3
Durability and reliability	4
*Type of vocabulary organization	5
Number of key presses required to generate symbol or text output	6
*Size of output vocabulary	7
Range of access methods	8
Number of cells per page	9
Portability	10
*Graphic representation	11
Battery life	12
Supplier support	13
Ease of mounting on a range of equipment	14
Cost	15
Additional assistive technology functions	16
Voice	17
Appearance	18

Note. Asterisk indicates attribute included in discrete choice experiment.

For peer review only

Appendix B – Example survey

Note: the AAC system levels and child vignettes shown here are for illustrative purposes and do not represent the statistical design used in the full survey.

Instructions

Thank you for taking part in this survey.

It aims to identify what factors clinicians think are important when making decisions about aided AAC systems for children with communication difficulties.

You will be asked a series of questions. Each one has the same format. A brief description of a child will be given, along with three possible choices of aided AAC systems.

The three AAC systems are described in terms of five characteristics (the systems are identical apart from changes to these five characteristics):-

1. **Vocabulary sets**: Pre-determined vocabulary or language package provided, which can be:-
   - No commercially provided sets
   - Commercially provided sets without language progression
   - Commercially provided sets with language progression
2. **Size of vocabulary**: The size of the output vocabulary available within the aided AAC system, which can be:-
   - Up to 50 vocabulary items
   - 50-1000 vocabulary items
   - More than 1000 vocabulary items
3. **Type of vocabulary organisation**: Primary format used to organise the vocabulary within the aided AAC system, which can be:-
   - Visual scene display
   - Semantic organisation
   - Semantic syntactic organisation
   - Pragmatic organisation

For peer review only

4. **Graphic Representation**: Primary type of graphic symbol used, which can be:-
    - Photo symbols (i.e. a photo symbol set without rules or encoding)
    - Pictographic symbols (i.e. a graphic symbol set without rules or encoding)
    - Ideographic symbols (i.e. a symbol system with rules or encoding)
    - Graphic symbols with text (i.e. a system with either pictographic or ideographic symbols that incorporates an alphabet for generating text)
5. **Consistency of layout**: Consistency of layout of symbols on pages, including when navigating through pages to select desired output, which can be:-
    - Inconsistent layout
    - Somewhat consistent layout
    - Highly consistent layout

Imagine you had to choose between only these three systems. You should indicate which you would prescribe for the child described. If your preferred option is not available, pick the system from the three options that you think best matches the child's needs. There are no right or wrong answers. It is acknowledged that this may feel uncomfortable for you.

In the survey, there are three different children described. You will be asked four questions about each child (12 questions in total).

In acknowledgement of choices being uncomfortable, after each choice, you will be asked to indicate how well you think that system matches the child's needs. (1 = very unsuitable, 7 = very suitable).

This survey is part of independent research funded by the National Institute for Health Research (NIHR), Health Service and Delivery Research (HS&DR) Programme 14/70/153. The views expressed are those of the authors and do not necessarily reflect those of the NHS, the NIHR, NETSCC, the HS&DR programme or the Department of Health.

Consent

For peer review only

Your participation in this survey is voluntary. All information is collected anonymously and held in confidence. We hope you complete the survey but you are free to stop responding at any point resulting in your answers will be removed.

❑ I have read and understood the above and consent to taking part.

I confirm my work involves assessing children for aided AAC systems and I contribute to the decision making in relation to the language and vocabulary organisation within aided AAC systems.

❑ Yes

❑ No

If yes go to DCE questions.

If no go to a page with the following:-

Thank you for your interest in this survey. At present we are only recruiting participants who contribute to decision making in relation to the language and vocabulary organisation within aided AAC for children. Over the coming 12 months we will be recruiting people with a wider range of AAC experience to test decision making resources we are developing. If you are interested in this aspect of the project or would like to be notified when the free resources are available, there will be an opportunity at the end to submit your email address.

We would still like to ask you a few questions about your experience with AAC to check the representativeness of participants.

Then go directly to demographics questionnaire.

For peer review only

Question 1

Child A has delayed expressive and receptive language and is able to use aided AAC for a few communicative functions. Child A is motivated to communicate through symbol communication systems. Child A is predicted to regress in skills and abilities (regression).

	System 1	System 2	System 3
**Size of vocabulary** The size of the output vocabulary available within the aided AAC system.	50-1000 vocabulary items	50-1000 vocabulary items	50-1000 vocabulary items
**Type of vocabulary organisation** Primary format used to organise the vocabulary within the aided AAC system	Pragmatic	Visual Scene	Visual Scene
**Vocabulary sets** Pre-determined vocabulary or language package provided	No vocabulary set	Vocabulary sets with staged progression	Fixed vocabulary set
**Graphic representation** Primary type of graphic symbol used	Ideographic symbol system (with rules or encoding)	Photos	Ideographic symbol system (with rules or encoding)
**Type of vocabulary organisation** Primary format used to organise the vocabulary within the aided AAC system	Idiosyncratic layout	Idiosyncratic layout	Consistency of some aspects of layout

For this child I would choose:

- ❑ System 1
- ❑ System 2
- ❑ System 3

On a scale from 1 to 7, how good a match is your chosen device for this child? (1=very unsuitable, 7=very suitable).

❑	❑	❑	❑	❑	❑	❑
1	2	3	4	5	6	7

For peer review only

Question 2

Child A has delayed expressive and receptive language and is able to use aided AAC for a few communicative functions. Child A is motivated to communicate through symbol communication systems. Child A is predicted to regress in skills and abilities (regression).

	System 1	System 2	System 3
**Size of vocabulary**<br>The size of the output vocabulary available within the aided AAC system.	50-1000 vocabulary items	50-1000 vocabulary items	50-1000 vocabulary items
**Type of vocabulary organisation**<br>Primary format used to organise the vocabulary within the aided AAC system	Taxonomic	Visual Scene	Pragmatic
**Vocabulary sets**<br>Pre-determined vocabulary or language package provided	Vocabulary sets with staged progression	No vocabulary set	Fixed vocabulary set
**Graphic representation**<br>Primary type of graphic symbol used	Text	Pictographic symbol set	Photos
**Type of vocabulary organisation**<br>Primary format used to organise the vocabulary within the aided AAC system	Consistency of some aspects of layout	Idiosyncratic layout	Consistency of some aspects of layout

For this child I would choose:

- ❑ System 1
- ❑ System 2
- ❑ System 3

On a scale from 1 to 7, how good a match is your chosen device for this child? (1=very unsuitable, 7=very suitable).

❑	❑	❑	❑	❑	❑	❑
1	2	3	4	5	6	7

For peer review only

Question 3

Child A has delayed expressive and receptive language and is able to use aided AAC for a few communicative functions. Child A is motivated to communicate through symbol communication systems. Child A is predicted to regress in skills and abilities (regression).

	**System 1**	**System 2**	**System 3**
**Size of vocabulary**<br>The size of the output vocabulary available within the aided AAC system.	50-1000 vocabulary items	50-1000 vocabulary items	Up to 50 vocabulary items
**Type of vocabulary organisation**<br>Primary format used to organise the vocabulary within the aided AAC system	Semantic-Syntactic	Semantic-Syntactic	Visual Scene
**Vocabulary sets**<br>Pre-determined vocabulary or language package provided	Vocabulary sets with staged progression	No vocabulary set	Fixed vocabulary set
**Graphic representation**<br>Primary type of graphic symbol used	Text	Photos	Pictographic symbol set
**Type of vocabulary organisation**<br>Primary format used to organise the vocabulary within the aided AAC system	Consistency of all aspects of layout	Idiosyncratic layout	Consistency of some aspects of layout

For this child I would choose:

- ❑ System 1
- ❑ System 2
- ❑ System 3

On a scale from 1 to 7, how good a match is your chosen device for this child? (1=very unsuitable, 7=very suitable).

❑	❑	❑	❑	❑	❑	❑
1	2	3	4	5	6	7

For peer review only

Question 4

Child A has delayed expressive and receptive language and is able to use aided AAC for a few communicative functions. Child A is motivated to communicate through symbol communication systems. Child A is predicted to regress in skills and abilities (regression).

	**System 1**	**System 2**	**System 3**
**Size of vocabulary**<br>The size of the output vocabulary available within the aided AAC system.	50-1000 vocabulary items	50-1000 vocabulary items	50-1000 vocabulary items
**Type of vocabulary organisation**<br>Primary format used to organise the vocabulary within the aided AAC system	Visual Scene	Taxonomic	Pragmatic
**Vocabulary sets**<br>Pre-determined vocabulary or language package provided	Vocabulary sets with staged progression	Fixed vocabulary set	Fixed vocabulary set
**Graphic representation**<br>Primary type of graphic symbol used	Ideographic symbol system (with rules or encoding)	Text	Photos
**Type of vocabulary organisation**<br>Primary format used to organise the vocabulary within the aided AAC system	Consistency of some aspects of layout	Consistency of all aspects of layout	Idiosyncratic layout

For this child I would choose:
- ❑ System 1
- ❑ System 2
- ❑ System 3

On a scale from 1 to 7, how good a match is your chosen device for this child? (1=very unsuitable, 7=very suitable).

❑	❑	❑	❑	❑	❑	❑
1	2	3	4	5	6	7

For peer review only

Question 5

Child B has receptive language exceeding expressive language and no previous AAC experience. Child B is only motivated to communicate through methods other than symbol communication systems. Child B is predicted to maintain current skills and abilities (plateau).

	System 1	System 2	System 3
**Size of vocabulary**<br>The size of the output vocabulary available within the aided AAC system.	Up to 50 vocabulary items	50-1000 vocabulary items	More than 1000 vocabulary items
**Type of vocabulary organisation**<br>Primary format used to organise the vocabulary within the aided AAC system	Taxonomic	Visual Scene	Pragmatic
**Vocabulary sets**<br>Pre-determined vocabulary or language package provided	No vocabulary set	Fixed vocabulary set	Vocabulary sets with staged progression
**Graphic representation**<br>Primary type of graphic symbol used	Photos	Text	Ideographic symbol system (with rules or encoding)
**Type of vocabulary organisation**<br>Primary format used to organise the vocabulary within the aided AAC system	Consistency of all aspects of layout	Idiosyncratic layout	Consistency of some aspects of layout

For this child I would choose:

- ❑ System 1
- ❑ System 2
- ❑ System 3

On a scale from 1 to 7, how good a match is your chosen device for this child? (1=very unsuitable, 7=very suitable).

❑	❑	❑	❑	❑	❑	❑
1	2	3	4	5	6	7

For peer review only

Question 6

Child B has receptive language exceeding expressive language and no previous AAC experience. Child B is only motivated to communicate through methods other than symbol communication systems. Child B is predicted to maintain current skills and abilities (plateau).

	System 1	System 2	System 3
**Size of vocabulary**<br>The size of the output vocabulary available within the aided AAC system.	More than 1000 vocabulary items	Up to 50 vocabulary items	50-1000 vocabulary items
**Type of vocabulary organisation**<br>Primary format used to organise the vocabulary within the aided AAC system	Semantic-Syntactic	Semantic-Syntactic	Taxonomic
**Vocabulary sets**<br>Pre-determined vocabulary or language package provided	Vocabulary sets with staged progression	Fixed vocabulary set	No vocabulary set
**Graphic representation**<br>Primary type of graphic symbol used	Ideographic symbol system (with rules or encoding)	Photos	Ideographic symbol system (with rules or encoding)
**Type of vocabulary organisation**<br>Primary format used to organise the vocabulary within the aided AAC system	Idiosyncratic layout	Consistency of some aspects of layout	Consistency of some aspects of layout

For this child I would choose:

- ❑ System 1
- ❑ System 2
- ❑ System 3

On a scale from 1 to 7, how good a match is your chosen device for this child? (1=very unsuitable, 7=very suitable).

❑	❑	❑	❑	❑	❑	❑
1	2	3	4	5	6	7

For peer review only

Question 7

Child B has receptive language exceeding expressive language and no previous AAC experience. Child B is only motivated to communicate through methods other than symbol communication systems. Child B is predicted to maintain current skills and abilities (plateau).

	**System 1**	**System 2**	**System 3**
**Size of vocabulary**<br>The size of the output vocabulary available within the aided AAC system.	Up to 50 vocabulary items	More than 1000 vocabulary items	50-1000 vocabulary items
**Type of vocabulary organisation**<br>Primary format used to organise the vocabulary within the aided AAC system	Taxonomic	Pragmatic	Taxonomic
**Vocabulary sets**<br>Pre-determined vocabulary or language package provided	Fixed vocabulary set	Vocabulary sets with staged progression	No vocabulary set
**Graphic representation**<br>Primary type of graphic symbol used	Photos	Ideographic symbol system (with rules or encoding)	Text
**Type of vocabulary organisation**<br>Primary format used to organise the vocabulary within the aided AAC system	Consistency of all aspects of layout	Consistency of some aspects of layout	Idiosyncratic layout

For this child I would choose:

- ❑ System 1
- ❑ System 2
- ❑ System 3

On a scale from 1 to 7, how good a match is your chosen device for this child? (1=very unsuitable, 7=very suitable).

❑	❑	❑	❑	❑	❑	❑
1	2	3	4	5	6	7

For peer review only

Question 8

Child B has receptive language exceeding expressive language and no previous AAC experience. Child B is only motivated to communicate through methods other than symbol communication systems. Child B is predicted to maintain current skills and abilities (plateau).

	**System 1**	**System 2**	**System 3**
**Size of vocabulary**<br>The size of the output vocabulary available within the aided AAC system.	Up to 50 vocabulary items	More than 1000 vocabulary items	50-1000 vocabulary items
**Type of vocabulary organisation**<br>Primary format used to organise the vocabulary within the aided AAC system	Pragmatic	Semantic-Syntactic	Visual Scene
**Vocabulary sets**<br>Pre-determined vocabulary or language package provided	Fixed vocabulary set	Fixed vocabulary set	Vocabulary sets with staged progression
**Graphic representation**<br>Primary type of graphic symbol used	Ideographic symbol system (with rules or encoding)	Ideographic symbol system (with rules or encoding)	Photos
**Type of vocabulary organisation**<br>Primary format used to organise the vocabulary within the aided AAC system	Consistency of some aspects of layout	Consistency of all aspects of layout	Idiosyncratic layout

For this child I would choose:

- ❑ System 1
- ❑ System 2
- ❑ System 3

On a scale from 1 to 7, how good a match is your chosen device for this child? (1=very unsuitable, 7=very suitable).

❑	❑	❑	❑	❑	❑	❑
1	2	3	4	5	6	7

For peer review only

Question 9

Child C has delayed expressive and receptive language and no previous AAC experience. Child C is only motivated to communicate through methods other than symbol communication systems. Child C is predicted to maintain current skills and abilities (plateau).

	System 1	System 2	System 3
**Size of vocabulary**<br>The size of the output vocabulary available within the aided AAC system.	50-1000 vocabulary items	More than 1000 vocabulary items	Up to 50 vocabulary items
**Type of vocabulary organisation**<br>Primary format used to organise the vocabulary within the aided AAC system	Visual Scene	Pragmatic	Semantic-Syntactic
**Vocabulary sets**<br>Pre-determined vocabulary or language package provided	Fixed vocabulary set	Fixed vocabulary set	Fixed vocabulary set
**Graphic representation**<br>Primary type of graphic symbol used	Pictographic symbol set	Pictographic symbol set	Text
**Type of vocabulary organisation**<br>Primary format used to organise the vocabulary within the aided AAC system	Idiosyncratic layout	Consistency of some aspects of layout	Consistency of all aspects of layout

For this child I would choose:

- ❑ System 1
- ❑ System 2
- ❑ System 3

On a scale from 1 to 7, how good a match is your chosen device for this child? (1=very unsuitable, 7=very suitable).

❑	❑	❑	❑	❑	❑	❑
1	2	3	4	5	6	7

Question 10

Child C has delayed expressive and receptive language and no previous AAC experience. Child C is only motivated to communicate through methods other than symbol communication systems. Child C is predicted to maintain current skills and abilities (plateau).

	**System 1**	**System 2**	**System 3**
**Size of vocabulary**<br>The size of the output vocabulary available within the aided AAC system.	Up to 50 vocabulary items	More than 1000 vocabulary items	Up to 50 vocabulary items
**Type of vocabulary organisation**<br>Primary format used to organise the vocabulary within the aided AAC system	Taxonomic	Semantic-Syntactic	Taxonomic
**Vocabulary sets**<br>Pre-determined vocabulary or language package provided	No vocabulary set	Vocabulary sets with staged progression	No vocabulary set
**Graphic representation**<br>Primary type of graphic symbol used	Ideographic symbol system (with rules or encoding)	Ideographic symbol system (with rules or encoding)	Photos
**Type of vocabulary organisation**<br>Primary format used to organise the vocabulary within the aided AAC system	Consistency of all aspects of layout	Consistency of some aspects of layout	Idiosyncratic layout

For this child I would choose:

- ❑ System 1
- ❑ System 2
- ❑ System 3

On a scale from 1 to 7, how good a match is your chosen device for this child? (1=very unsuitable, 7=very suitable).

❑	❑	❑	❑	❑	❑	❑
1	2	3	4	5	6	7

For peer review only

Question 11

Child C has delayed expressive and receptive language and no previous AAC experience. Child C is only motivated to communicate through methods other than symbol communication systems. Child C is predicted to maintain current skills and abilities (plateau).

	System 1	System 2	System 3
**Size of vocabulary** The size of the output vocabulary available within the aided AAC system.	Up to 50 vocabulary items	50-1000 vocabulary items	50-1000 vocabulary items
**Type of vocabulary organisation** Primary format used to organise the vocabulary within the aided AAC system	Visual Scene	Semantic-Syntactic	Semantic-Syntactic
**Vocabulary sets** Pre-determined vocabulary or language package provided	No vocabulary set	Vocabulary sets with staged progression	No vocabulary set
**Graphic representation** Primary type of graphic symbol used	Photos	Pictographic symbol set	Text
**Type of vocabulary organisation** Primary format used to organise the vocabulary within the aided AAC system	Idiosyncratic layout	Consistency of some aspects of layout	Consistency of all aspects of layout

For this child I would choose:

- ❑ System 1
- ❑ System 2
- ❑ System 3

On a scale from 1 to 7, how good a match is your chosen device for this child? (1=very unsuitable, 7=very suitable).

❑	❑	❑	❑	❑	❑	❑
1	2	3	4	5	6	7

For peer review only

Question 12

Child C has delayed expressive and receptive language and no previous AAC experience. Child C is only motivated to communicate through methods other than symbol communication systems. Child C is predicted to maintain current skills and abilities (plateau).

	System 1	System 2	System 3
**Size of vocabulary**<br>The size of the output vocabulary available within the aided AAC system.	Up to 50 vocabulary items	More than 1000 vocabulary items	50-1000 vocabulary items
**Type of vocabulary organisation**<br>Primary format used to organise the vocabulary within the aided AAC system	Semantic-Syntactic	Semantic-Syntactic	Visual Scene
**Vocabulary sets**<br>Pre-determined vocabulary or language package provided	No vocabulary set	Vocabulary sets with staged progression	Fixed vocabulary set
**Graphic representation**<br>Primary type of graphic symbol used	Ideographic symbol system (with rules or encoding)	Text	Photos
**Type of vocabulary organisation**<br>Primary format used to organise the vocabulary within the aided AAC system	Consistency of all aspects of layout	Idiosyncratic layout	Consistency of some aspects of layout

For this child I would choose:

- ❑ System 1
- ❑ System 2
- ❑ System 3

On a scale from 1 to 7, how good a match is your chosen device for this child? (1=very unsuitable, 7=very suitable).

❑ 1 ❑ 2 ❑ 3 ❑ 4 ❑ 5 ❑ 6 ❑ 7

For peer review only

Questionnaire

In this final part of the survey, we'd like to ask you for some information about yourself and your experience with AAC.

We would like to know about the characteristics of the people who complete this survey to check that we have a representative sample. We would also like to check if people with different professional experiences have different opinions.

All responses will be held anonymously and we have no way of tracing your responses back to you as an individual.

Q. What is your age? _______ years.

Q. What is your gender?

❑ Male ❑ Female ❑ Other ❑ Prefer not to say

Q. How would you describe your ethnicity?

- ❑ White - English/Welsh/Scottish/Northern Irish/British
- ❑ White -Irish
- ❑ White - Gypsy or Irish Traveller
- ❑ White - Any other White background
- ❑ Mixed/Multiple ethnic group - White and Black Caribbean
- ❑ Mixed/Multiple ethnic group - White and Black African
- ❑ Mixed/Multiple ethnic group - White and Asian
- ❑ Mixed/Multiple ethnic group - Any other Mixed/Multiple ethnic background
- ❑ Asian/Asian British - Indian
- ❑ Asian/Asian British - Pakistani
- ❑ Asian/Asian British – Bangladeshi
- ❑ Asian/Asian British - Chinese
- ❑ Asian/Asian British - Any other Asian background
- ❑ Black/ African/Caribbean/Black British - African

For peer review only

❑ Black/ African/Caribbean/Black British - Caribbean

❑ Black/ African/Caribbean/Black British - Any other Black/African/Caribbean background

❑ Arab

❑ Other

Q. For how many years have you worked with AAC? _______ years.

Q. What is your professional background? You may select more than one option if applicable.

❑ Occupational therapist

❑ Speech and language therapist

❑ Assistive technology specialist

❑ Clinical scientist

❑ Teacher

❑ Other

Q. If you selected Other, please specify.

____

Q. How much of your role relates to AAC? _______%.

(e.g. 1 day per week = 20%, 2 days a week = 40%, etc.)

Q. How would you characterise your role? Pick the one that best describes your role.

❑ I refer on anyone who may benefit from AAC

❑ I assess and implement AAC. I seek support from within my own team for decisions made

❑ I assess and implement AAC. I seek support from outside my own team for

❑ decisions made

❑ I assess and implement AAC. I act as a support for others in relation to AAC

❑ decision making

❑ I assess only. I provide support to others outside my team in relation to

❑ AAC decision making

❑ Other

Q. If you selected Other, please specify.

____

For peer review only

Q. Out of the list below, select the three most common diagnoses you encounter in your work.

- ❑ Autism Spectrum Disorder
- ❑ Physical disability (e.g. neuromuscular, cerebral palsy etc.)
- ❑ Dyspraxia
- ❑ Intellectual Disability/Developmental Delay
- ❑ Neurological
- ❑ Specific Speech/Language Impairment
- ❑ Syndromes
- ❑ Unknown
- ❑ Other

Q. If you selected Other, please specify.

____

Q. Who do you provide services for? (Please choose all that apply.)

- ❑ All age groups
- ❑ Preschool age
- ❑ Primary school age
- ❑ Secondary school age
- ❑ Higher education
- ❑ Further education
- ❑ Adults
- ❑ Other

Q. If you selected Other, please specify.

____

Q. What is the geographical area covered by your service? (Please choose all that apply.

- ❑ North West England
- ❑ North East England
- ❑ Yorkshire and Humber
- ❑ West Midlands
- ❑ East Midlands
- ❑ East of England

For peer review only

- ❑ South West England
- ❑ South East England
- ❑ London
- ❑ Northern Ireland
- ❑ North Wales
- ❑ South Wales
- ❑ Mid-Wales
- ❑ Southern Scotland
- ❑ Central Scotland
- ❑ Northern Scotland
- ❑ Non-UK

End of survey

Thank you for your participation in this survey.

Your responses will contribute to the results of the I-ASC project and support the development of decision making resources for use in AAC assessments.

You can follow the progress of our research project on our website, on Facebook or on Twitter.

For peer review only

Figure A 1: Example discrete choice experiment task

Child B has delayed expressive and receptive language and able to use aided AAC for a few communicative functions. Child B is only motivated to communicate through methods other than symbol communication systems. Child B is predicted to maintain current skills and abilities (plateau).

	System 1	System 2	System 3
Graphic Representation<br>Primary type of graphic symbol used	Ideographic symbols	Photo symbols	Pictographic symbols
Consistency of layout<br>Consistency of layout of symbols on pages, including when navigating through pages to select desired output.	Highly consistent layout	Somewhat consistent layout	Inconsistent layout
Vocabulary sets<br>Pre-determined vocabulary or language package provided	Commercially provided sets without language progression	Commercially provided sets with language progression	Commercially provided sets without language progression
Size of vocabulary<br>The size of the output vocabulary available within the aided AAC system.	More than 1000 vocabulary items	50-1000 vocabulary items	Up to 50 vocabulary items
Type of vocabulary organisation<br>Primary format used to organise the vocabulary within the aided AAC system	Pragmatic organisation	Semantic syntactic organisation	Visual scene display

For this child I would choose: ○ ○ ○

On a scale from 1 to 7, how good a match is your chosen device for this child? (1=very unsuitable, 7=very suitable)

1 = very unsuitable	2	3	4	5	6	7 = very suitable
○	○	○	○	○	○	○

35%

Appendix C – Final preferred model selection process

A full model with all interaction terms and two alternative specific constants implies 98 parameters, which is too many to reliably estimate given the amount of data collected and given that many interactions are expected to be of very low magnitude. Thus, a strategy was required to identify a suitable model with fewer parameters.

The first stage was estimating a series of stepwise multinomial logit (MNL) models, beginning with a model with all 98 parameters. The parameter with the highest p-value, excluding the γ_0 constant terms, was eliminated, and a model with 97 parameters was estimated. Then the parameter with the lowest p-value was excluded and a new model run, and so on in an iterative process until only the 12 γ_0 constant terms remained (one for each non-baseline system level).

The Bayesian Information Criterion (BIC) was used to select the preferred MNL model. This model was then re-estimated as a mixed logit (MIXL) model to account for participant heterogeneity. (The process did not begin by estimating a series of stepwise MIXL models due to the difficulty and greatly increased computational resources required to estimate MIXL models with a large number of parameters.) The β \coefficients on system attribute levels were assumed to be drawn from normal distributions with means given by

$$\bar{\beta}_{alc} = \gamma_{al0} + \gamma_{al} z_c$$

and variances given by

$$\sigma_{alc}^2 = (\sigma_{al0} + \sigma_{al} z_c)^2.$$

If p is the number of parameters of the preferred MNL model, then models with between $p - 3$ and $p + 3$ parameters were re-estimated as MIXL models. The BIC for each MIXL model is given in **Error! Reference source not found.**.

The MIXL model minimising the BIC was chosen as the final preferred model.

For peer review only

Table C 1: Bayesian information criteria (BIC) for estimated mixed logit models

Number of parameters	BIC
22	3502.25
23	3487.80
24	3482.30
25	3489.18
26	3493.07
27	3502.28
28	3509.34

For peer review only

Appendix D – Relative interaction attribute importance

Relative information attribute importance (RIAI) measures the amount that preferences for attributes of choice objects are impacted by a given interaction attribute associated with a choice situation relative to other interaction attributes. It may be calculated either with respect to a single choice object attribute or overall with respect to all choice object attributes.

RIAI is calculated with respect to a single choice object attribute by taking the difference between the greatest increase an interaction attribute causes to a choice object attribute's part worth utility and the greatest decrease, expressed as a percentage of the differences for all interaction attributes. Formally, the RIAI for interaction attribute i with respect to choice attribute c is

$$RIAI_{ic} = 100\left(\frac{\gamma_{ic}^{max} - \gamma_{ic}^{min}}{\sum_{j=1}^{N_I} \gamma_{jc}^{max} - \gamma_{jc}^{min}}\right)$$

where γ_{ic}^{max} and γ_{ic}^{min} are respectively the maximum and minimum coefficients for interaction attribute i with respect to choice attribute c and N_I is the number of interaction attributes. The overall RIAI for i is similarly calculated as

$$RIAI_{i} = 100\left(\frac{\gamma_{i}^{max} - \gamma_{i}^{min}}{\sum_{j=1}^{N_I} \gamma_{j}^{max} - \gamma_{j}^{min}}\right)$$

Where now γ_{i}^{max} and γ_{i}^{min} are respectively the maximum and minimum coefficients for interaction attribute i across all choice attributes.

For peer review only

BMJ Open

Finding the best fit: Examining the decision making of augmentative and alternative communication professionals in the UK using a discrete choice experiment

Journal:	*BMJ Open*
Manuscript ID	bmjopen-2019-030274.R2
Article Type:	Original research
Date Submitted by the Author:	27-Oct-2019
Complete List of Authors:	Webb, Edward; University of Leeds, Leeds Institute of Health Sciences<br>Lynch, Yvonne; Manchester Metropolitan University, Faculty of Health, Psychology and Social Care<br>Meads, David; University of Leeds, Leeds Institute of Health Sciences<br>Judge, Simon; Barnsley Hospital NHS Foundation Trust, Barnsley Assistive Technology Team<br>Randall, Nicola; Barnsley Hospital NHS Foundation Trust, Barnsley Assistive Technology Team<br>Goldbart, Juliet; Manchester Metropolitan University, Faculty of Health, Psychology and Social Care<br>Meredith, Stuart; Manchester Metropolitan University, Faculty of Health, Psychology and Social Care<br>Moulam, Liz; Manchester Metropolitan University, Faculty of Health, Psychology and Social Care<br>Hess, Stephane; University of Leeds, Choice Modelling Centre and Institute of Transport Studies<br>Murray, Janice; Manchester Metropolitan University, Faculty of Health, Psychology and Social Care
**Primary Subject Heading**:	Communication
Secondary Subject Heading:	Health economics, Health services research
Keywords:	discrete choice experiment, augmentative and alternative communication, clinical decision making

SCHOLARONE™
Manuscripts

Finding the best fit: Examining the decision making of augmentative and alternative communication professionals in the UK using a discrete choice experiment

Edward J.D. Webb[i,ii] Yvonne Lynch[iii,iv] David Meads[ii,v] Simon Judge[iii,vi,vii]

Nicola Randall[iii,vi,viii] Juliet Goldbart[iii,ix] Stuart Meredith[iii,x]

Liz Moulam[iii,xi] Stephane Hess[xii,xiii] Janice Murray[iii,xiv]

Keywords: discrete choice experiment; augmentative and alternative communication; clinical decision making

Word count: 5162

[i] Corresponding author; e.j.d.webb@leeds.ac.uk; +44 113 343 2982; Leeds Institute of Health Sciences (LIHS), Level 10 Worsley Building, Clarendon Way, Leeds, LS2 9NL, UK. OrcID: 0000-0001-7918-839X
[ii] Leeds Institute of Health Sciences, University of Leeds
[iii] Faculty of Health, Psychology and Social Care, Manchester Metropolitan University
[iv] lynchyv@tcd.ie
[v] d.meads@leeds.ac.uk
[vi] Barnsley Assistive Technology Team, Barnsley Hospital NHS Foundation Trust
[vii] Simon.judge@nhs.net
[viii] nicola.randall1@nhs.net
[ix] j.goldbart@mmu.ac.uk
[x] meredithsn@hotmail.com
[xi] lizmoulam@aol.com
[xii] Choice Modelling Centre and Institute for Transport Studies, University of Leeds
[xiii] s.hess@leeds.ac.uk
[xiv] j.murray@mmu.ac.uk

For peer review only - http://bmjopen.bmj.com/site/about/guidelines.xhtml

ABSTRACT

Objectives: Many children with varied disabilities, e.g., cerebral palsy, autism, can benefit from augmentative and alternative communication (AAC) systems. However, little is known about professionals' decision-making when recommending symbol based AAC systems for children. This study examines AAC professionals' preferences for attributes of AAC systems and how they interact with child characteristics.

Design: AAC professionals answered a discrete choice experiment (DCE) survey with AAC system and child-related attributes, where participants chose an AAC system for a child vignette.

Setting: The survey was administered online in the UK.

Participants: 155 UK-based AAC professionals were recruited between 20/10/17 and 4/3/18.

Outcomes: The study outcomes were AAC professionals' preferences as quantified using a mixed logit model, with model selection performed using a stepwise procedure and the Bayesian Information Criterion.

Results: Significant differences were observed in preferences for AAC system attributes, and large interactions were seen between child attributes included in the child vignettes, e.g., participants made more ambitious choices for children who were motivated to communicate using AAC, and predicted to progress in skills and abilities. These characteristics were perceived as relatively more important than language ability and previous AAC experience.

Conclusions: AAC professionals make trade-offs between attributes of AAC systems, and these trade-offs change depending on the characteristics of the child for whom the system is being provided.

STRENGTHS AND LIMITATIONS OF THIS STUDY

- This was the first discrete choice experiment (DCE), and only the second stated preference study in the field of augmentative and alternative communication (AAC).
- The study used unusual and innovative methodology by (1) using a Best-worst Scaling case 1 study in attribute selection; (2) having AAC system choices be made in the context of a child vignette

For peer review only - http://bmjopen.bmj.com/site/about/guidelines.xhtml

For peer review only

formed from a set of attributes; and (3) introducing a new measure termed relative interaction attribute importance to interpret results.

- Child vignettes were relatively simple, and a single vignette could represent children with very different needs.
- In some ways, the DCE task differed from how augmentative and alternative professionals make decisions in practice.

INTRODUCTION

Many people lack the ability to produce intelligible speech to meet their functional needs for a wide range of reasons, including cerebral palsy, intellectual/developmental delays and autism spectrum disorder. Even within disability types, individuals' communication related needs and abilities are extremely varied. Augmentative and alternative communication (AAC) refers to methods of supporting communication. AAC systems encompass unaided methods including signing, facial expressions, body language, as well as the use of aided systems.[1] This article focused on aided systems, also known as communication aids, which include high-tech electronic devices, such as those used by Stephen Hawking or Britain's Got Talent winner Lee Ridley, as well as low-tech systems, such as boards and communication books.

AAC can improve the lives of people with communication disabilities.[2-4] Appropriate AAC is especially important for the estimated 1 in 200 children in the UK[5-7] who require these kind of supports. Not only are their language and communication abilities still developing and their needs evolving[8-10], the systems used in childhood can potentially have impacts lasting a whole lifetime.[4]

Major advances in the AAC landscape have occurred in recent years.[11 12] These include technological innovation, for example iPads and eye-tracking, though low-tech systems may still offer the best solution in many cases.[13 14] Another development within services is a greater expectation of participation in all aspects of life for people who use AAC,[11 15-17] coupled with advocacy for the right to communicate.[14] New possibilities for AAC have been created by new communication methods such as text messaging[18], email[19] and social media.[20 21]

For peer review only - http://bmjopen.bmj.com/site/about/guidelines.xhtml

Despite the benefits AAC can offer, high rates of abandonment (30-50%) of AAC systems by children have been observed[22 23], with causes of abandonment not well understood. AAC systems can be costly (up to £10,000) and require a large amount of professional support.[24] However, when recommended appropriately and well implemented, AAC systems have been suggested to be a cost-effective use of the UK's National Health Service (NHS) resources.[25]

The process through which children receive AAC systems varies, both across and within countries.[26-28] In the UK, the context for this study, children's needs and abilities are commonly assessed by a team of AAC professionals, which may include speech and language therapists, occupational therapists and/or specialist teachers.[29 30] Final recommendations and decision-making about AAC systems are made with variable input from the child and family.

Choosing an AAC system requires consideration of many features. For example, what type of graphic symbols, e.g., photos/stylized pictures/words, to use, how many symbols are available, how they are organized, and how they are accessed.[10 31 32] The large degree of heterogeneity in the population of people who benefit from AAC, and in the systems available, means the assessment and subsequent matching of individual and system is a complex task and unique to each person.[26 28 33]

There is currently a lack of documented evidence for assessment and decision-making processes,[33-35] and what does exist is largely individual case studies.[3 25 36] AAC professionals must often make difficult and complex decisions in a complicated, heterogeneous and rapidly evolving environment, balancing the needs of an individual child, and available resources.[30 37] They must also take account of the cultural and contextual influences shaping each assessment.[13 38] While there have been studies which have highlighted some important factors in decision-making,[6 33 39] available guidelines have tended to focus on the organisational structure of services, rather than decision-making as such.[29 34 40]

The current study addressed the knowledge gaps by providing quantitative evidence about AAC professionals' decision-making using a survey method termed a discrete choice experiment (DCE). DCEs are commonly used in healthcare,[41-43] and can quantify the preferences of patients, health professionals and the public for

For peer review only - http://bmjopen.bmj.com/site/about/guidelines.xhtml

treatments, service delivery methods, policies, or other things. In this case, the goal was measuring the preferences of AAC professionals when choosing AAC systems.

This study was part of a wider project entitled *Identifying Appropriate Symbol Communication aids for children who are non-speaking: enhancing clinical decision making* (I-ASC), which examined provision of AAC systems for children in the UK. I-ASC had several components, using different research methods[30 37 44 45] to generate a body of evidence on current practice and recommendations for best practice. This has resulted in resources to aid AAC decision-making available here: https://iasc.mmu.ac.uk.

Although there is a lack of robust evidence surrounding the decision-making process, some factors in successful adoption of AAC have been identified. An AAC system is more likely to be adopted by a motivated child[22] with good support from the child's network.[27 33 44] The AAC system must also meet a child's individual needs and circumstances, which will be unique to every child.[14 22 46]

A previous study from the current research project investigated the AAC decision-making process using a Best-worst Scaling (BWS) case 1 survey.[45] This method was chosen as it could quantify which of several child and AAC system related factors (37 in total) AAC professionals considered most and least important in decision-making.

The current study sought to complement the previous work by examining fewer factors in more detail using a DCE.[43] It aimed to quantify the clinical judgements and trade-offs AAC professionals make between different attributes of AAC systems, and how those trade-offs change depending on children's characteristics, things not possible using BWS case 1. This is the first DCE carried out in AAC, and there were challenges associated with performing a DCE with a target population of AAC professionals (for details see discussion). Thus, an additional goal was to establish the feasibility of using DCEs as a research tool in AAC.

METHODS

Survey development

For peer review only - http://bmjopen.bmj.com/site/about/guidelines.xhtml

A DCE on AAC professionals' decision making

No stated preference work existed in AAC prior to the current project, and there were a large number of potential attributes with little evidence as to which to include in a DCE. A BWS case 1 study was hence performed initially and the results used to guide attribute selection for the DCE. In line with good practice and to ensure attributes were meaningful and relevant, qualitative methods were used to generate attributes.[47 48] Attributes for the BWS study were generated through focus groups and interviews with AAC professionals, people who use AAC, their families, and other stakeholders; systematic literature reviews; and input from an expert panel. For more details see section 2 of Webb et al. [45]

The BWS study produced relative importance scores for 19 child and 18 system attributes given in Appendix A. DCE attributes were selected from these during consensus discussions between authors with expertise in AAC, speech and language therapy, and health economics. The selection criteria were that attributes should: (1) form coherent and realistic descriptions of children and systems; (2) address the research aims of the wider research project, e.g., a focus on symbol communication systems; (3) include mainly attributes with high relative importance scores in the BWS study; and (4) be small in number so choice tasks would not over-burden respondents. Consensus was achieved via unstructured discussions until all authors were in agreement. This resulted in four child and five system attributes. The attributes are listed in Tables 1 and 2, together with non-specialist descriptions for the benefit of the general reader. (For a further introduction see Beukelman and Mirenda.[17])

In summary, the child attributes captured a child's language ability, experience with AAC, attitude/motivation to communicate with AAC, and whether the child is expected to regress, plateau or progress in communication ability. A total of 54 child vignettes were formed from the set of child attributes. Authors with expertise in AAC and speech and language therapy identified and removed 18 child vignettes representing unrealistic combinations, leaving 36.

AAC system attributes broadly captured the vocabulary set(s) provided by manufacturers, vocabulary size and organisation, type of graphic symbols used, and how consistent the navigational layout of words/symbols is when accessing items. It was not stated whether a system was high-tech or low-tech, although certain levels,

For peer review only - http://bmjopen.bmj.com/site/about/guidelines.xhtml

e.g., vocabulary sets with staged progression, are more common with high-tech systems. Authors with experience in AAC and speech and language therapy removed 158 unrealistic combinations from the 432 AAC systems which could be formed from the system attributes, leaving 274.

Prior experience from the BWS study suggested it would be difficult to recruit a large respondent sample, so to maximise the information captured a relatively heavy response burden of 12 choices between three systems was selected for the DCE. Participants were shown three child vignettes, referred to as Child A, B and C, and made four choices for each child vignette. An example task is shown in Appendix B.

The survey's statistical design (i.e., which levels of the AAC system attributes were presented in each question) was generated using NGene[i], with 60 choice tasks split into five blocks. The design sought to maximise D-efficiency, a measure of how much information it is possible to extract from survey responses.[49]

The survey was piloted by five AAC professionals and consequently the wording of some attributes and levels altered.

Survey administration

The DCE was administered online for ease of recruitment. Recruitment was carried out via AAC professionals' email distribution lists (the project's own list and the mailing list of the UK wide charity Communication Matters[ii]). In addition, invitations were sent via publicly available lists and websites, and the professional contacts of authors. Adverts were also placed on the project website and online media. Responses were collected between 20/10/17 and 4/3/18. Ethical approval was received from an NHS Research Ethics Committee (REC reference 6/NW/0165) and informed consent obtained from participants at the start of the survey.

Participants began by confirming they contributed towards AAC decision-making for children, and those who indicated they did not progressed directly to demographic questions that were at the end of the survey (for

[i] ©ChoiceMetrics
[ii] www.communicationmatters.org.uk

For peer review only - http://bmjopen.bmj.com/site/about/guidelines.xhtml

details, see Appendix A).[i] Three child vignettes and one survey block were randomly allocated to each participant. The order of system attributes was randomised between participants, but consistent within participants, and which systems appeared on the left, middle and right of the screen was also randomised.

Analysis

Analysis of participants' choices was grounded in random utility theory. This standard approach[50] assumed participants chose the object which maximised their utility. The utility of an object was modelled as depending partly on the object's attributes and partly random, the latter component capturing the influence of all factors not included in the model. In a given choice scenario t, participant i chose which of three AAC systems to allocate to child c. The utility to participant i of allocating AAC system $s \in \{1,2,3\}$ to child c in choice scenario t was

$$u_{isc} = \alpha_s + \beta_{ic} x_s + \varepsilon_i$$

where α_s was an alternative specific constant for AAC system s, x_s was a vector of dummy variables indicating AAC system levels, β_{ic} was a vector of coefficients which differ across participants and children, and ε_i was a random error term.

The coefficient on level l of system attribute a, β_{ialc}, depended on the characteristics of the child vignette according to

$$\beta_{ialc} = \gamma_{ial0} + \gamma_{ial} z_c$$

[i] The precise wording of the question was: *"I confirm my work involves assessing children for aided AAC systems and I contribute to the decision making in relation to the language and vocabulary organisation within AAC systems."* During testing it was revealed that some AAC professionals did not have sufficient input into the decision making process in their day-to-day practice for the DCE questions to be meaningful, e.g., occupational therapists specialising in optimising physical access to an AAC system recommended by other members of the team, and this question was designed to filter out such respondents.

For peer review only - http://bmjopen.bmj.com/site/about/guidelines.xhtml

where γ_{ial0} was a constant giving the preference for a system attribute at baseline child levels, z_c was a vector of dummy variables indicating vignette levels and γ_{ial} was a vector of coefficients, allowing for heterogeneity in relative preference for AAC system attributes depending on child characteristics.

A full model with all interaction terms included too many parameters to estimate reliably. Thus, parameters were eliminated in a stepwise process and a final preferred mixed logit model was identified using the Bayesian Information Criterion (BIC). The mixed logit model incorporated participant heterogeneity by allowing AAC system attribute parameters to be random, following a normal distribution with both means and variances depending on child characteristics. For details, see Appendix C.

Models were estimated using the CMC Choice Modelling Centre Code for R version 1.1[51] and all analysis was carried out using R version 3.3.1. Statistical significance was assessed at the 5% level after adjusting for multiple testing using Holm's sequential Bonferroni correction.[52]

Results were presented using a new measure termed *relative interaction attribute importance* (RIAI) which assessed how big an impact child attributes have on AAC professionals' decision-making. RIAI is analogous to relative attribute importance, often used to present DCE results, [53] and may be calculated either with respect to a single choice object attribute or overall with respect to all choice object attributes. For a formal definition of RIAI, see Appendix D.

Patient and public involvement

One author (SM) is an AAC user, and one (LM) is the parent of an AAC user, and both were involved in all stages of research development and delivery.

RESULTS

A total of 172 participants completed the survey, of whom 155 indicated they contributed to decision-making regarding AAC systems and answered DCE questions. Summary statistics of their demographics and professional experience are given in Table 3. Most participants were female (~90%) and white. We believe

For peer review only - http://bmjopen.bmj.com/site/about/guidelines.xhtml

this to be reasonably representative of the population of AAC professionals in the UK.[i] The mean age of DCE participants was around 40, with a range from 24 to 65, and they had on average 10 years' experience of AAC. Around 75% of DCE participants had a speech and language therapy background, with no other background reported by more than 10%. Those who did not answer DCE questions were less likely to have a speech and language therapy background (~50%), with teacher (~20%) and occupational therapist (~30%) more common.

Approximately 30% of the sample worked with all age groups, while 50-60% worked with pre-school, primary school and secondary school aged children. Participants were asked for the three most common diagnoses encountered in their work, with ~80% stating physical disability, 70% stating intellectual disability/developmental delay and 65% stating autism spectrum disorder.

Turning to DCE responses, respondents chose the left-hand option 37.6% of the time, and the central and right-hand options 33.1% and 29.2% of the time respectively, significantly different from an equal distribution (one sample Kolmogorov-Smirnov p = 0.002).

Table 4 contains the results of the final preferred model, with 24 coefficients. Figure 1 illustrates the RIAI of child attributes for each system attribute and overall. The constant terms in Table 4 give participants' preferences for AAC system allocation when shown a child vignette with all attributes at baseline levels. This baseline vignette is as follows: *"Child A/B/C has delayed expressive and receptive language and no previous AAC experience. Child A/B/C does not appear motivated to communicate through any methods and means. Child A/B/C is predicted to regress in skills and abilities (regression)."* It represents what was considered by the researchers as the most challenging profile that can be formed from the set of child attributes.

The interaction terms represent how respondents' preferences for AAC systems changed if choosing for a child vignette which differed on a given child attribute.

Vocabulary sets

---

[i] E.g., data from the Health and Care Professionals Council showed speech and language therapists in the UK were 96% female and the Higher Education Statistics Agency found speech and language therapy students in 2017/18 were 79% white. Source: Royal College of Speech and Language Therapists, personal communication.

For peer review only - http://bmjopen.bmj.com/site/about/guidelines.xhtml

For the baseline child vignette, vocabulary sets which are fixed or have staged progression were preferred to no pre-installed vocabulary. Only a single child attribute influenced preferences: Professionals were much more likely compared to the baseline to choose systems with staged progression vocabulary sets over no pre-installed set if the child vignette was predicted progress in skills and ability (odds ratio, OR 3.88) (Table 4).

Consistency of layout

For the baseline child vignette, consistent layout or an idiosyncratic layout was preferred to only having some aspects of system layout consistent for use, with no interactions with child attributes (Table 4).

Vocabulary organisation

For the baseline child vignette there was no significant preference between visual scene, taxonomic or semantic-syntactic vocabulary organisation, whilst pragmatic organisation was preferred. There were two significant interactions between vocabulary organisation and motivation. A child vignette with motivation to communicate using AAC became more likely to be allocated a system with taxonomic (OR 2.03), or semantic-syntactic (OR 2.29) organisation compared to visual scene layout (Table 4).

Size of vocabulary

For the baseline child vignette there were no significant differences in preferences between up to 50 and between 50-1000 vocabulary items, but over 1000 items were considered significantly less appropriate. A mid-size vocabulary (50-1000 items) became more preferable compared to 50 or fewer for a child vignette motivated to communicate using AAC. Over 1000 items became significantly more preferable for child vignettes with each of the following characteristics: receptive language exceeding expressive language, an ability to use a range of AAC functions, motivated to communicate using AAC and predicted to progress (Table 4). All child attributes influenced preferences for vocabulary size. As measured using RIAI, communication ability with AAC (32%) and determination and persistence (28%) were relatively more important than future skills and abilities (22%) and receptive and expressive language (17%) (Figure 1).

Graphic representation

For peer review only - http://bmjopen.bmj.com/site/about/guidelines.xhtml

For the baseline child vignette there was no preference between graphic representation using photos or pictographs, but text was less preferred than either, and idiographic symbols were even less preferred. Interactions were seen with two child attributes. Motivation to communicate using AAC increased the probability of choosing pictographic symbols (OR 3.88), idiographic symbols (OR 5.31), or text (OR 4.00) rather than photos. However, being predicted to progress made pictographic symbols less preferable (Table 4).

Overall RIAI of child attributes

Overall, future skills and abilities had the highest RIAI (38%), followed by child's determination and persistence (19%), communication ability with AAC (20%), and receptive and expressive language (12%) (Figure 1).

DISCUSSION

This DCE has revealed AAC professionals' priorities when choosing AAC systems for children, and shown that these priorities change when faced with children with different characteristics. That priorities change in this way is not unexpected, and in line with previous research showing that AAC professionals recognise the importance of matching an AAC system to an individual person's needs.[22 54] However, the current study builds on previous findings by showing the magnitude of preference changes, as for some system attributes their preferences for different levels could completely reverse depending which child vignette was shown. For example, for the baseline child vignette (see Table 4), a system with more than 1000 vocabulary items was less likely to be chosen than one with less than 50 (OR 0.395). However, for a child vignette describing a receptive-expressive language gap, the ability to use AAC for a range of functions, motivation to use AAC and predicted progression, a system with more than 1000 vocabulary items was more likely to be chosen (OR 22.5). Such flexibility is encouraging, as it is in line with one of Williams et al.'s[14] five principles for AAC application: "AAC systems must be highly individualised and appropriate to individual needs" (p.195).

For peer review only - http://bmjopen.bmj.com/site/about/guidelines.xhtml

A key finding was that the attribute of the child's determination and persistence had the greatest number of interactions with preferences and was more important in terms of RIAI than language ability or previous experience with AAC. Specifically, the attribute level motivation to communicate using AAC tended to drive participants towards what can be regarded as more ambitious choices, for example more vocabulary items. It may be that participants believed that motivated children are more likely to succeed with such AAC systems, in line with previous findings that attitude towards AAC, and valuing an AAC system are important factors in successful adoption of AAC.[22 54]

Visual scene vocabulary organisation and graphic representation using photos can both involve items/scenes from an individual's own life, and use literal, rather than abstract depictions. Both were less preferred for child vignettes motivated to communicate via AAC. Rather, participants favoured more abstract methods of organisation (taxonomic and semantic-syntactic) and graphic symbols that require more grammar (pictographs, ideographs and text). Preferences for abstract methods of organisation and symbols requiring more grammar may be interpreted as an (unfounded[55]) belief that motivated children will be better able to use more complex AAC systems. An alternative and by no means mutually exclusive interpretation is that lack of motivation requires an AAC system involving familiar cues from their everyday environment.

Previous studies have also studied how AAC professionals choose graphic symbols for children.[56] For example, Thistle and Wilkinson[33] found that cognitive abilities are an important factor, as did Dada et al.[46] The advantage of a DCE was that the precise interactions between child characteristics and symbol type have been enumerated, showing, e.g., which children were more likely to be given AAC systems with photos, and which were more likely to be given systems with text. AAC system preferences did not significantly differ between child vignettes where their skills and abilities were predicted to regress or plateau. However, if a child was predicted to progress, this had a large impact on professional decision-making, with anticipated future skills and abilities ranked as the highest attribute in terms of RIAI. As with motivation, skills and abilities led to more ambitious choices, with more vocabulary items preferred and pictographs depreciated compared to ideographs and text. Such ambitious choices could reflect participants wishing to provide AAC

For peer review only - http://bmjopen.bmj.com/site/about/guidelines.xhtml

systems that would fulfil the future needs of children who are anticipated to progress, given the large investment that goes into learning a new AAC system.[57-59] With plateau or regression this was less of a concern.

Photos were still the most preferred aided communication mode unless a child vignette featured both predicted progress *and* motivation to communicate via AAC. This preference for photos possibly indicates that photos remain a good starting point for a child who is not engaged, regardless of prognosis, and may reflect recommendations that recognise the need to reduce the learning demands of AAC systems for some children.[12 60]

Despite unwelcome rates of abandonment, AAC professionals had high expectations of motivated children who were expected to progress, even if their receptive and expressive language were both delayed and they had no previous AAC experience. It has previously been noted that people who use AAC experience an asymmetry between the language they receive and the language they are able to express.[61] One interpretation is that participants wished to minimise asymmetries by choosing text as the expressive output for children they believed could cope with it. These ambitious choices are also encouraging given the greatly increased aspirations for effective societal participation of AAC users.[11 15 16] It is also in line with official guidance[62] and one of Williams et al.'s[14] five principles for AAC: "AAC must support full participation in all aspects of 21st century life" (p.195).

For many of the child vignettes there were non-linear preferences for vocabulary size. Offering between 50 and 1000 items was considered better than 50 or fewer for all child vignettes, although the difference was not always significant. Systems with fewer than 50 items being depreciated may indicate that participants were mindful of limiting children's potential for expression, even for children with lower cognitive ability and poor prognosis.

Findings suggest that respondents preferred levels of AAC systems that require personalisation, e.g., pragmatic vocabulary organisation or an idiosyncratic layout, in line with previous findings that personalisation is important in successful AAC adoption.[28] A preference for personalisation indicates that it

For peer review only - http://bmjopen.bmj.com/site/about/guidelines.xhtml

is not possible to achieve the goal of AAC systems being closely tailored to individuals' needs[14 63] with "off-the-shelf" AAC systems: in other words, some personalisation is always necessary.[64 65]

Pre-installed vocabulary sets were always preferred over no pre-provided set, in line with other studies showing that selecting core vocabulary was an important part of AAC professionals' decision-making process.[33 37]

Comparing the DCE results with the previous BWS Case 1 study,[45] some similarities may be observed. For example, graphic representation was the lowest ranked attribute in terms of importance in the BWS to be included in the DCE. In concordance with this finding, when the relative importance of AAC system attributes was calculated for each child vignette in the DCE, graphic representation was never the most important attribute. The relative lack of importance ascribed to graphic representation raises debate about the fundamental components of language construction through aided means and suggests much further research is required.

Many differences to the BWS findings can also be seen. Language abilities were the most important child attribute in the BWS, yet its RIAI in the DCE was below predicted future abilities, ranked sixth in the BWS. However, differences do not necessarily imply contradiction, as the two methodologies did not measure the same thing. The BWS measured the importance of AAC system attributes over the case mix AAC professionals encounter in practice, whereas for the DCE respondents were presented with a specific child vignette.

Receptive and expressive language had the lowest RIAI overall, with only a single interaction term in the final model. This contrasts with some previous findings that a child's language abilities play a large role in selecting an appropriate AAC System.[13 28 30 37] One possible explanation is that the aspects of language ability which were most relevant were captured in this study by other child attributes, but this remains a question to be addressed by future research.

For peer review only

The current study has demonstrated the feasibility of conducting a DCE with a target population of AAC professionals. The demonstration of feasibility is noteworthy given the relative rarity of DCEs studying health professionals' decision-making. For example in a systematic review[41] of DCEs in health published between 2013 and 2017, only 13% included a sample of health professionals. In addition, there were particular challenges associated with performing a DCE with AAC professionals. The target population in the UK is small, meaning it was uncertain that sufficient participants for a successful study could be recruited. There were also concerns that participants might not find the DCE format acceptable, as they might have rejected having to make compromises between AAC system attributes in the context of providing a system for a child. Yet despite informal feedback that some respondents found the tasks uncomfortable, many were still willing to complete them. Finally, as interactions between child characteristics and AAC systems are so important, it was necessary to present hypothetical child vignettes, making tasks more complicated than in a typical DCE.

Despite these potential pitfalls, the DCE was successfully carried out, and having demonstrated the feasibility of the method in this area, further DCE studies should be considered in future.

Limitations

The current study has several limitations. The sample size was relatively small (155 participants, compared to a median for healthcare DCEs of 401[41]). However, many studies exist with smaller sample sizes (e.g., Spinks et al.[66] with 35), and it was possible to estimate robust statistical models. Furthermore, it would have been difficult to collect a larger sample, as 155 participants represented a large proportion of the population of AAC professionals in the UK working with children, which was estimated at around 800.[i]

The DCE task may not match how UK AAC professionals make decisions in practice. Typically, many participants have the opportunity to work with families and children, as well as part of an AAC team, which could include diverse areas of clinical and personal expertise. Teams also generally make recommendations, rather than unilaterally choosing a system. However, there is evidence that AAC professionals compare the

[i] Communication Matters, personal correspondence

For peer review only - http://bmjopen.bmj.com/site/about/guidelines.xhtml

attributes of AAC systems in everyday practice,[13] and that they make trade-offs between system attributes,[37] akin to DCE tasks. In addition, it is still useful to study the individual decision-making of AAC professionals. Lynch et al.[30] reported that a wide variety of team structures are used, and the mode of service delivery can have an influence on outcomes. Gathering evidence on individual-level decision-making can thus inform an assessment of how different ways of organising services influence decisions.

The DCE tasks presented one-off static decisions made by a single individual. In reality the decision-making environment is dynamic, with children developing over time, and often having two or more devices over the course of their childhood. These differences are a limiting factor in the external validity of results.

Attributes and levels use a mixture of speech and language therapy terms, e.g., receptive and expressive language, and more AAC specific language, e.g., staged vocabulary progression. Mixing these terms may have made it more difficult for respondents from any one professional speciality to interpret all of them.[44] However, this issue is not limited to the current study, but reflects an ongoing struggle in AAC to establish a common language.[44] In addition, respondents may have been unfamiliar with the generic term ideographic symbols, since only a single commercial set of ideographic symbols is in popular use in the UK (Minspeak[i]).

Respondents were more likely to choose AAC systems on the left of the screen and less likely to choose ones on the right. However, the risk of bias was mitigated by allowing for alternative specific constants and randomising the position in which AAC systems were presented.

Compared to the real children AAC professionals encounter, the child vignettes were simple, and lacked information which influences decision-making, such as the child's preferences[33] and contextual factors.[30] However, this is an inherent limitation of the DCE methodology, and vignettes with a greater number of attributes and levels would have made decisions overly burdensome, and therefore were not included. Significant interactions between AAC systems and child attributes implied that the vignettes were meaningful enough that respondents changed their preferences in response to them, often dramatically.

[i] © Semantic Compaction Systems, Inc.

For peer review only - http://bmjopen.bmj.com/site/about/guidelines.xhtml

For a given child vignette, it was only possible to determine relative preferences for system attributes, rather than absolute preferences. Consequently, it is not possible to tell how suitable a given system is for a given child vignette, which is important as some presented a challenging profile for which it may be hard to find a suitable AAC system.

CONCLUSION

A lack of rigorous evidence on how to best assess and provide AAC systems for children has previously been identified,[25 34 44] as well as a gap between research and current practice.[11] In the light of this, the current study's results are encouraging, as it shows AAC professionals following best practice in many areas, for example ensuring AAC systems suit individual needs, and having high expectations for many children.

However, there is still demand from AAC professionals for better support in decision-making,[33 37] and undoubtedly current practice could be improved. The results of the current study, together with evidence from the wider research project, have been used to create a heuristic and suite of resources, available at https://iasc.mmu.ac.uk. It is hoped these resources will aid AAC professionals in their clinical practice and help them provide the best possible service for children.

Acknowledgements: Thank you to Muireann McCleary and the Speech and Language Therapy team at the Central Remedial Clinic, Dublin, who piloted and gave feedback on the survey, and to participants who responded to the survey. Thanks to Mark Jayes (Manchester Metropolitan University) and Berenice Napier (Royal College of Speech and Language Therapists) for assistance in finding demographic statistics on speech and language therapists in the UK.

Funding: This independent research was funded by the National Institute for Health Research, UK (Health Services & Delivery Research Project: 14/70/153 - Identifying appropriate symbol communication aids for children who are non-speaking: enhancing clinical decision-making). The views expressed in this article are those of the authors and not necessarily those of the NHS, the National Institute for Health Research or the Department of Health.

For peer review only - http://bmjopen.bmj.com/site/about/guidelines.xhtml

Stephane Hess acknowledges additional support by the European Research Council through the consolidator grant 615596-DECISIONS.

Competing interests: The authors have no competing interests to declare.

Author statement: All authors conceived the study and defined the study aims. EW, DM, YL, NR, SJ, JG, SM, LM and JM developed attributes and levels. EW, DM and SH constructed the survey statistical design. EW and DM collected data. EW conducted statistical analysis. EW, YL, NR, SJ, JG, SM, LM and JM interpreted findings. EW wrote the manuscript first draft. All authors contributed to and approved the final manuscript.

Ethical approval: Ethical approval was received for the study from an NHS Research Ethics Committee (REC reference 6/NW/0165) and informed consent was obtained from participants at the start of the survey.

Data availability: Survey data is not publicly available as respondent consent was not obtained for this. However, it is available on request to the corresponding author or to Leeds Institute of Health Sciences if a formal data sharing agreement is entered into.

For peer review only

REFERENCES

1. Murray J, Goldbart J. Augmentative and alternative communication: a review of current issues. *Paediatrics and child health* 2009;19(10):464-68.

2. Schlosser RW, Wendt O. Effects of augmentative and alternative communication intervention on speech production in children with autism: A systematic review. *American Journal of Speech-Language Pathology* 2008;17(3):212-30.

3. Millar DC, Light JC, Schlosser RW. The impact of augmentative and alternative communication intervention on the speech production of individuals with developmental disabilities: A research review. *Journal of Speech, Language, and Hearing Research* 2006;49(2):248-64.

4. Lund SK, Light J. Long-term outcomes for individuals who use augmentative and alternative communication: Part I–What is a "good" outcome? *Augmentative and Alternative Communication* 2006;22(4):284-99.

5. Gross J. Augmentative and alternative communication: a report on provision for children and young people in England: Office of the Communication Champion, 2010.

6. Enderby P, Judge S, Creer S, et al. Examining the Need for and Provision of AAC Methods in the UK. *Advances in Clinical Neuroscience & Rehabilitation* 2013;13:20-23.

7. Judge S, Enderby P, Creer S, et al. Provision of powered communication aids in the United Kingdom. *Augmentative and Alternative Communication* 2017;33(3):181-87.

8. Hajjar DJ, McCarthy JW, Benigno JP, et al. "You Get More Than You Give": Experiences of Community Partners in Facilitating Active Recreation with Individuals who have Complex Communication Needs. *Augmentative and Alternative Communication* 2016;32(2):131-42.

9. Ryan SE, Shepherd T, Renzoni AM, et al. Towards Advancing Knowledge Translation of AAC Outcomes Research for Children and Youth with Complex Communication Needs. *Augmentative and Alternative Communication* 2015;31(2):137-47.

For peer review only - http://bmjopen.bmj.com/site/about/guidelines.xhtml

10. Dada S, Alant E. The effect of aided language stimulation on vocabulary acquisition in children with little or no functional speech. *American Journal of Speech-Language Pathology* 2009;18(1):50-64. doi: 10.1044/1058-0360(2008/07-0018)

11. Light J, McNaughton D, Beukelman D, et al. Challenges and opportunities in augmentative and alternative communication: Research and technology development to enhance communication and participation for individuals with complex communication needs. *Augmentative and Alternative Communication* 2019;35(1):1-12.

12. Light J, McNaughton D, Caron JJA, et al. New and emerging AAC technology supports for children with complex communication needs and their communication partners: State of the science and future research directions. *Augmentative and Alternative Communication* 2019;35(1):26-41.

13. Lund SK, Quach W, Weissling K, et al. Assessment with children who need augmentative and alternative communication (AAC): Clinical decisions of AAC specialists. *Language, Speech, and нearing Services in Schools* 2017;48(1):56-68.

14. Williams MB, Krezman C, McNaughton DJA, et al. "Reach for the stars": Five principles for the next 25 years of AAC. *Augmentative and Alternative Communication* 2008;24(3):194-206.

15. Calculator SNE. Augmentative and alternative communication (AAC) and inclusive education for students with the most severe disabilities. *International Journal of Inclusive Education* 2009;13(1):93-113.

16. Isakson CL, Burghstahler S, Arnold AJATO, et al. AAC, Employment, and Independent Living: A Success Story. *Assistive Technology Outcomes and Benefits* 2006;3(1):67-79.

17. Beukelman DR, Mirenda P. Augmentative and alternative communication: Supporting children and adults with complex communication needs: Paul H. Brookes Pub. 2013.

18. Williams M, Beukelman D, Ullman C. AAC text messaging. *Perspectives on Augmentative and Alternative Communication* 2012;21(2):56-59.

19. Sundqvist A, Rönnberg JJA, communication a. A qualitative analysis of email interactions of children who use augmentative and alternative communication. *Augmentative and Alternative Communication* 2010;26(4):255-66.

For peer review only - http://bmjopen.bmj.com/site/about/guidelines.xhtml

20. Hemsley B, Murray J. Distance and proximity: research on social media connections in the field of communication disability. *Disability and Rehabilitation* 2015; 37:17, 1509-1510.

21. Hynan A, Goldbart J, Murray J. A grounded theory of Internet and social media use by young people who use augmentative and alternative communication (AAC). *Disability and Rehabilitation* 2015;37(17):1559-75.

22. Johnson JM, Inglebret E, Jones C, et al. Perspectives of speech language pathologists regarding success versus abandonment of AAC. *Augmentative and Alternative Communication* 2006;22(2):85-99.

23. Moorcroft A, Scarinci N, Meyer C. A systematic review of the barriers and facilitators to the provision and use of low-tech and unaided AAC systems for people with complex communication needs and their families. *Disability and Rehabilitation: Assistive Technology* 2018:1-22.

24. Reddington J. The Domesday dataset: Linked open data in disability studies. *Journal of Intellectual Disabilities* 2013;17(2):107-21.

25. Munton T. Augmentative and Alternative Communication (AAC) support in Scotland: A review of the research literature and cost benefit analyses: NHS Education for Scotland, 2013.

26. Binger C, Ball L, Dietz A, et al. Personnel roles in the AAC assessment process. *Augmentative and Alternative Communication* 2012;28(4):278-88.

27. Lindsay S. Perceptions of health care workers prescribing augmentative and alternative communication devices to children. *Disability and Rehabilitation: Assistive Technology* 2010;5(3):209-22.

28. Dietz A, Quach W, Lund SK, et al. AAC assessment and clinical-decision making: The impact of experience. *Augmentative and Alternative Communication* 2012;28(3):148-59.

29. Guidance for commissioning AAC services and equipment: NHS England, 2016.

30. Lynch Y, Murray J, Moulam L, et al. Decision-making in communication aid recommendations in the UK: cultural and contextual influencers. *Augmentative and Alternative Communication* 2019:180-192.

31. Binger C, Light J. The effect of aided AAC modeling on the expression of multi-symbol messages by preschoolers who use AAC. *Augmentative and Alternative Communication* 2007;23(1):30-43.

For peer review only

32. Binger C, Light J. The morphology and syntax of individuals who use AAC: Research review and implications for effective practice. *Augmentative and Alternative Communication* 2008;24(2):123-38.

33. Thistle JJ, Wilkinson KM. Building evidence-based practice in AAC display design for young children: Current practices and future directions. *Augmentative and Alternative Communication* 2015;31(2):124-36.

34. Resource manual for commissioning and planning services for SLCN: Royal College of Speech and Language Therapists, 2009.

35. Batorowicz B, Shepherd TA. Teamwork in AAC: Examining clinical perceptions. *Augmentative and Alternative Communication* 2011;27(1):16-25.

36. Bryen DN, Chung Y, Lever SJPoA, et al. What you might not find in a typical transition plan! Some important lessons from adults who rely on augmentative and alternative communication. *Perspectives on Augmentative and Alternative Communication* 2010;19(2):32-40.

37. Murray J, Lynch Y, Meredith S, et al. Professionals' decision-making in recommending communication aids in the UK: competing considerations. *Augmentative and Alternative Communication* 2019:167-179.

38. Baxter S, Enderby P, Evans P, et al. Barriers and facilitators to the use of high-technology augmentative and alternative communication devices: a systematic review and qualitative synthesis. *International Journal of Language & Communication Disorders* 2012;47(2):115-29. doi: 10.1111/j.1460-6984.2011.00090.x

39. Geytenbeek JJ, Vermeulen RJ, Becher JG, et al. Comprehension of spoken language in non-speaking children with severe cerebral palsy: an explorative study on associations with motor type and disabilities. *Developmental Medicine & Child Neurology* 2015;57(3):294-300.

40. Choi BC, Pak AW. Multidisciplinarity, interdisciplinarity and transdisciplinarity in health research, services, education and policy: 1. Definitions, objectives, and evidence of effectiveness. *Clinical and Investigative Medicine* 2006;29(6):351.

For peer review only - http://bmjopen.bmj.com/site/about/guidelines.xhtml

41. Soekhai V, de Bekker-Grob EW, Ellis AR, et al. Discrete choice experiments in health economics: past, present and future. *PharmacoEconomics* 2019;37(2):201-26.

42. Clark MD, Determann D, Petrou S, et al. Discrete Choice Experiments in Health Economics: A Review of the Literature. *PharmacoEconomics* 2014;32(9):883-902. doi: 10.1007/s40273-014-0170-x

43. Ryan M. Discrete choice experiments in health care. *BMJ* 2004;328:360.

44. Judge S, Randall N, Goldbart J, et al. The language and communication attributes of graphic symbol communication aids–a systematic review and narrative synthesis. *Disability and Rehabilitation: Assistive Technology* 019:1-11.

45. Webb EJ, Meads D, Lynch Y, et al. What's important in AAC decision making for children? Evidence from a best-worst scaling survey. *Augmentative and Alternative Communication* 2019· 35(2):80-94.

46. Dada S, Murphy Y, Tönsing KJA, et al. Augmentative and alternative communication practices: A descriptive study of the perceptions of South African speech-language therapists. *Augmentative and Alternative Communication* 2017;33(4):189-200.

47. Coast J, Al-Janabi H, Sutton EJ, et al. Using qualitative methods for attribute development for discrete choice experiments: issues and recommendations. *Health Economics* 2012;21(6):730-41. doi: 10.1002/hec.1739

48. Kløjgaard ME, Bech M, Søgaard R. Designing a stated choice experiment: the value of a qualitative process. *Journal of Choice Modelling* 2012;5(2):1-18.

49. Kuhfeld WF, Tobias RD, Garratt M. Efficient experimental design with marketing research applications. *Journal of Marketing Research* 1994;31(4):545-57.

50. Louviere JJ, Hensher DA, Swait JD. Stated choice methods: analysis and applications: Cambridge University Press 2000.

51. CMC. CMC choice modelling code for R: Choice Modelling Centre, University of Leeds, 2017.

52. Holm S. A simple sequentially rejective multiple test procedure. *Scandinavian Journal of Statistics* 1979;6(2):65-70.

For peer review only - http://bmjopen.bmj.com/site/about/guidelines.xhtml

53. Hauber AB, González JM, Groothuis-Oudshoorn CG, et al. Statistical methods for the analysis of discrete choice experiments: a report of the ISPOR Conjoint Analysis Good Research Practices Task Force. *Value in Health* 2016;19(4):300-15.

54. Light J, McNaughton D. Communicative competence for individuals who require augmentative and alternative communication: A new definition for a new era of communication? Augmentative and Alternative Communication, 2014;30:1, 1-18.

55. von Tetzchner S. Introduction to the special issue on aided language processes, development, and use: an international perspective. *Augmentative and Alternative Communication* 2018;34(1):1-15.

56. Lynch Y, McCleary M, Smith M, et al. Instructional strategies used in direct AAC interventions with children to support graphic symbol learning: A systematic review. *Child Language Teaching and Therapy* 2018;34(1):23-36.

57. Getting the literacy and language skills needed for employment: Teaching is the solution. Proceedings of the Eighth Pittsburgh Employment Conference for Augmented Communicators; 2001. Shout Press Pittsburgh, PA.

58. Rackensperger T, Krezman C, Mcnaughton D, et al. "When I first got it, I wanted to throw it off a cliff": The challenges and benefits of learning AAC technologies as described by adults who use AAC. *Augmentative and Alternative Communication* 2005;21(3):165-86.

59. Bailey RL, Parette, H Jr, Stoner JB, et al. Family members' perceptions of augmentative and alternative communication device use. *Language, Speech, and Hearing Services in Schools* 2006

60. Light J, Wilkinson KM, Thiessen A, et al. Designing effective AAC displays for individuals with developmental or acquired disabilities: State of the science and future research directions. *Augmentative and Alternative Communication* 2019;35(1):42-55.

61. Smith MM, Grove NCJCcfiwuAFrtep. Asymmetry in input and output for individuals who use AAC. *Communicative competence for individuals who use AAC: From research to effective practice* 2003:163-95.

62. SEND Code of Practice 0–25 Years London: Department for Education, Department of Health, 2015.

For peer review only - http://bmjopen.bmj.com/site/about/guidelines.xhtml

63. Light J, McNaughton DJA, Communication A. Putting people first: Re-thinking the role of technology in augmentative and alternative communication intervention. *Augmentative and Alternative Communication* 2013;29(4):299-309.

64. King G, Batorowicz B, Shepherd T, et al. Expertise in research-informed clinical decision making: Working effectively with families of children with little or no functional speech. *Evidence-Based Communication Assessment and Intervention* 2008;2(2):106-16.

65. Parette P, VanBiervliet A, Hourcade J. Family-centered decision making in assistive technology. *Journal of Special Education Technology* 1999;15(1):45-55.

66. Spinks J, Janda M, Soyer HP, et al. Consumer preferences for teledermoscopy screening to detect melanoma early. *Journal of Telemedicine and Telecare* 2016;22(1):39-46.

For peer review only

For peer review only - http://bmjopen.bmj.com/site/about/guidelines.xhtml

Table 1: Child attributes and levels including brief descriptions

Child attributes and levels	Description†
**Receptive and expressive language (1)**	Child's ability without AAC to understand communication from (receptive) and communicate with others (expressive)
*Delayed	Both receptive and expressive abilities below expectation given child's age
Receptive language exceeding expressive language	Ability to understand communication from others greater than ability to communicate with others
**Communication ability with AAC (3)**	How well a child can communicate when using AAC
*No previous AAC experience	Has never communicated using AAC before
Able to use AAC for a few communicative functions	Can use AAC for some basic functions, e.g. simple requests
Able to use AAC for a range of communicative functions	Can use AAC for more complex tasks, e.g. constructing sentences
**Child's determination and persistence (4)**	Attitude of child towards communication and using AAC
*Does not appear motivated to communicate through any methods and means	Child is not inclined to develop communication skills
Motivated to communicate through symbol communication systems	Child has demonstrated motivation and willingness to use AAC
Only motivated to communicate through methods other than symbol communication	Child may be motivated to communicate, but is not inclined to use AAC
**Predicted future skills and abilities (6)**	Professional assessment of how child's communication abilities will develop
*Regression	Abilities projected to become worse in future, e.g. due to a degenerative condition such as Rett syndrome
Plateau	Abilities will not change significantly in future, e.g. a child aged 16-17
Progression	Communication abilities will develop in future

Note: * indicates baseline level; numbers in parentheses indicate attributes' rank in relative importance from Webb et al. [45] †Descriptions are not intended as rigorous definitions of AAC terminology, but as a rough guide for the non-AAC specialist reader.

For peer review only - http://bmjopen.bmj.com/site/about/guidelines.xhtml

Table 2: AAC System attributes and levels, including brief descriptions

AAC System attributes and levels	Description†
**Vocabulary sets (1)**	Words and/or symbols pre-provided with system "out of the box", e.g. as part of a software package for a high-tech system
*No vocabulary set	AAC practitioners/child's support network provides all vocabulary content
Fixed vocabulary set	A single fixed set of vocabulary which may be customised
Vocabulary set with staged progression	A series of vocabulary sets with pre-determined progression through them that simulate language development. E.g. an initial set including just basic words, with subsequent sets introducing more grammatical structure. May be customised.
**Consistency of layout (2)**	How consistent positions of words/symbols are in system interface, and how consistent navigation to find different symbols is
*Consistency of some aspects of layout	Words/symbols in multiple categories appear in different positions across categories, but always in the same place in a given category
Consistency of all aspects of layout	All/nearly all words/symbols always appear in same position in interface
Idiosyncratic layout	Layout that has been personalised for an individual child
**Type of vocabulary organisation (5)**	How words/symbols are organised within the system
*Visual scene	Interface shows photos, most likely of scenes familiar to the child, with areas of it highlighted to represent words
Taxonomic	Words/symbols organised according to subject, analogous to non-fiction books in a library
Semantic-syntactic	Words/symbols organised according to sentence structure, e.g. verbs, nouns, adjectives
Pragmatic	Words/symbols organised around function in language rather than grammar, e.g. request, mood
**Size of vocabulary (7)**	How many words/symbols system can output
*Up to 50 vocabulary items	Implies only simple communication functions possible
50-1000 vocabulary items	Implies combining words/symbols to create grammatical structures
More than 1000 vocabulary items	Does not imply more complex communication than 50-1000 items, but means a greater load on child's memory
**Graphic representation (12)**	Type of symbols used by system
*Photos	Photographs, possibly of items or environments personal to the child
Pictographic symbol set	Non-photorealist pictures with specific meanings attached. May be accompanied by text
Ideographic symbol system (with rules or encoding)	Stylised symbols combined with fixed rules and grammar analogous to Chinese/Japanese characters, e.g. Minspeak
Text	Text unaccompanied by other symbols

Note: * indicates baseline level; numbers in parentheses indicate attributes' rank in relative importance from prior BWS study (reported in Webb et al. [45]). †Descriptions are not intended as rigorous definitions of AAC terminology, but as a rough guide for the non-AAC specialist reader.

For peer review only

For peer review only - http://bmjopen.bmj.com/site/about/guidelines.xhtml

Table 3: Demographics and professional experience of participants

		mean	s.e
Age (years)		40.8	11
Experience (years)		11.4	9.2
% of role relating to AAC		53.7	34.3
		N	%
Gender	Female	155	90.1
	Male	10	5.81
	Prefer not to say	7	4.07
Ethnicity	White - English/Welsh/Scottish/Northern Irish/British	149	86.6
	White – other	12	6.98
	Other	6	3.49
	White – Irish	5	2.91
Professional background	Speech and language therapist	125	72.7
	Occupational therapist	16	9.3
	Teacher	14	8.14
	Other	12	6.98
	Assistive technology specialist	5	2.91
	Clinical scientist	5	2.91
Age groups worked with	Primary school age	99	57.6
	Secondary school age	94	54.7
	Pre-school age	85	49.4
	All age groups	56	32.6
	Higher education	30	17.4
	Further education	21	12.2
	Other	12	6.98
	Adults	10	5.81
Among most common three diagnoses seen in practice	Physical disability (e.g. neuromuscular, cerebral palsy etc.)	140	81.4
	Intellectual Disability/Developmental Delay	118	68.6
	Autism spectrum disorder	113	65.7
	Syndromes	61	35.5
	Neurological	45	26.2
	Specific Speech/Language Impairment	22	12.8
	Dyspraxia	14	8.14

Note. For some questions, participants could select more than one response, thus some percentages do not sum to 100%

For peer review only

For peer review only - http://bmjopen.bmj.com/site/about/guidelines.xhtml

Table 4: Parameter means and standard deviations for final mixed logit model. * indicates significance at the 5% level corrected using Holm's sequential Bonferroni [52], s.e. = standard error

AAC system attribute		Child attribute	Parameter mean	s.e.	σ	s.e.
Vocabulary sets (baseline none)	Fixed	Constant	0.283*	0.0966	0.131	0.258
	Staged progression	Constant	0.364*	0.141	0.941*	0.206
		Predicted to progress	1.36*	0.221	-1.09*	0.343
Consistency of layout (baseline some aspects)	Consistency of all aspects	Constant	0.892*	0.121	0.15	0.126
	Idiosyncratic layout	Constant	1.46*	0.14	0.757*	0.134
Type of vocabulary organisation (baseline visual scene)	Taxonomic	Constant	0.0629	0.165	0.383	0.257
		Motivated to communicate through symbol communication systems	0.707*	0.206	-0.563	0.295
	Semantic-syntactic	Constant	-0.178	0.166	0.549	0.234
		Motivated to communicate through symbol communication systems	0.826*	0.197	-0.112	0.296
	Pragmatic	Constant	0.443*	0.123	0.723*	0.152
Size of vocabulary (baseline 50 items)	50-1000 items	Constant	0.131	0.143	0.43	0.166
		Motivated to communicate through symbol communication systems	1.01*	0.232	-0.731	0.329
	More than 1000 items	Constant	-0.929*	0.213	1.02*	0.33
		Receptive language exceeding expressive language	0.692*	0.186	0.489	0.367
		Able to use AAC for a range of communicative functions	1.14*	0.319	-0.419	0.762
		Motivated to communicate through symbol communication systems	1.31*	0.272	-0.751	0.556
		Predicted to progress	0.902*	0.233	0.981	0.657
Graphic representation (baseline photos)	Pictographic symbol set	Constant	-0.41	0.183	0.0722	0.248
		Motivated to communicate through symbol communication systems	1.36*	0.24	-0.363	0.428
		Predicted to progress	-0.814*	0.217	1.12	0.385
	Ideographic symbol system	Constant	-1.25*	0.207	0.823*	0.216
		Motivated to communicate through symbol communication systems	1.67*	0.268	0.069	0.297
	Text	Constant	-0.709*	0.159	0.615*	0.204
		Motivated to communicate through symbol communication systems	1.39*	0.231	-1.12*	0.282

Note. Constants give preferences when choosing for the baseline child vignette: *"Child A/B/C has delayed expressive and receptive language and no previous AAC experience. Child A/B/C does not appear motivated to communicate through any methods and means. Child A/B/C is predicted to regress in skills and abilities (regression)."*
σ indicates standard deviation. Parameter variance for level l of AAC system attribute a when choosing for child c is given by $\sigma^2_{alc} = (\sigma_{al0} + \sigma_{al} Z_c)^2$.

For peer review only

For peer review only - http://bmjopen.bmj.com/site/about/guidelines.xhtml

Figure 1: Relative interaction attribute importance for each AAC system attribute and averaged over all attributes. Note that consistency of layout is omitted as there are no interactions with child attributes. Error bars show 95% confidence intervals.

For peer review only

For peer review only - http://bmjopen.bmj.com/site/about/guidelines.xhtml

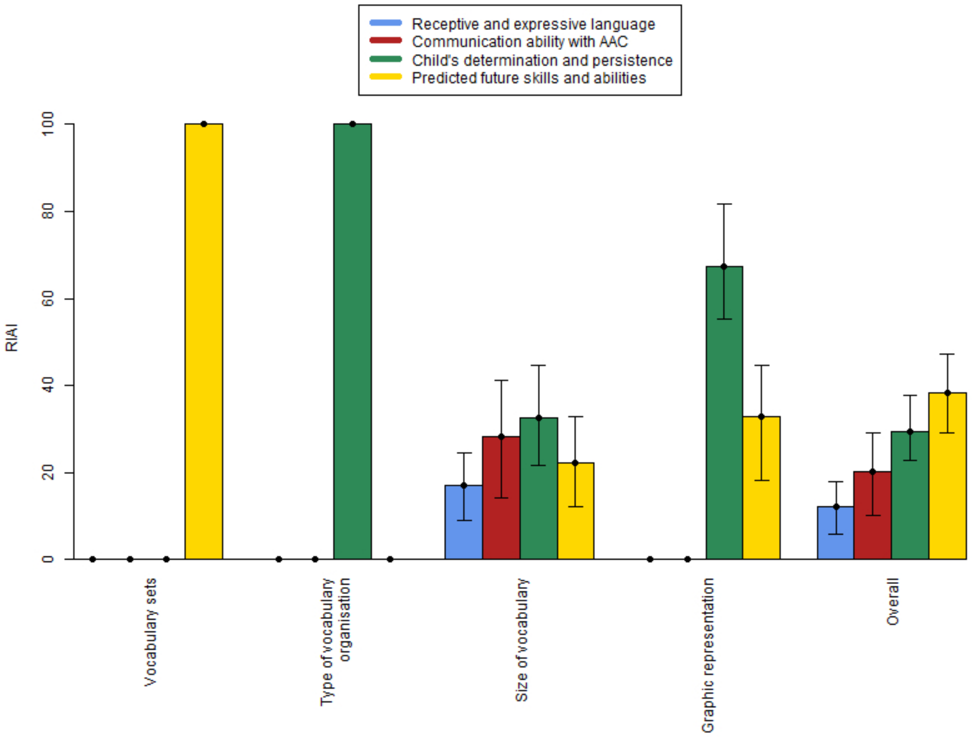

Relative interaction attribute importance for each AAC system attribute and averaged over all attributes. Note that consistency of layout is omitted as there are no interactions with child attributes. Error bars show 95% confidence intervals.

Appendix A – Attributes from best-worst scaling case 1 study

Table B 1: Attributes used in best-worst scaling case 1 survey in Webb et al. [23] and rank in terms of relative importance score

Child attribute	Rank
*Child's receptive and expressive language abilities	1
Support for AAC from communication partners	2
*Communication ability with aided AAC	3
*Child's determination and persistence	4
Physical abilities for access	5
*Predicted future needs and abilities	6
Level of learning ability	7
Insight into own communicative skills	8
Attention level	9
Access to professional AAC support	10
Speech skills and intelligibility	11
Functional visual skills	12
History of aided AAC use	13
Presence of additional diagnoses	14
Level of fatigue	15
Literacy ability	16
Educational stage	17
Primary diagnosis	18
Mobility	19
AAC system attributes	Rank
*Vocabulary or language package(s)	1
*Consistency of layout and navigation	2
Ease of customization	3
Durability and reliability	4
*Type of vocabulary organization	5
Number of key presses required to generate symbol or text output	6
*Size of output vocabulary	7
Range of access methods	8
Number of cells per page	9
Portability	10
*Graphic representation	11
Battery life	12
Supplier support	13
Ease of mounting on a range of equipment	14
Cost	15
Additional assistive technology functions	16
Voice	17
Appearance	18

Note. Asterisk indicates attribute included in discrete choice experiment.

For peer review only

Appendix B – Example survey

Note: the AAC system levels and child vignettes shown here are for illustrative purposes and do not represent the statistical design used in the full survey.

Instructions

Thank you for taking part in this survey.

It aims to identify what factors clinicians think are important when making decisions about aided AAC systems for children with communication difficulties.

You will be asked a series of questions. Each one has the same format. A brief description of a child will be given, along with three possible choices of aided AAC systems.

The three AAC systems are described in terms of five characteristics (the systems are identical apart from changes to these five characteristics):-

1. **Vocabulary sets**: Pre-determined vocabulary or language package provided, which can be:-
   - No commercially provided sets
   - Commercially provided sets without language progression
   - Commercially provided sets with language progression
2. **Size of vocabulary**: The size of the output vocabulary available within the aided AAC system, which can be:-
   - Up to 50 vocabulary items
   - 50-1000 vocabulary items
   - More than 1000 vocabulary items
3. **Type of vocabulary organisation**: Primary format used to organise the vocabulary within the aided AAC system, which can be:-
   - Visual scene display
   - Semantic organisation
   - Semantic syntactic organisation
   - Pragmatic organisation

For peer review only

4. **Graphic Representation**: Primary type of graphic symbol used, which can be:-
   - Photo symbols (i.e. a photo symbol set without rules or encoding)
   - Pictographic symbols (i.e. a graphic symbol set without rules or encoding)
   - Ideographic symbols (i.e. a symbol system with rules or encoding)
   - Graphic symbols with text (i.e. a system with either pictographic or ideographic symbols that incorporates an alphabet for generating text)
5. **Consistency of layout**: Consistency of layout of symbols on pages, including when navigating through pages to select desired output, which can be:-
   - Inconsistent layout
   - Somewhat consistent layout
   - Highly consistent layout

Imagine you had to choose between only these three systems. You should indicate which you would prescribe for the child described. If your preferred option is not available, pick the system from the three options that you think best matches the child's needs. There are no right or wrong answers. It is acknowledged that this may feel uncomfortable for you.

In the survey, there are three different children described. You will be asked four questions about each child (12 questions in total).

In acknowledgement of choices being uncomfortable, after each choice, you will be asked to indicate how well you think that system matches the child's needs. (1 = very unsuitable, 7 = very suitable).

This survey is part of independent research funded by the National Institute for Health Research (NIHR), Health Service and Delivery Research (HS&DR) Programme 14/70/153. The views expressed are those of the authors and do not necessarily reflect those of the NHS, the NIHR, NETSCC, the HS&DR programme or the Department of Health.

Consent

For peer review only

Your participation in this survey is voluntary. All information is collected anonymously and held in confidence. We hope you complete the survey but you are free to stop responding at any point resulting in your answers will be removed.

❑ I have read and understood the above and consent to taking part.

I confirm my work involves assessing children for aided AAC systems and I contribute to the decision making in relation to the language and vocabulary organisation within aided AAC systems.

❑ Yes

❑ No

If yes go to DCE questions.

If no go to a page with the following:-

Thank you for your interest in this survey. At present we are only recruiting participants who contribute to decision making in relation to the language and vocabulary organisation within aided AAC for children. Over the coming 12 months we will be recruiting people with a wider range of AAC experience to test decision making resources we are developing. If you are interested in this aspect of the project or would like to be notified when the free resources are available, there will be an opportunity at the end to submit your email address.

We would still like to ask you a few questions about your experience with AAC to check the representativeness of participants.

Then go directly to demographics questionnaire.

For peer review only

Question 1

Child A has delayed expressive and receptive language and is able to use aided AAC for a few communicative functions. Child A is motivated to communicate through symbol communication systems. Child A is predicted to regress in skills and abilities (regression).

	System 1	System 2	System 3
**Size of vocabulary**<br>The size of the output vocabulary available within the aided AAC system.	50-1000 vocabulary items	50-1000 vocabulary items	50-1000 vocabulary items
**Type of vocabulary organisation**<br>Primary format used to organise the vocabulary within the aided AAC system	Pragmatic	Visual Scene	Visual Scene
**Vocabulary sets**<br>Pre-determined vocabulary or language package provided	No vocabulary set	Vocabulary sets with staged progression	Fixed vocabulary set
**Graphic representation**<br>Primary type of graphic symbol used	Ideographic symbol system (with rules or encoding)	Photos	Ideographic symbol system (with rules or encoding)
**Type of vocabulary organisation**<br>Primary format used to organise the vocabulary within the aided AAC system	Idiosyncratic layout	Idiosyncratic layout	Consistency of some aspects of layout

For this child I would choose:

- ❑ System 1
- ❑ System 2
- ❑ System 3

On a scale from 1 to 7, how good a match is your chosen device for this child? (1=very unsuitable, 7=very suitable).

❑	❑	❑	❑	❑	❑	❑
1	2	3	4	5	6	7

For peer review only

Question 2

Child A has delayed expressive and receptive language and is able to use aided AAC for a few communicative functions. Child A is motivated to communicate through symbol communication systems. Child A is predicted to regress in skills and abilities (regression).

	**System 1**	**System 2**	**System 3**
**Size of vocabulary**<br>The size of the output vocabulary available within the aided AAC system.	50-1000 vocabulary items	50-1000 vocabulary items	50-1000 vocabulary items
**Type of vocabulary organisation**<br>Primary format used to organise the vocabulary within the aided AAC system	Taxonomic	Visual Scene	Pragmatic
**Vocabulary sets**<br>Pre-determined vocabulary or language package provided	Vocabulary sets with staged progression	No vocabulary set	Fixed vocabulary set
**Graphic representation**<br>Primary type of graphic symbol used	Text	Pictographic symbol set	Photos
**Type of vocabulary organisation**<br>Primary format used to organise the vocabulary within the aided AAC system	Consistency of some aspects of layout	Idiosyncratic layout	Consistency of some aspects of layout

For this child I would choose:
- ❑ System 1
- ❑ System 2
- ❑ System 3

On a scale from 1 to 7, how good a match is your chosen device for this child? (1=very unsuitable, 7=very suitable).

❑	❑	❑	❑	❑	❑	❑
1	2	3	4	5	6	7

For peer review only

Question 3

Child A has delayed expressive and receptive language and is able to use aided AAC for a few communicative functions. Child A is motivated to communicate through symbol communication systems. Child A is predicted to regress in skills and abilities (regression).

	System 1	System 2	System 3
**Size of vocabulary**<br>The size of the output vocabulary available within the aided AAC system.	50-1000 vocabulary items	50-1000 vocabulary items	Up to 50 vocabulary items
**Type of vocabulary organisation**<br>Primary format used to organise the vocabulary within the aided AAC system	Semantic-Syntactic	Semantic-Syntactic	Visual Scene
**Vocabulary sets**<br>Pre-determined vocabulary or language package provided	Vocabulary sets with staged progression	No vocabulary set	Fixed vocabulary set
**Graphic representation**<br>Primary type of graphic symbol used	Text	Photos	Pictographic symbol set
**Type of vocabulary organisation**<br>Primary format used to organise the vocabulary within the aided AAC system	Consistency of all aspects of layout	Idiosyncratic layout	Consistency of some aspects of layout

For this child I would choose:

- ❑ System 1
- ❑ System 2
- ❑ System 3

On a scale from 1 to 7, how good a match is your chosen device for this child? (1=very unsuitable, 7=very suitable).

❑	❑	❑	❑	❑	❑	❑
1	2	3	4	5	6	7

For peer review only

Question 4

Child A has delayed expressive and receptive language and is able to use aided AAC for a few communicative functions. Child A is motivated to communicate through symbol communication systems. Child A is predicted to regress in skills and abilities (regression).

	**System 1**	**System 2**	**System 3**
**Size of vocabulary** The size of the output vocabulary available within the aided AAC system.	50-1000 vocabulary items	50-1000 vocabulary items	50-1000 vocabulary items
**Type of vocabulary organisation** Primary format used to organise the vocabulary within the aided AAC system	Visual Scene	Taxonomic	Pragmatic
**Vocabulary sets** Pre-determined vocabulary or language package provided	Vocabulary sets with staged progression	Fixed vocabulary set	Fixed vocabulary set
**Graphic representation** Primary type of graphic symbol used	Ideographic symbol system (with rules or encoding)	Text	Photos
**Type of vocabulary organisation** Primary format used to organise the vocabulary within the aided AAC system	Consistency of some aspects of layout	Consistency of all aspects of layout	Idiosyncratic layout

For this child I would choose:
- ❑ System 1
- ❑ System 2
- ❑ System 3

On a scale from 1 to 7, how good a match is your chosen device for this child? (1=very unsuitable, 7=very suitable).

❑	❑	❑	❑	❑	❑	❑
1	2	3	4	5	6	7

For peer review only

Question 5

Child B has receptive language exceeding expressive language and no previous AAC experience. Child B is only motivated to communicate through methods other than symbol communication systems. Child B is predicted to maintain current skills and abilities (plateau).

	**System 1**	**System 2**	**System 3**
**Size of vocabulary** The size of the output vocabulary available within the aided AAC system.	Up to 50 vocabulary items	50-1000 vocabulary items	More than 1000 vocabulary items
**Type of vocabulary organisation** Primary format used to organise the vocabulary within the aided AAC system	Taxonomic	Visual Scene	Pragmatic
**Vocabulary sets** Pre-determined vocabulary or language package provided	No vocabulary set	Fixed vocabulary set	Vocabulary sets with staged progression
**Graphic representation** Primary type of graphic symbol used	Photos	Text	Ideographic symbol system (with rules or encoding)
**Type of vocabulary organisation** Primary format used to organise the vocabulary within the aided AAC system	Consistency of all aspects of layout	Idiosyncratic layout	Consistency of some aspects of layout

For this child I would choose:

- ❑ System 1
- ❑ System 2
- ❑ System 3

On a scale from 1 to 7, how good a match is your chosen device for this child? (1=very unsuitable, 7=very suitable).

❑ 1 ❑ 2 ❑ 3 ❑ 4 ❑ 5 ❑ 6 ❑ 7

For peer review only

Question 6

Child B has receptive language exceeding expressive language and no previous AAC experience. Child B is only motivated to communicate through methods other than symbol communication systems. Child B is predicted to maintain current skills and abilities (plateau).

	**System 1**	**System 2**	**System 3**
**Size of vocabulary** The size of the output vocabulary available within the aided AAC system.	More than 1000 vocabulary items	Up to 50 vocabulary items	50-1000 vocabulary items
**Type of vocabulary organisation** Primary format used to organise the vocabulary within the aided AAC system	Semantic-Syntactic	Semantic-Syntactic	Taxonomic
**Vocabulary sets** Pre-determined vocabulary or language package provided	Vocabulary sets with staged progression	Fixed vocabulary set	No vocabulary set
**Graphic representation** Primary type of graphic symbol used	Ideographic symbol system (with rules or encoding)	Photos	Ideographic symbol system (with rules or encoding)
**Type of vocabulary organisation** Primary format used to organise the vocabulary within the aided AAC system	Idiosyncratic layout	Consistency of some aspects of layout	Consistency of some aspects of layout

For this child I would choose:

- ❑ System 1
- ❑ System 2
- ❑ System 3

On a scale from 1 to 7, how good a match is your chosen device for this child? (1=very unsuitable, 7=very suitable).

❑	❑	❑	❑	❑	❑	❑
1	2	3	4	5	6	7

For peer review only

Question 7

Child B has receptive language exceeding expressive language and no previous AAC experience. Child B is only motivated to communicate through methods other than symbol communication systems. Child B is predicted to maintain current skills and abilities (plateau).

	**System 1**	**System 2**	**System 3**
**Size of vocabulary**<br>The size of the output vocabulary available within the aided AAC system.	Up to 50 vocabulary items	More than 1000 vocabulary items	50-1000 vocabulary items
**Type of vocabulary organisation**<br>Primary format used to organise the vocabulary within the aided AAC system	Taxonomic	Pragmatic	Taxonomic
**Vocabulary sets**<br>Pre-determined vocabulary or language package provided	Fixed vocabulary set	Vocabulary sets with staged progression	No vocabulary set
**Graphic representation**<br>Primary type of graphic symbol used	Photos	Ideographic symbol system (with rules or encoding)	Text
**Type of vocabulary organisation**<br>Primary format used to organise the vocabulary within the aided AAC system	Consistency of all aspects of layout	Consistency of some aspects of layout	Idiosyncratic layout

For this child I would choose:

- ❑ System 1
- ❑ System 2
- ❑ System 3

On a scale from 1 to 7, how good a match is your chosen device for this child? (1=very unsuitable, 7=very suitable).

❑	❑	❑	❑	❑	❑	❑
1	2	3	4	5	6	7

For peer review only

Question 8

Child B has receptive language exceeding expressive language and no previous AAC experience. Child B is only motivated to communicate through methods other than symbol communication systems. Child B is predicted to maintain current skills and abilities (plateau).

	System 1	System 2	System 3
**Size of vocabulary**<br>The size of the output vocabulary available within the aided AAC system.	Up to 50 vocabulary items	More than 1000 vocabulary items	50-1000 vocabulary items
**Type of vocabulary organisation**<br>Primary format used to organise the vocabulary within the aided AAC system	Pragmatic	Semantic-Syntactic	Visual Scene
**Vocabulary sets**<br>Pre-determined vocabulary or language package provided	Fixed vocabulary set	Fixed vocabulary set	Vocabulary sets with staged progression
**Graphic representation**<br>Primary type of graphic symbol used	Ideographic symbol system (with rules or encoding)	Ideographic symbol system (with rules or encoding)	Photos
**Type of vocabulary organisation**<br>Primary format used to organise the vocabulary within the aided AAC system	Consistency of some aspects of layout	Consistency of all aspects of layout	Idiosyncratic layout

For this child I would choose:

- ❑ System 1
- ❑ System 2
- ❑ System 3

On a scale from 1 to 7, how good a match is your chosen device for this child? (1=very unsuitable, 7=very suitable).

❑	❑	❑	❑	❑	❑	❑
1	2	3	4	5	6	7

For peer review only

Question 9

Child C has delayed expressive and receptive language and no previous AAC experience. Child C is only motivated to communicate through methods other than symbol communication systems. Child C is predicted to maintain current skills and abilities (plateau).

	System 1	System 2	System 3
**Size of vocabulary**<br>The size of the output vocabulary available within the aided AAC system.	50-1000 vocabulary items	More than 1000 vocabulary items	Up to 50 vocabulary items
**Type of vocabulary organisation**<br>Primary format used to organise the vocabulary within the aided AAC system	Visual Scene	Pragmatic	Semantic-Syntactic
**Vocabulary sets**<br>Pre-determined vocabulary or language package provided	Fixed vocabulary set	Fixed vocabulary set	Fixed vocabulary set
**Graphic representation**<br>Primary type of graphic symbol used	Pictographic symbol set	Pictographic symbol set	Text
**Type of vocabulary organisation**<br>Primary format used to organise the vocabulary within the aided AAC system	Idiosyncratic layout	Consistency of some aspects of layout	Consistency of all aspects of layout

For this child I would choose:
- ❑ System 1
- ❑ System 2
- ❑ System 3

On a scale from 1 to 7, how good a match is your chosen device for this child? (1=very unsuitable, 7=very suitable).

❑	❑	❑	❑	❑	❑	❑
1	2	3	4	5	6	7

For peer review only

Question 10

Child C has delayed expressive and receptive language and no previous AAC experience. Child C is only motivated to communicate through methods other than symbol communication systems. Child C is predicted to maintain current skills and abilities (plateau).

	System 1	System 2	System 3
**Size of vocabulary**<br>The size of the output vocabulary available within the aided AAC system.	Up to 50 vocabulary items	More than 1000 vocabulary items	Up to 50 vocabulary items
**Type of vocabulary organisation**<br>Primary format used to organise the vocabulary within the aided AAC system	Taxonomic	Semantic-Syntactic	Taxonomic
**Vocabulary sets**<br>Pre-determined vocabulary or language package provided	No vocabulary set	Vocabulary sets with staged progression	No vocabulary set
**Graphic representation**<br>Primary type of graphic symbol used	Ideographic symbol system (with rules or encoding)	Ideographic symbol system (with rules or encoding)	Photos
**Type of vocabulary organisation**<br>Primary format used to organise the vocabulary within the aided AAC system	Consistency of all aspects of layout	Consistency of some aspects of layout	Idiosyncratic layout

For this child I would choose:
- ❑ System 1
- ❑ System 2
- ❑ System 3

On a scale from 1 to 7, how good a match is your chosen device for this child? (1=very unsuitable, 7=very suitable).

❑	❑	❑	❑	❑	❑	❑
1	2	3	4	5	6	7

For peer review only

Question 11

Child C has delayed expressive and receptive language and no previous AAC experience. Child C is only motivated to communicate through methods other than symbol communication systems. Child C is predicted to maintain current skills and abilities (plateau).

	System 1	System 2	System 3
**Size of vocabulary**<br>The size of the output vocabulary available within the aided AAC system.	Up to 50 vocabulary items	50-1000 vocabulary items	50-1000 vocabulary items
**Type of vocabulary organisation**<br>Primary format used to organise the vocabulary within the aided AAC system	Visual Scene	Semantic-Syntactic	Semantic-Syntactic
**Vocabulary sets**<br>Pre-determined vocabulary or language package provided	No vocabulary set	Vocabulary sets with staged progression	No vocabulary set
**Graphic representation**<br>Primary type of graphic symbol used	Photos	Pictographic symbol set	Text
**Type of vocabulary organisation**<br>Primary format used to organise the vocabulary within the aided AAC system	Idiosyncratic layout	Consistency of some aspects of layout	Consistency of all aspects of layout

For this child I would choose:

- ❑ System 1
- ❑ System 2
- ❑ System 3

On a scale from 1 to 7, how good a match is your chosen device for this child? (1=very unsuitable, 7=very suitable).

❑	❑	❑	❑	❑	❑	❑
1	2	3	4	5	6	7

For peer review only

Question 12

Child C has delayed expressive and receptive language and no previous AAC experience. Child C is only motivated to communicate through methods other than symbol communication systems. Child C is predicted to maintain current skills and abilities (plateau).

	**System 1**	**System 2**	**System 3**
**Size of vocabulary**<br>The size of the output vocabulary available within the aided AAC system.	Up to 50 vocabulary items	More than 1000 vocabulary items	50-1000 vocabulary items
**Type of vocabulary organisation**<br>Primary format used to organise the vocabulary within the aided AAC system	Semantic-Syntactic	Semantic-Syntactic	Visual Scene
**Vocabulary sets**<br>Pre-determined vocabulary or language package provided	No vocabulary set	Vocabulary sets with staged progression	Fixed vocabulary set
**Graphic representation**<br>Primary type of graphic symbol used	Ideographic symbol system (with rules or encoding)	Text	Photos
**Type of vocabulary organisation**<br>Primary format used to organise the vocabulary within the aided AAC system	Consistency of all aspects of layout	Idiosyncratic layout	Consistency of some aspects of layout

For this child I would choose:

- ❑ System 1
- ❑ System 2
- ❑ System 3

On a scale from 1 to 7, how good a match is your chosen device for this child? (1=very unsuitable, 7=very suitable).

❑	❑	❑	❑	❑	❑	❑
1	2	3	4	5	6	7

For peer review only

Questionnaire

In this final part of the survey, we'd like to ask you for some information about yourself and your experience with AAC.

We would like to know about the characteristics of the people who complete this survey to check that we have a representative sample. We would also like to check if people with different professional experiences have different opinions.

All responses will be held anonymously and we have no way of tracing your responses back to you as an individual.

Q. What is your age? _______ years.

Q. What is your gender?

❑ Male ❑ Female ❑ Other ❑ Prefer not to say

Q. How would you describe your ethnicity?

- ❑ White - English/Welsh/Scottish/Northern Irish/British
- ❑ White -Irish
- ❑ White - Gypsy or Irish Traveller
- ❑ White - Any other White background
- ❑ Mixed/Multiple ethnic group - White and Black Caribbean
- ❑ Mixed/Multiple ethnic group - White and Black African
- ❑ Mixed/Multiple ethnic group - White and Asian
- ❑ Mixed/Multiple ethnic group - Any other Mixed/Multiple ethnic background
- ❑ Asian/Asian British - Indian
- ❑ Asian/Asian British - Pakistani
- ❑ Asian/Asian British – Bangladeshi
- ❑ Asian/Asian British - Chinese
- ❑ Asian/Asian British - Any other Asian background
- ❑ Black/ African/Caribbean/Black British - African

For peer review only

- ❑ Black/ African/Caribbean/Black British - Caribbean
- ❑ Black/ African/Caribbean/Black British - Any other Black/African/Caribbean background
- ❑ Arab
- ❑ Other

Q. For how many years have you worked with AAC? _______ years.

Q. What is your professional background? You may select more than one option if applicable.

- ❑ Occupational therapist
- ❑ Speech and language therapist
- ❑ Assistive technology specialist
- ❑ Clinical scientist
- ❑ Teacher
- ❑ Other

Q. If you selected Other, please specify.

____

Q. How much of your role relates to AAC? _______%.

(e.g. 1 day per week = 20%, 2 days a week = 40%, etc.)

Q. How would you characterise your role? Pick the one that best describes your role.

- ❑ I refer on anyone who may benefit from AAC
- ❑ I assess and implement AAC. I seek support from within my own team for decisions made
- ❑ I assess and implement AAC. I seek support from outside my own team for
- ❑ decisions made
- ❑ I assess and implement AAC. I act as a support for others in relation to AAC
- ❑ decision making
- ❑ I assess only. I provide support to others outside my team in relation to
- ❑ AAC decision making
- ❑ Other

Q. If you selected Other, please specify.

____

For peer review only

Q. Out of the list below, select the three most common diagnoses you encounter in your work.

- ❑ Autism Spectrum Disorder
- ❑ Physical disability (e.g. neuromuscular, cerebral palsy etc.)
- ❑ Dyspraxia
- ❑ Intellectual Disability/Developmental Delay
- ❑ Neurological
- ❑ Specific Speech/Language Impairment
- ❑ Syndromes
- ❑ Unknown
- ❑ Other

Q. If you selected Other, please specify.

____

Q. Who do you provide services for? (Please choose all that apply.)

- ❑ All age groups
- ❑ Preschool age
- ❑ Primary school age
- ❑ Secondary school age
- ❑ Higher education
- ❑ Further education
- ❑ Adults
- ❑ Other

Q. If you selected Other, please specify.

____

Q. What is the geographical area covered by your service? (Please choose all that apply.

- ❑ North West England
- ❑ North East England
- ❑ Yorkshire and Humber
- ❑ West Midlands
- ❑ East Midlands
- ❑ East of England

For peer review only

- ❑ South West England
- ❑ South East England
- ❑ London
- ❑ Northern Ireland
- ❑ North Wales
- ❑ South Wales
- ❑ Mid-Wales
- ❑ Southern Scotland
- ❑ Central Scotland
- ❑ Northern Scotland
- ❑ Non-UK

End of survey

Thank you for your participation in this survey.

Your responses will contribute to the results of the I-ASC project and support the development of decision making resources for use in AAC assessments.

You can follow the progress of our research project on our website, on Facebook or on Twitter.

For peer review only

Figure A 1: Example discrete choice experiment task

Child B has delayed expressive and receptive language and able to use aided AAC for a few communicative functions. Child B is only motivated to communicate through methods other than symbol communication systems. Child B is predicted to maintain current skills and abilities (plateau).

	System 1	System 2	System 3
Graphic Representation<br>Primary type of graphic symbol used	Ideographic symbols	Photo symbols	Pictographic symbols
Consistency of layout<br>Consistency of layout of symbols on pages, including when navigating through pages to select desired output.	Highly consistent layout	Somewhat consistent layout	Inconsistent layout
Vocabulary sets<br>Pre-determined vocabulary or language package provided	Commercially provided sets without language progression	Commercially provided sets with language progression	Commercially provided sets without language progression
Size of vocabulary<br>The size of the output vocabulary available within the aided AAC system.	More than 1000 vocabulary items	50-1000 vocabulary items	Up to 50 vocabulary items
Type of vocabulary organisation<br>Primary format used to organise the vocabulary within the aided AAC system	Pragmatic organisation	Semantic syntactic organisation	Visual scene display
For this child I would choose:	○	○	○

On a scale from 1 to 7, how good a match is your chosen device for this child? (1=very unsuitable, 7=very suitable)

1 = very unsuitable	2	3	4	5	6	7 = very suitable
○	○	○	○	○	○	○

35%

Appendix C – Final preferred model selection process

A full model with all interaction terms and two alternative specific constants implies 98 parameters, which is too many to reliably estimate given the amount of data collected and given that many interactions are expected to be of very low magnitude. Thus, a strategy was required to identify a suitable model with fewer parameters.

The first stage was estimating a series of stepwise multinomial logit (MNL) models, beginning with a model with all 98 parameters. The parameter with the highest p-value, excluding the γ_0 constant terms, was eliminated, and a model with 97 parameters was estimated. Then the parameter with the lowest p-value was excluded and a new model run, and so on in an iterative process until only the 12 γ_0 constant terms remained (one for each non-baseline system level).

The Bayesian Information Criterion (BIC) was used to select the preferred MNL model. This model was then re-estimated as a mixed logit (MIXL) model to account for participant heterogeneity. (The process did not begin by estimating a series of stepwise MIXL models due to the difficulty and greatly increased computational resources required to estimate MIXL models with a large number of parameters.) The β \coefficients on system attribute levels were assumed to be drawn from normal distributions with means given by

$$\bar{\beta}_{alc} = \gamma_{al0} + \gamma_{al} z_c$$

and variances given by

$$\sigma_{alc}^2 = (\sigma_{al0} + \sigma_{al} z_c)^2.$$

If p is the number of parameters of the preferred MNL model, then models with between $p - 3$ and $p + 3$ parameters were re-estimated as MIXL models. The BIC for each MIXL model is given in **Error! Reference source not found.**.

The MIXL model minimising the BIC was chosen as the final preferred model.

For peer review only

Table C 1: Bayesian information criteria (BIC) for estimated mixed logit models

Number of parameters	BIC
22	3502.25
23	3487.80
24	3482.30
25	3489.18
26	3493.07
27	3502.28
28	3509.34

For peer review only

Appendix D – Relative interaction attribute importance

Relative information attribute importance (RIAI) measures the amount that preferences for attributes of choice objects are impacted by a given interaction attribute associated with a choice situation relative to other interaction attributes. It may be calculated either with respect to a single choice object attribute or overall with respect to all choice object attributes.

RIAI is calculated with respect to a single choice object attribute by taking the difference between the greatest increase an interaction attribute causes to a choice object attribute's part worth utility and the greatest decrease, expressed as a percentage of the differences for all interaction attributes. Formally, the RIAI for interaction attribute i with respect to choice attribute c is

$$RIAI_{ic} = 100\left(\frac{\gamma_{ic}^{max} - \gamma_{ic}^{min}}{\sum_{j=1}^{N_I} \gamma_{jc}^{max} - \gamma_{jc}^{min}}\right)$$

where γ_{ic}^{max} and γ_{ic}^{min} are respectively the maximum and minimum coefficients for interaction attribute i with respect to choice attribute c and N_I is the number of interaction attributes. The overall RIAI for i is similarly calculated as

$$RIAI_{i} = 100\left(\frac{\gamma_{i}^{max} - \gamma_{i}^{min}}{\sum_{j=1}^{N_I} \gamma_{j}^{max} - \gamma_{j}^{min}}\right)$$

Where now γ_{i}^{max} and γ_{i}^{min} are respectively the maximum and minimum coefficients for interaction attribute i across all choice attributes.

For peer review only

STROBE Statement—checklist of items that should be included in reports of observational studies

	Item No.	Recommendation	Page No.	Relevant text from manuscript
**Title and abstract**	1	(*a*) Indicate the study's design with a commonly used term in the title or the abstract	Page 1	…using a discrete choice experiment…
		(*b*) Provide in the abstract an informative and balanced summary of what was done and what was found	Page 2	
**Introduction**				
Background/rationale	2	Explain the scientific background and rationale for the investigation being reported	Pages 3-4	
Objectives	3	State specific objectives, including any prespecified hypotheses	Page 5	The current study sought to complement the previous work by examining fewer factors in more detail using a DCE.43 It aimed to quantify the clinical judgements and trade-offs AAC professionals make between different attributes of AAC systems, and how those trade-offs change depending on children's characteristics, things not possible using BWS case 1. This is the first DCE carried out in AAC, and there were challenges associated with performing a DCE with a target population of AAC professionals (for details see discussion). Thus, an additional goal was to establish the

For peer review only

For peer review only - http://bmjopen.bmj.com/site/about/guidelines.xhtml

				feasibility of using DCEs as a research tool in AAC.
**Methods**				
Study design	4	Present key elements of study design early in the paper	Pages 6-7	
Setting	5	Describe the setting, locations, and relevant dates, including periods of recruitment, exposure, follow-up, and data collection	Setting and locations: Page 7<br><br>Relevant dates: Page 7	The DCE was administered online for ease of recruitment.<br><br>Responses were collected between 20/10/17 and 4/3/18.
Participants	6	(*a*) *Cohort study*—Give the eligibility criteria, and the sources and methods of selection of participants. Describe methods of follow-up<br>*Case-control study*—Give the eligibility criteria, and the sources and methods of case ascertainment and control selection. Give the rationale for the choice of cases and controls<br>*Cross-sectional study*—Give the eligibility criteria, and the sources and methods of selection of participants	Eligibility criteria: Page 8<br><br>Sources and methods of selection of participants: Page 7	Participants began by confirming they contributed towards AAC decision-making for children<br>Recruitment was carried out via AAC professionals' email distribution lists (the project's own list and the mailing list of the UK wide charity Communication Matters). In addition, invitations were sent via publicly available lists and websites, and the professional contacts of authors. Adverts were also placed on the project website and online media.
		(*b*) *Cohort study*—For matched studies, give matching criteria and number of exposed and unexposed<br>*Case-control study*—For matched studies, give matching criteria and the number of controls per case		

For peer review only - http://bmjopen.bmj.com/site/about/guidelines.xhtml

Variables	7	Clearly define all outcomes, exposures, predictors, potential confounders, and effect modifiers. Give diagnostic criteria, if applicable	Pages 8-9	
Data sources/ measurement	8*	For each variable of interest, give sources of data and details of methods of assessment (measurement). Describe comparability of assessment methods if there is more than one group	Appendix B	
Bias	9	Describe any efforts to address potential sources of bias	Pages 8-9	…where α_s was an alternative specific constant for AAC system s… … after adjusting for multiple testing using Holm's sequential Bonferroni correction.
Study size	10	Explain how the study size was arrived at	Page 7	Prior experience from the BWS study suggested it would be difficult to recruit a large respondent sample, so to maximise the information captured a relatively heavy response burden of 12 choices between three systems was selected for the DCE.

Continued on next page

For peer review only

Quantitative variables	11	Explain how quantitative variables were handled in the analyses. If applicable, describe which groupings were chosen and why	Pages 8-9	
Statistical methods	12	(*a*) Describe all statistical methods, including those used to control for confounding	Pages 8-9	
		(*b*) Describe any methods used to examine subgroups and interactions		
		(*c*) Explain how missing data were addressed		
		(*d*) *Cohort study*—If applicable, explain how loss to follow-up was addressed<br>*Case-control study*—If applicable, explain how matching of cases and controls was addressed<br>*Cross-sectional study*—If applicable, describe analytical methods taking account of sampling strategy		
		(*e*) Describe any sensitivity analyses		
**Results**				
Participants	13*	(a) Report numbers of individuals at each stage of study—eg numbers potentially eligible, examined for eligibility, confirmed eligible, included in the study, completing follow-up, and analysed	Page 10	A total of 172 participants completed the survey, of whom 155 indicated they contributed to decision-making regarding AAC systems and answered DCE questions.
		(b) Give reasons for non-participation at each stage		
		(c) Consider use of a flow diagram		
Descriptive data	14*	(a) Give characteristics of study participants (eg demographic, clinical, social) and information on exposures and potential confounders	Table 3	
		(b) Indicate number of participants with missing data for each variable of interest		
		(c) *Cohort study*—Summarise follow-up time (eg, average and total amount)		
Outcome data	15*	*Cohort study*—Report numbers of outcome events or summary measures over time	Table 4, Figure 1	
		Case-control study—Report numbers in each exposure category, or summary measures of exposure		
		Cross-sectional study—Report numbers of outcome events or summary measures		
Main results	16	(*a*) Give unadjusted estimates and, if applicable, confounder-adjusted estimates and their precision (eg, 95% confidence interval). Make clear which confounders were adjusted for and why they were included	Table 4, Figure 1	
		(*b*) Report category boundaries when continuous variables were categorized		

For peer review only - http://bmjopen.bmj.com/site/about/guidelines.xhtml

	(*c*) If relevant, consider translating estimates of relative risk into absolute risk for a meaningful time period

Continued on next page

For peer review only

Other analyses	17	Report other analyses done—eg analyses of subgroups and interactions, and sensitivity analyses		
**Discussion**				
Key results	18	Summarise key results with reference to study objectives	Pages 12-16	
Limitations	19	Discuss limitations of the study, taking into account sources of potential bias or imprecision. Discuss both direction and magnitude of any potential bias	Pages 16-17	
Interpretation	20	Give a cautious overall interpretation of results considering objectives, limitations, multiplicity of analyses, results from similar studies, and other relevant evidence	Pages 12-16	
Generalisability	21	Discuss the generalisability (external validity) of the study results	Pages 16-17	The DCE task may not match how UK AAC professionals make decisions in practice. Typically, many participants have the opportunity to work with families and children, as well as part of an AAC team, which could include diverse areas of clinical and personal expertise. Teams also generally make recommendations, rather than unilaterally choosing a system. However, there is evidence that AAC professionals compare the attributes of AAC systems in everyday practice,[13] and that they make trade-offs between system attributes,[37] akin to DCE tasks. In addition, it is still useful to study the individual decision-making of AAC professionals. Lynch et al.[30] reported that a wide variety of team structures are used, and the mode of service delivery can have an influence on outcomes. Gathering evidence on individual-level decision-making can thus inform an assessment of how different ways of organising services influence decisions.<br>The DCE tasks presented one-off static decisions made by a single individual. In reality the decision-making environment is dynamic,

For peer review only - http://bmjopen.bmj.com/site/about/guidelines.xhtml

				with children developing over time, and often having two or more devices over the course of their childhood. These differences are a limiting factor in the external validity of results.
**Other information**				
Funding	22	Give the source of funding and the role of the funders for the present study and, if applicable, for the original study on which the present article is based	Page 18	

*Give information separately for cases and controls in case-control studies and, if applicable, for exposed and unexposed groups in cohort and cross-sectional studies.

Note: An Explanation and Elaboration article discusses each checklist item and gives methodological background and published examples of transparent reporting. The STROBE checklist is best used in conjunction with this article (freely available on the Web sites of PLoS Medicine at http://www.plosmedicine.org/, Annals of Internal Medicine at http://www.annals.org/, and Epidemiology at http://www.epidem.com/). Information on the STROBE Initiative is available at www.strobe-statement.org.

For peer review only

For peer review only - http://bmjopen.bmj.com/site/about/guidelines.xhtml
